# Supplementary material for: Structural Basis for Broad HIV-1 Neutralization by the MPER-Specific Human Broadly Neutralizing Antibody LN01
Source: Cell Host Microbe. 2019 Nov 13;26(5):623–637.e8. doi: 10.1016/j.chom.2019.09.016 (PMC6854463; doi:10.1016/j.chom.2019.09.016)
Supplement: Document S1. Figures S1–S7 and Tables S1–S3 [file mmc1.pdf]

Cell Host & Microbe, Volume *26*

Supplemental Information

Structural Basis for Broad HIV-1 Neutralization by the MPER-Specific Human Broadly Neutralizing Antibody LN01

Dora Pinto, Craig Fenwick, Christophe Caillat, Chiara Silacci, Serafima Guseva, François Dehez, Christophe Chipot, Sonia Barbieri, Andrea Minola, David Jarrossay, Georgia D. Tomaras, Xiaoying Shen, Agostino Riva, Maciej Tarkowski, Olivier Schwartz, Timothée Bruel, Jérémy Dufloo, Michael S. Seaman, David C. Montefiori, Antonio Lanzavecchia, Davide Corti, Giuseppe Pantaleo, and Winfried Weissenhorn

Supplemental Information

Structural basis for broad HIV-1 neutralization by the MPER-specific human broadly neutralizing antibody LN01

Dora Pinto, Craig Fenwick, Christophe Caillat, Chiara Silacci, Serafima Guseva, François Dehez, Christophe Chipot, Sonia Barbieri, Andrea Minola, David Jarrossay, Georgia D. Tomaras, Xiaoying Shen, Agostino Riva, Maciej Tarkowski, Olivier Schwartz, Timothée Bruel, Jérémy Dufloo, Michael S. Seaman, David C. Montefiori, Antonio Lanzavecchia, Davide Corti, Giuseppe Pantaleo, Winfried Weissenhorn

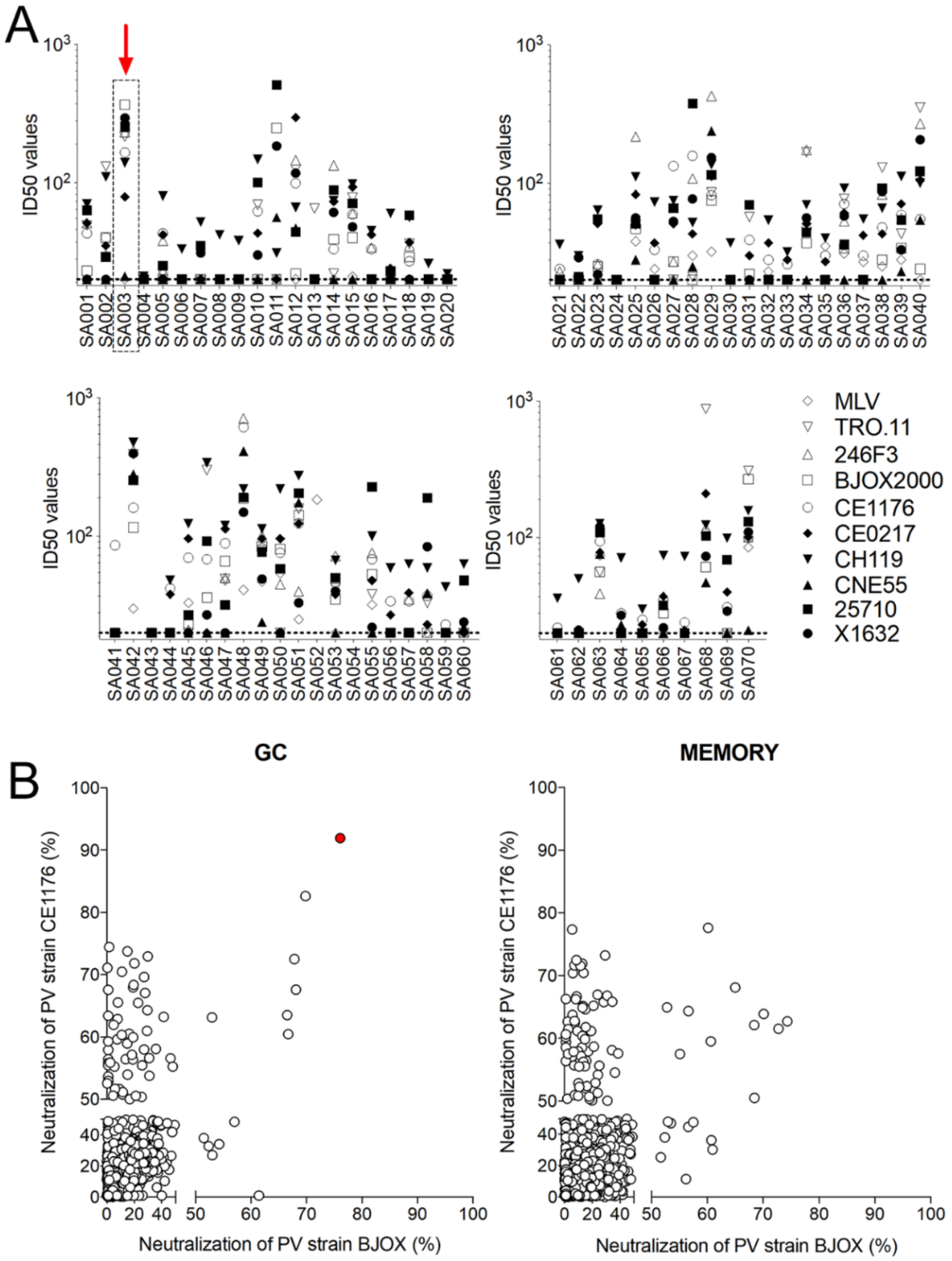

Figure S1 related to Figure 1. Selection of HIV-1 patients for the presence of broad neutralizing activity in the serum and identification of the LN01 mAb.

(A) A cohort (n=70) of patients chronically infected with HIV-1 clade B or clade C were screened for the presence of high titers of antibodies able to neutralize a panel of nine HIV-1 PVs from the Global Panel of HIV-1 reference strains. The sera were tested in neutralization assay using TZM-bl cells and the ID50s (y-axes) were determined.

(B) Identification of LN01 mAb. IgG GC and IgG memory B cells isolated from LNMC of an HIV-1 infected donor were seeded on MSC at 3 c/well in 384-well plates in the presence of IL-2, IL-6, IL-21, BCR-trigger and EBV. After 14 days of culture, supernatants were collected and tested for their ability to neutralize HIV-1 strains CE1176 and BJOX PV using a TZMbl-base micro-neutralization assay.

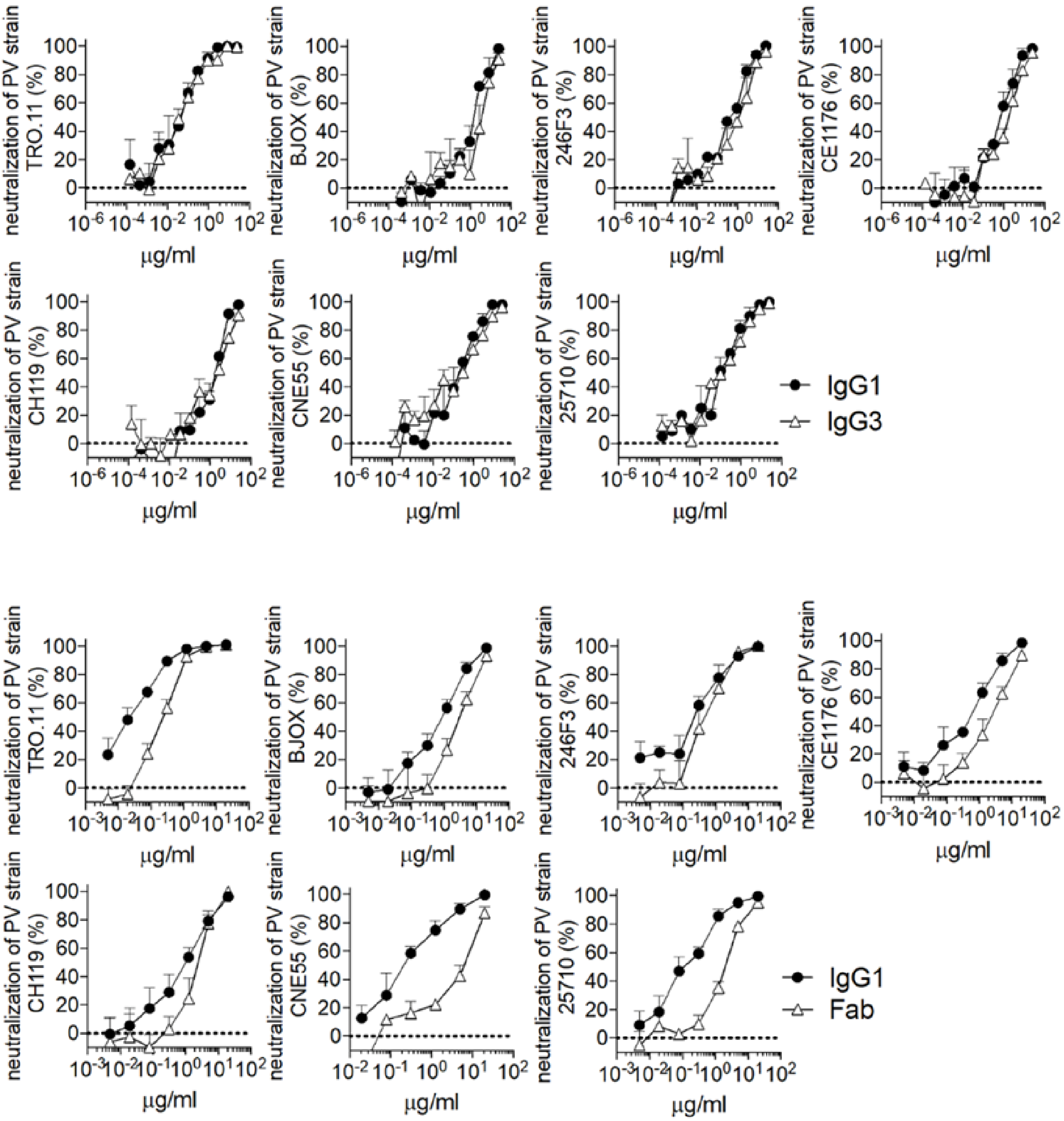

Figure S2 related to Figures 1C and 3A, B. Neutralizing activity of LN01 IgG1, IgG3 and Fab Ig formats.

Percentage of neutralization of LN01 IgG1 compared to LN01 IgG3 (top panel) and LN01 Fab fragment (bottom panel) against seven HIV-1 PVs of the Global Panel. Serial dilutions of the recombinant mAbs were incubated with HIV-1 PVs and then added to the TZM-bl target cells.

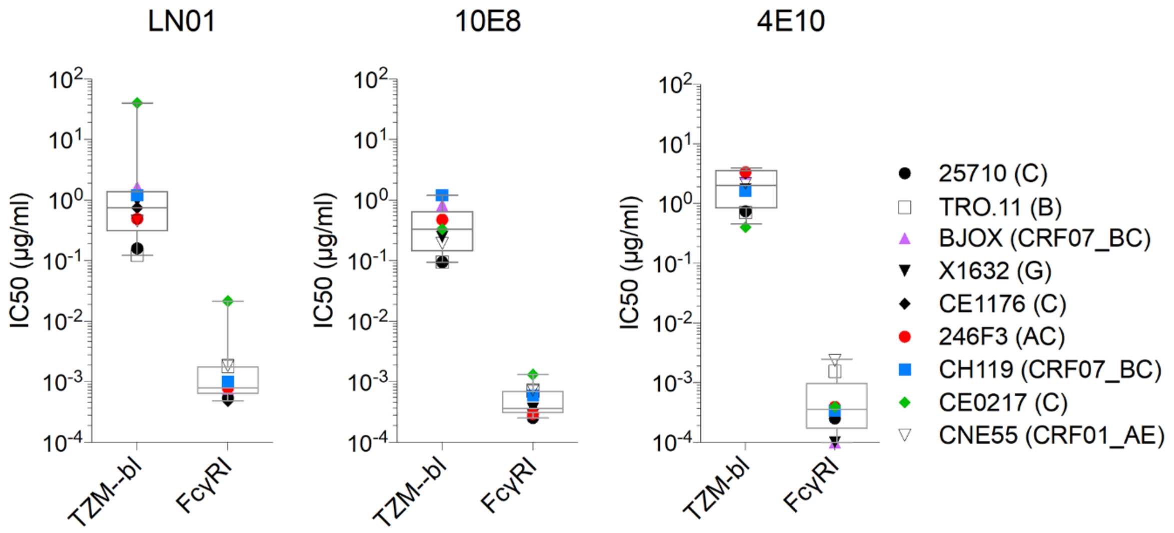

Figure S3 related to Figures 1C and 3A, B. FcγRI expression on target cells greatly enhances HIV-1 neutralization by anti-MPER broadly neutralizing mAbs.

LN01 mAb and the two other anti-MPER mAbs 10E8 and 4E10 were tested for their neutralizing activity against nine HIV-1 PVs of the Global Panel using either parental TZM-bl cells or TZM-bl cells expressing human FcγRI. The IC50 values expressed in µg/ml are shown.

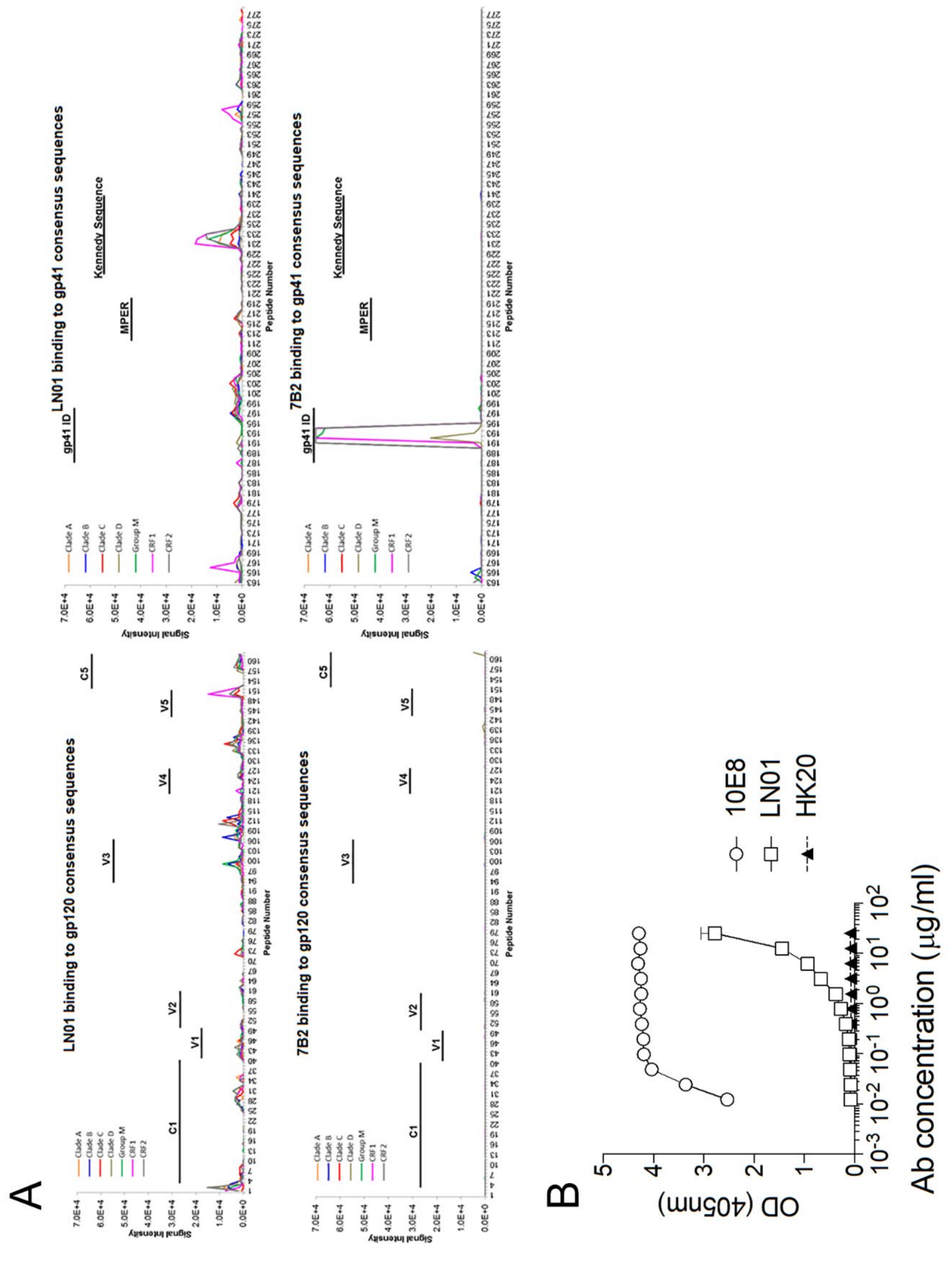

Figure S4 related to Figure 4. LN01 epitope specificity and binding.

(A) Binding of LN01 and 7B2 mAbs to an array of 1423 15-mer peptides, overlapping by 12 amino acids, that cover the full length of the consensus HIV-1 Env gp160 sequences for clades A, B, C, D, group M, CRF01_AE and CRF02_AG. Signals below 2.0E+4 are scored as negative. As expected 7B2 reacts with the gp41 immuno-dominant region.

(B) LN01, 10E8 and HK20 (HR1 specific) mAbs were tested in ELISA for binding to a 28 amino acids long peptide encompassing the entire gp41 MPER. Shown are the OD values at 405 nm.

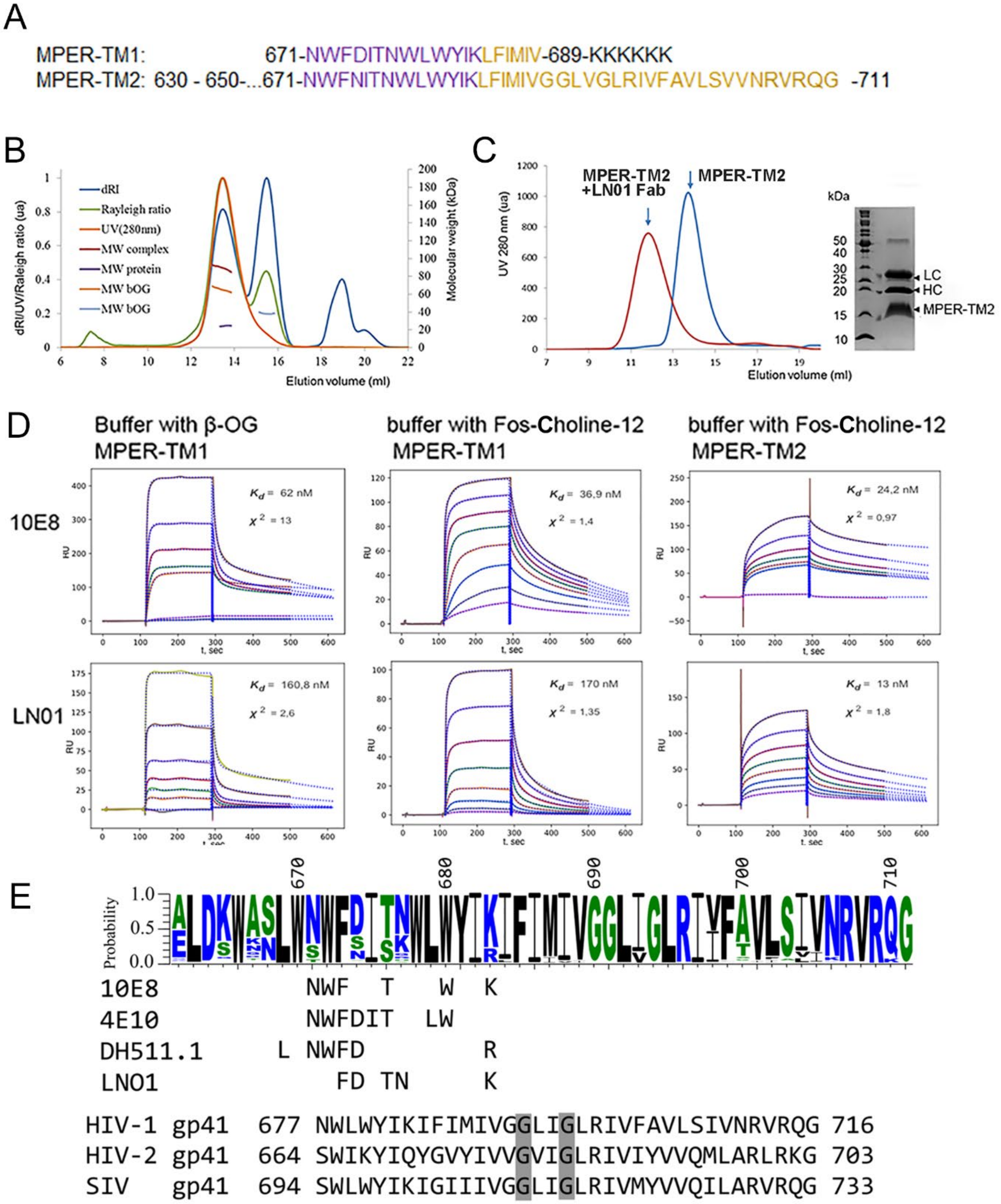

Figure S5 related to Figures 5 and 6. Sequence conservation of MPER and interaction of LN01 with gp41 peptides.

(A) Sequences of gp41 peptides, MPER-TM1 and MPER-TM-2 used in this study. Two forms of MPER-TM2 were expressed, gp41 residues 630 to 711, which was used for experiments

shown in D and MPER-TM2 residues 650 to 711 used for all other MPER-TM2 experiments. MPER-TM2 650 to 711 was generated by an engineered TEV site in the original 630 to 711 construct. The MPER sequence present in the structures is drawn in purple and the transmembrane region in beige.

(B) MPER-TM2 forms trimers in a buffer containing β-OG as determined by SEC-MALLS. The sample was applied to size-exclusion chromatography on a Superdex 200 column. The chromatogram shows detector readings of the light scattering detector (Rayleigh ratio), the UV_{280} detector, and the refractive index detector (dRI). The horizontal lines indicate the calculated mass of MPER-TM2 (24 kDa), detergent micelle in the complex (65 kDa), MPER-TM2-detergent complex (89 kDa) and free detergent micelle (38 kDa). The molecular weight of the MPER-TM2 (residues 650 to 711) monomer is 7.2 kDa.

(C) SEC analysis of MPER-TM2 alone and in complex with LN01 demonstrates complex formation in a buffer containing Fos-Choline-12. MPER-TM2 is monomeric in this buffer. The SDS-PAGE of a central fraction of the LN01-MPER-TM2 peak reveals the presence of MPER-TM2 and the Fab.

(D) SPR analyses of 10E8 and LN01 interaction with MPER-TM1 and MPER-TM2 in buffers containing β-OG and Fos-Choline-12. Injections were performed in the range of 1 to 2048 nM. The raw data is shown in full lines and the fits are drawn as dashed lines. The χ^2 of the fits and the calculated K_d are shown as insets.

(E) Sequence Conservation of gp41 MPER and the transmembrane domain (TM). Upper panel, comparison of 5448 sequences of HIV-1, M group (A-K plus recombinants) from http://www.hiv.lanl.gov. Middle panel, main residues interacting with bNAbs 4E10, 10E8, DH511.1 and LN01. Lower panel, consensus amino acid sequences of the TM region of HIV-1, HIV-2 and SIV showing the positions of the conserved glycine residues.

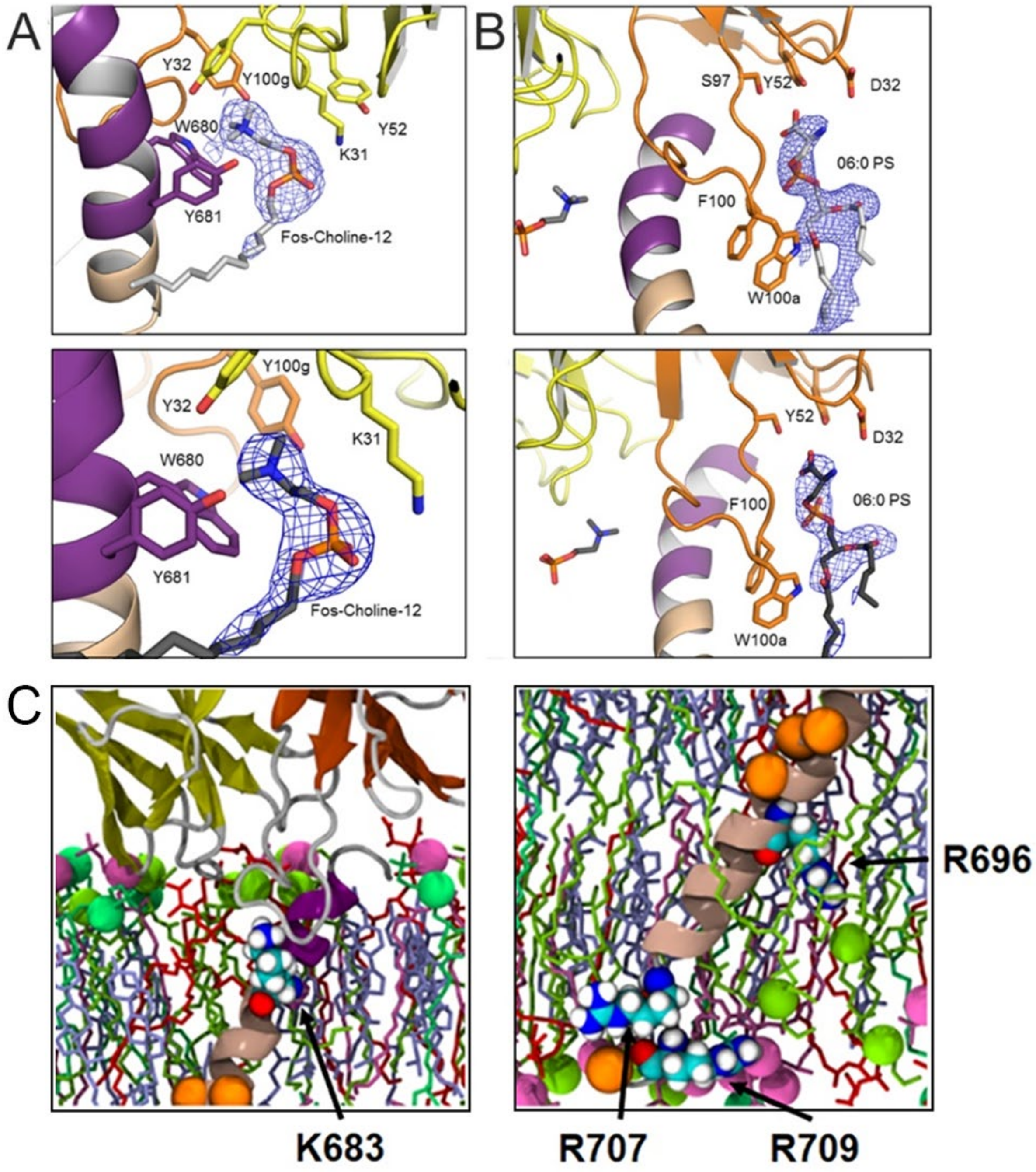

Figure S6 related to Figures 5B, 6 and 7. Molecular details of Fos-Choline-12 and 06:0 PS interaction and of transmembrane residues with lipid head groups. (A) Fos-Choline-12 and (B) 06:0 PS binding. The 2Fo-Fc composite omit map (1 σ level) is represented as a blue mesh around the ligands Fos-Choline-12 (A) and 06:0 PS (B). The 2Fo-Fc map (1σ level) calculated after refinement is represented as a blue mesh around the ligands Fos-Choline-12 (A lower panel) and 06:0 PS (B lower panel). All residues potentially interacting (within 3.5 Å) with the lipids are represented in sticks.

(C) Close-up of the LN01 Fab in complex with MPER-TM2 positioned in a lipid bilayer by MD simulation. The lipid bilayer is composed of POPC (turquoise), POPE (yellow-green), POPS (red), SSM (pink) and cholesterol (mauve). The lipid head groups are represented as van der

Waals spheres of the corresponding color. The light chain of the LN01 Fab is shown in yellow and the heavy chain in orange (left panel). Conserved basic residues positioned to contact lipid head groups on the Ab binding side of the bilayer are shown on the left panel and those present on the on the opposite side of the bilayer are shown on the right panel.

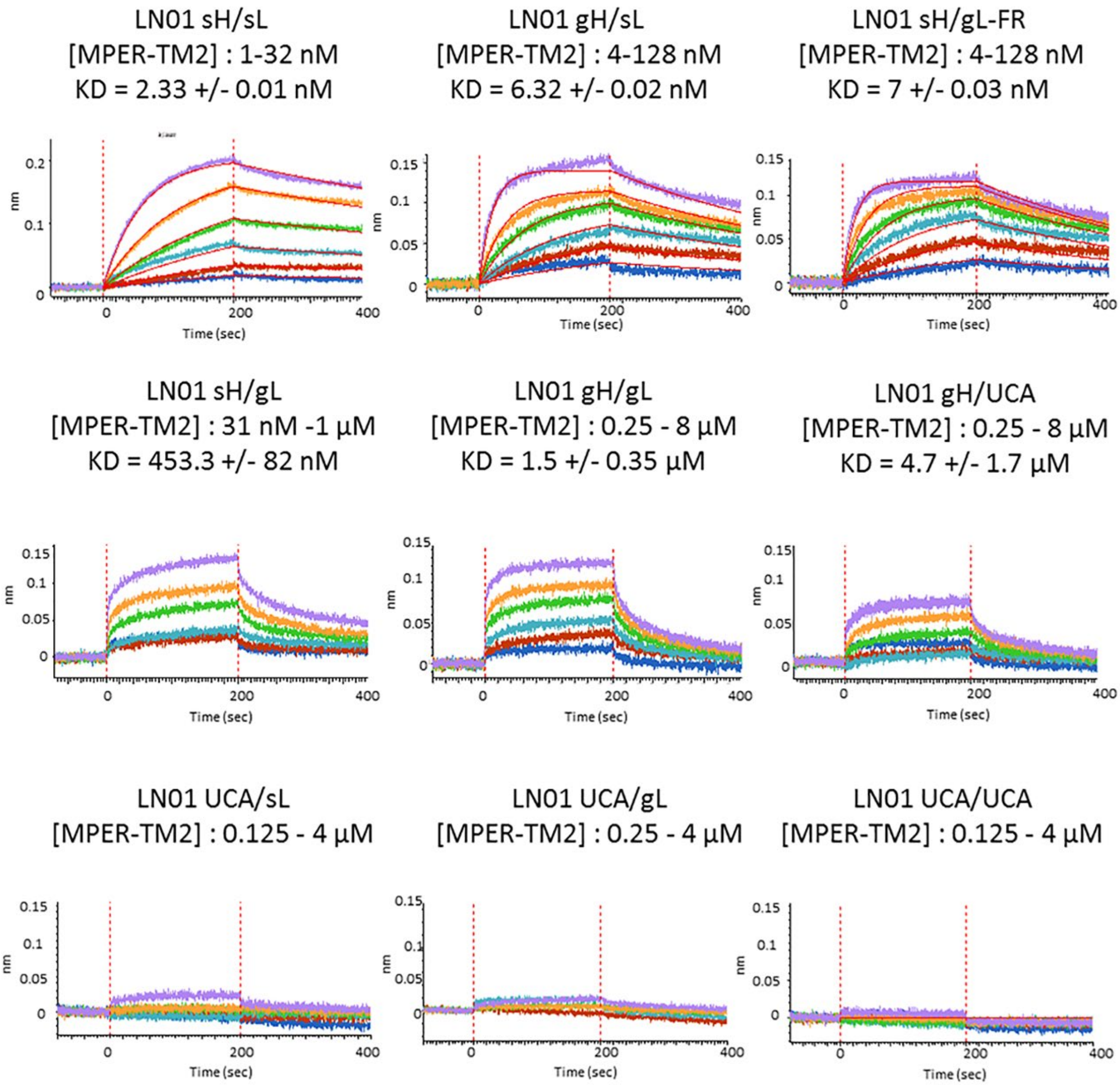

Figure S7 related to Figures 1D and E: Binding of LN01 variants to gp41 MPER-TM2 as measured by Bio-layer interferometry (BLI). Colored lines represent the response curves (blue is the lowest concentration and purple the highest). For LN01 sH/sL, LN01 gH/sL and LN01 sH/gL-FR the red line represents the best fit (1:1 model, see STAR*METHODS).

Table S1 related to Figures 2A, B. Breadth, and potency of LN01 mAb against 118 HIV-1 PVs. LN01 and 10E8 were tested in micro-neutralization assay on TZM-bl target cells against the extended panel of 118 HIV-1 PVs. Shown are the IC50 and IC80 values. Color code: red < 1 µg/ml; orange 1-10 µg/ml; yellow > 10 µg/ml.

		Titer in TZM-bl cells (ug/ml)				
		IC50			IC80	
Virus ID	Clade*	LN01	10E8		LN01	10E8
6535.3	B	1.031	0.081		6.072	1.32
QH0692.42	B	0.515	0.442		4.028	4.046
SC422661.8	B	0.292	0.445		1.446	2.715
PVO.4	B	0.403	0.747		3.793	>10
TRO.11	B	0.185	0.034		0.855	0.250
AC10.0.29	B	1.894	0.132		7.928	1.534
RHPA4259.7	B	10.306	0.728		>25	5.275
THRO4156.18	B	9.831	0.156		>25	0.884
REJO4541.67	B	0.291	0.203		2.261	1.686
TRJO4551.58	B	8.416	0.475		>25	4.783
WITO4160.33	B	0.224	0.094		1.599	1.226
CAAN5342.A2	B	2.242	1.284		18.329	9.281
WEAU_d15_410_5017	B (T/F)	2.471	>10		>25	>10
1006_11_C3_1601	B (T/F)	0.548	0.172		3.885	2.999
1054_07_TC4_1499	B (T/F)	0.092	0.046		0.551	0.305
1056_10_TA11_1826	B (T/F)	0.060	0.134		0.562	1.028
1012_11_TC21_3257	B (T/F)	0.641	0.244		8.756	4.211
6240_08_TA5_4622	B (T/F)	1.978	0.756		14.746	5.591
6244_13_B5_4576	B (T/F)	0.088	0.086		0.445	0.809
62357_14_D3_4589	B (T/F)	5.043	0.119		20.028	1.060
SC05_8C11_2344	B (T/F)	0.710	0.665		4.945	2.736
Du156.12	C	0.146	0.034		0.473	0.144
Du172.17	C	0.059	0.109		0.285	0.837
Du422.1	C	0.929	0.351		6.282	1.883
ZM197M.PB7	C	0.127	0.148		0.977	0.802
ZM214M.PL15	C	0.840	1.962		7.766	>10
ZM233M.PB6	C	0.673	0.442		3.060	2.393
ZM249M.PL1	C	0.944	1.511		4.646	6.294
ZM53M.PB12	C	11.114	3.985		>25	>10
ZM109F.PB4	C	0.618	0.295		4.857	1.888
ZM135M.PL10a	C	0.710	0.171		3.469	1.186
CAP45.2.00.G3	C	1.878	1.817		10.787	>10
CAP210.2.00.E8	C	>25	0.798		>25	3.892
HIV-001428-2.42	C	24.689	0.809		>25	7.298
HIV-0013095-2.11	C	0.028	0.031		0.136	0.213
HIV-16055-2.3	C	0.174	0.722		1.181	6.495
HIV-16845-2.22	C	0.040	0.068		0.326	0.495
Ce1086_B2	C (T/F)	0.328	1.162		2.851	6.934
Ce0393_C3	C (T/F)	>25	1.179		>25	7.423
Ce1176_A3	C (T/F)	1.092	0.867		3.786	3.923
Ce2010_F5	C (T/F)	11.611	1.844		>25	8.612
Ce0682_E4	C (T/F)	0.626	1.215		7.500	7.709
Ce1172_H1	C (T/F)	0.070	0.722		0.472	4.818
Ce2060_G9	C (T/F)	4.050	3.520		12.431	>10
Ce703010054_2A2	C (T/F)	1.703	2.299		8.142	8.579
BF1266.431a	C (T/F)	>25	2.687		>25	>10
246F C1G	C (T/F)	4.894	2.217		15.945	9.937
249M B10	C (T/F)	2.769	1.271		8.389	9.050
ZM247v1(Rev-)	C (T/F)	0.501	0.506		6.465	6.745
7030102001E5(Rev-)	C (T/F)	4.365	6.707		16.490	>10
1394C9G1(Rev-)	C (T/F)	5.964	0.963		23.305	5.327
Ce704809221_1B3	C (T/F)	3.078	0.058		15.769	0.319
CNE19	BC	1.206	0.666		6.774	6.485
CNE20	BC	0.018	1.246		0.050	6.577
CNE21	BC	0.053	1.900		0.317	>10
CNE17	BC	1.112	1.114		5.618	5.255
CNE30	BC	0.597	1.560		2.081	9.842
CNE52	BC	>25	0.304		>25	3.056
CNE53	BC	0.321	0.326		1.982	1.916
CNE58	BC	0.297	1.289		0.831	8.268

		Titer in TZM-bl cells (ug/ml)				
		IC50			IC80	
Virus ID	Clade*	LN01	10E8		LN01	10E8
MS208.A1	A	>25	0.609		>25	3.717
Q23.17	A	5.513	1.637		19.114	7.040
Q461.e2	A	2.829	2.111		11.579	>10
Q769.d22	A	6.420	1.667		21.297	10.000
Q259.d2.17	A	6.498	4.587		>25	>10
Q842.d12	A	7.934	4.189		>25	>10
0330.v4.c3	A	8.243	2.006		>25	>10
0260.v5.c36	A	>25	>10		>25	>10
191955_A11	A (T/F)	1.668	0.680		8.709	3.532
191084 B7-19	A (T/F)	2.614	5.156		22.185	>10
9004SS_A3_4	A (T/F)	0.409	1.160		2.923	6.761
T257-31	CRF02_AG	21.964	0.880		>25	5.779
928-28	CRF02_AG	0.086	0.384		0.573	1.962
263-8	CRF02_AG	0.355	0.196		1.982	1.728
T250-4	CRF02_AG	2.202	1.346		9.990	8.130
T251-18	CRF02_AG	>25	2.357		>25	>10
T278-50	CRF02_AG	5.102	0.949		21.584	7.460
T255-34	CRF02_AG	0.277	0.778		1.838	4.207
211-9	CRF02_AG	0.583	1.091		2.084	6.508
235-47	CRF02_AG	0.874	0.384		3.713	2.072
620345.c01	CRF01_AE	1.547	0.455		13.141	6.002
CNE8	CRF01_AE	0.028	NT		0.259	NT
C1080.c03	CRF01_AE	0.124	0.061		0.653	0.731
R2184.c04	CRF01_AE	0.890	0.272		7.371	2.455
R1166.c01	CRF01_AE	0.603	0.274		3.454	2.614
R3265.c06	CRF01_AE	3.139	>10		>25	>10
C2101.c01	CRF01_AE	0.739	1.921		6.352	8.260
C3347.c11	CRF01_AE	0.901	0.016		2.939	0.167
C4118.c09	CRF01_AE	2.022	1.622		12.752	9.064
CNE5	CRF01_AE	4.345	1.408		17.424	9.637
BJOX009000.02.4	CRF01_AE	1.212	1.570		6.676	9.954
BJOX015000.11.5	CRF01_AE (T/F)	0.529	0.051		8.603	1.909
BJOX010000.06.2	CRF01_AE (T/F)	0.353	0.341		1.605	5.293
BJOX025000.01.1	CRF01_AE (T/F)	0.186	0.107		1.263	6.325
BJOX028000.10.3	CRF01_AE (T/F)	1.097	0.665		18.970	7.361
X1193_c1	G	0.851	0.497		6.106	4.195
P0402_c2_11	G	3.836	0.172		18.720	3.288
X1254_c3	G	3.005	4.753		12.477	>10
X2088_c9	G	>25	>10		>25	>10
X2131_C1_B5	G	0.229	0.189		1.559	0.974
P1981_C5_3	G	0.115	0.057		0.422	0.285
X1632_S2_B10	G	1.846	1.976		7.366	9.684
3016.v5.c45	D	0.250	0.476		1.103	4.393
A07412M1.vrc12	D	0.326	0.576		2.655	6.054
231965.c01	D	9.503	>10		>25	>10
231966.c02	D	0.948	0.191		5.484	3.130
191821_E6_1	D (T/F)	1.239	1.413		4.886	7.737
3817.v2.c59	CD	14.639	1.181		>25	5.977
6480.v4.c25	CD	6.271	8.079		>25	>10
6952.v1.c20	CD	0.168	0.343		1.392	1.907
6811.v7.c18	CD	5.002	7.535		18.990	>10
89-F1_2_25	CD	3.043	1.112		21.607	10.000
3301.v1.c24	AC	7.543	4.628		24.230	>10
6041.v3.c23	AC	1.720	2.333		11.549	9.766
6540.v4.c1	AC	3.885	1.899		19.733	10.000
6545.v4.c1	AC	2.977	1.778		19.229	8.229
0815.v3.c3	ACD	0.384	0.332		5.546	2.868
3103.v3.c10	ACD	>25	>10		>25	>10

* (T/F): Transmitted / Founder Virus

**MPI: Maximum Percent Inhibition

Table S2 related to Figures 5 and 6. Crystallographic data collection and refinement statistics.

**Data collection**	LN01/MPER-TM1*	LN01/MPER-TM2**	LN01/MPER-TM1+06 PS*
Space group	$P\ 4_3\ 2_1\ 2$	$P\ 2_1\ 2_1\ 2_1$	$P\ 4_3\ 2_1\ 2$
Cell dimensions			
a, b, c (Å)	136.76, 136.76, 146.90	65.68, 144.61, 409.50	137.71, 137.71, 146.72
α, β, γ (°)	90, 90, 90	90, 90, 90	90, 90, 90
Resolution (Å)	47.00-3.20 (3.31-3.20) [1]	49.63-3.90 (4.09-3.90) [1]	48.91-3.10 (3.31-3.10)[1]
Unique reflexions	23542 (2291) [1]	35232 (3519) [1]	26213 (4662) [1]
R_{merge}[2]	0.088 (1.235) [1]	0.144 (0.555) [1]	0.161 (0.968) [1]
R_{pim}[3]	0.029 (0.395) [1]	0.073 (0.279) [1]	0.068 (0.415) [1]
$I / \sigma I$	21.6 (1.7) [1]	7.0 (2.7) [1]	8.8 (1.9) [1]
Completeness (%)	99.69 (99.48) [1]	96.2 (97.7) [1]	99.8 (99.8) [1]
Redundancy	9.6 (9.9) [1]	4.2 (4.3) [1]	7.1 (7.1) [1]
CC (1/2)	0.999 (0.688) [1]	0.995 (0.776) [1]	0.995 (0.671)
**Refinement**			
Resolution (Å)	47.00 - 3.20 (3.51 - 3.20) [1]	48.64 - 3.9 (4.039 - 3.9) [1]	46.21 - 3.1 (3.211 - 3.1) [1]
No. reflections	23526 (2285) [1]	35206 (3518) [1]	26131 (2565) [1]
Reflections used for R_{free}[5]	1208 (117) [1]	1726 (174) [1]	1336 (124) [1]
R_{work}[4] / R_{free}[5]	0.213 / 0.253	0.218/0.260	0.238/0.267
No. atoms			
Protein	7090	14832	7118
Ligand/ion	83	166	92
Water	0	0	0
Wilson B (Å^2)	85	105	77
Average B-factors (Å^2)			
Overall	96	124	69
Protein	96	124	69
Ligand/ion	137	137	81
Water			
R.m.s deviations			
Bond lengths (Å)	0.010	0.007	0.004
Bond angles (°)	1.47	1.17	1.15
Ramachandran Plot (%)			
Favoured	96.22	95.39	95.80
Outliers	0.33	0.85	0.44
PDB ID	6SNC	6SNE	6SND

*1 crystal was used for the structure; **2 crystals was used for the structure; [1] Statistics for the highest resolution shell.

[1] Parentheses refer to outer shell statistics.

[2] $R_{merge} = \Sigma_{hkl} \Sigma_i | I_{hkl,i} - <I_{hkl}> | / \Sigma_{hkl} \Sigma_i I_{hkl,i}$, where $I_{hkl,i}$ is the scaled intensity of the ith measurement of

reflection h, k, l, and $< I_{hkl}>$ is the average intensity for that reflection.

[3] $R_{pim} = \Sigma_{hkl} (1/(n-1))^{1/2} \Sigma_i | I_{hkl,i} - <I_{hkl}> | / \Sigma_{hkl} \Sigma_i I_{hkl,i}$, where n is the number of time a given reflexion has been measured.

[4] $R_{work} = \Sigma_{hkl} | Fo - Fc | / \Sigma_{hkl} | Fo | \times 100$, where Fo and Fc are the observed and calculated structures factors.

[5] R_{free} was calculated as for R_{work}, but on a test set of 5% of the data excluded from refinement.

Table S3 related to Figure 5. Contact residues between gp41 and LN01. The contact distances (cut-off 3.5 Å) were calculated based on the LN01/MPER-TM1+06:0 PS structure.

gp41			LN01			Contact	
Residue name, number , interacting atom			Residue name, number, interacting atom			residue distance (A)	Hydrogen bond
Asn	671	ND2	Tyr	H 58	CD1	3.3	
Phe	673	CD1	Trp	H 100h	CE2	3.4	
Phe	673	CD2	Trp	L 96	CH2	3.5	
Phe	673	CE2	Thr	H 50	CG2	3.4	
Asp	674	OD1	Ser	L 93	C	3.5	
			Thr	L 94	CA	3.3	
			Thr	L 94	N	3.2	***
Asp	674	OD2	Thr	L 94	CB	3.1	
Thr	676	O	Ser	H 100f	CB	3.3	
Thr	676	OG1	Trp	H 100h	NE1	3.0	***
Asn	677	OD1	Tyr	L 32	CE2	3.2	
			Tyr	L 32	CZ	3.5	
Asn	677	ND2	His	L 92	ND1	3.2	***
			His	L 92	O	2.9	***
Trp	680	CD1	Phe	H 100e	O	3.2	
			Ser	H 100f	OG	3.3	
Lys	683	CD	Phe	H 100	O	3.3	
Lys	683	CE	Phe	H 100	O	3.1	
Lys	683	NZ	Phe	H 100	O	2.5	***
			Trp	H 100a	CA	3.4	
			Trp	H 100a	C	3.4	
			Ser	H 100b	O	3	***
Met	687	SD	Trp	H 100a	CZ3	3.4	

H, Ab heavy chain; L, Ab light chain
