# Supplementary material for: Structural Basis for Broad HIV-1 Neutralization by the MPER-Specific Human Broadly Neutralizing Antibody LN01
Source: Cell Host Microbe. 2019 Nov 13;26(5):623–637.e8. doi: 10.1016/j.chom.2019.09.016 (PMC6854463; doi:10.1016/j.chom.2019.09.016)
Supplement: Document S2. Article plus Supplemental Information [file mmc2.pdf]

Article

Cell Host & Microbe

Structural Basis for Broad HIV-1 Neutralization by the MPER-Specific Human Broadly Neutralizing Antibody LN01

Graphical Abstract

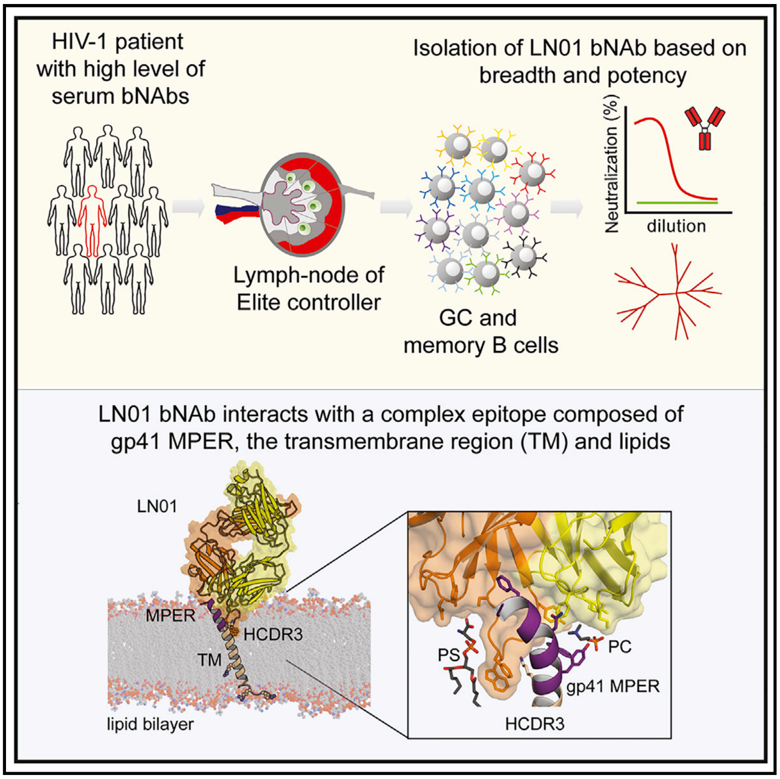

Highlights

- bNAb LN01 neutralizes 92% of a 118-strain virus panel
- LN01 targets the HIV-1 gp41 MPER, the TM region, and lipids
- LN01-complexed MPER forms a continuous helix with TM
- Most LN01 paratope residues interacting with MPER-TM and lipids are germline encoded

Authors

Dora Pinto, Craig Fenwick,
Christophe Caillat, ..., Davide Corti,
Giuseppe Pantaleo,
Winfried Weissenhorn

Correspondence

dcorti@vir.bio (D.C.),
giuseppe.pantaleo@chuv.ch (G.P.),
winfried.weissenhorn@ibs.fr (W.W.)

In Brief

The gp41 membrane-proximal external region (MPER) is a highly conserved region of HIV-1 Env. Pinto et al. characterize the broadly neutralizing anti-MPER mAb LN01, which shows low autoreactivity. LN01 interacts with a complex epitope comprising MPER, the transmembrane region, and lipids, providing insights for vaccine design.

Pinto et al., 2019, Cell Host & Microbe *26*, 623–637
November 13, 2019 © 2019 The Author(s). Published by Elsevier Inc.
https://doi.org/10.1016/j.chom.2019.09.016

CellPress

CellPress

Structural Basis for Broad HIV-1 Neutralization by the MPER-Specific Human Broadly Neutralizing Antibody LN01

Dora Pinto,[1,17,18] Craig Fenwick,[2,17] Christophe Caillat,[3,17] Chiara Silacci,[1] Serafima Guseva,[3] François Dehez,[4,5] Christophe Chipot,[4,5,6] Sonia Barbieri,[1] Andrea Minola,[7] David Jarrossay,[1] Georgia D. Tomaras,[8,13] Xiaoying Shen,[8] Agostino Riva,[9,10] Maciej Tarkowski,[9] Olivier Schwartz,[11,12] Timothée Bruel,[11,12] Jérémy Dufloo,[11,12,13] Michael S. Seaman,[14] David C. Montefiori,[15] Antonio Lanzavecchia,[1] Davide Corti,[7,18,*] Giuseppe Pantaleo,[2,16,*] and Winfried Weissenhorn[3,19,*]

[1]Institute for Research in Biomedicine, Bellinzona 6500, Ticino, Switzerland
[2]Swiss Vaccine Research Institute, Lausanne University Hospital, University of Lausanne, 1011 Lausanne, Switzerland
[3]Institut de Biologie Structurale (IBS), University Grenoble Alpes, CEA, CNRS, 38000 Grenoble, France
[4]LPCT, UMR 7019 Université de Lorraine CNRS, 54500 Vandœuvre-lès-Nancy, France
[5]Laboratoire International Associé CNRS and University of Illinois at Urbana-Champaign, LPCT, UMR 7019 Universiteé de Lorraine CNRS, Vandœuvre-lès-Nancy 54500, France
[6]Department of Physics, University of Illinois at Urbana-Champaign, Urbana, IL 61801, USA
[7]Humabs Biomed SA, Vir Biotechnology, 6500 Bellinzona, Ticino, Switzerland
[8]Duke Human Vaccine Institute, Durham, NC 27710, USA
[9]Department of Biomedical and Clinical Sciences, Luigi Sacco University Hospital, Università di Milano, 20157 Milan, Italy
[10]III Division of Infectious Diseases, ASST Fatebenefratelli-Sacco, 20157 Milan, Italy
[11]Institut Pasteur, Virus & Immunity Unit, CNRS UMR 3569, Paris 75015, France
[12]Vaccine Research Institute, 94000 Créteil, France
[13]Paris Diderot University, Sorbonne Paris Cité, Paris 75013, France
[14]Beth Israel Deaconess Medical Center, Harvard Medical School, Boston, MA 02215, USA
[15]Department of Surgery, Duke University Medical Center, Durham, NC 27710, USA
[16]Service of Immunology and Allergy, Lausanne University Hospital, University of Lausanne, 1011 Lausanne, Switzerland
[17]These authors contributed equally
[18]Present address: Humabs Biomed, a subsidiary of Vir Biotechnology, Bellinzona, Switzerland
[19]Lead Contact
*Correspondence: dcorti@vir.bio (D.C.), giuseppe.pantaleo@chuv.ch (G.P.), winfried.weissenhorn@ibs.fr (W.W.)
https://doi.org/10.1016/j.chom.2019.09.016

SUMMARY

Potent and broadly neutralizing antibodies (bnAbs) are the hallmark of HIV-1 protection by vaccination. The membrane-proximal external region (MPER) of the HIV-1 gp41 fusion protein is targeted by the most broadly reactive HIV-1 neutralizing antibodies. Here, we examine the structural and molecular mechansims of neutralization by anti-MPER bnAb, LN01, which was isolated from lymph-node-derived germinal center B cells of an elite controller and exhibits broad neutralization breadth. LN01 engages both MPER and the transmembrane (TM) region, which together form a continuous helix in complex with LN01. The tilted TM orientation allows LN01 to interact simultaneously with the peptidic component of the MPER epitope and membrane via two specific lipid binding sites of the antibody paratope. Although LN01 carries a high load of somatic mutations, most key residues interacting with the MPER epitope and lipids are germline encoded, lending support for the LN01 epitope as a candidate for lineage-based vaccine development.

INTRODUCTION

The key to HIV-1 vaccine development is the induction of broadly neutralizing antibodies (bnAbs). The currently known classes of bnAbs target six functional regions on the envelope glycoprotein encompassing the V2 apex, the V3 glycan site, the CD4 binding site, the gp120-gp41 interface region, the gp120 silent face, and the membrane proximal external region (MPER) of Env gp41 (Kwong and Mascola, 2018; Sok and Burton, 2018). MPER-specific bnAbs 4E10, 10E8, DH511, and VRC42 target the same helical linear epitope, which precedes the transmembrane (TM) region (Cardoso et al., 2005; Huang et al., 2012; Krebs et al., 2019; Williams et al., 2017; Zwick et al., 2001), and neutralizes more than 90% of multiclade strains (Krebs et al., 2019; Sok and Burton, 2018).

A hallmark of MPER bnAbs are long, heavy-chain CDR3 (HCDR3) loops carrying hydrophobic residues at their tips, whose interaction with membrane is required for neutralization (Alam et al., 2009; Julien et al., 2010; Ofek et al., 2010; Scherer et al., 2010). MPER bnAbs show various degrees of autoreactivity linked to immune tolerance mechanisms (Chen et al., 2013; Doyle-Cooper et al., 2013) that could impair MPER bnAb development. BnAbs 4E10 and VRC42.01 show the most significant non-specific interaction with lipids and membrane (Alam et al., 2007; Krebs et al., 2019), while 10E8 and DH511 lineage bnAbs

This is an open access article under the CC BY license (http://creativecommons.org/licenses/by/4.0/).

A

heavy chain	IGHV	IGHD	IGHV	CDR3-length (amino acid)	VH mutation frequency (aa)
**LN01**	IGHV4-39*07	IGHJ2*01	IGHD3-3*01	20	108/385 (**28%**)

light chain	IGKV	IGKJ	CDR3-length (amino acid)	VL mutation frequency (aa)
**LN01**	IGKV1-39*01	IGKJ1*01	9	87/322 (**27%**)

B

```
heavy chain                  26-35                              52-57
UCA QLQLQESGPGLVKPSETLSLTCTVSGGSISSSSYYWGWIRQPPGKGLEWIGSIYYSGSTYYNP
WT  .V..E.......Q.WG......R...D.V.NDN...A....T..RE.QV..T.....T.....

                                  93-102 (93 94 95 96 97 98 99 100 100a 100b 100c 100d 100e 100f 100g 100h 100i 100j 101 102)
UCA SLKSRVTISVDTSKNQFSLKLSSVTAADTAVYYCVRMPSHGFWSGYFSYWYFDLWGRGTLVTVSS
WT  ..RN.....L.K.V.VV..R.G..S.....Q.............TS............HF.A..W

light chain                  27-31                   50-52
UCA DIQMTQSPSSLSASVGDRVTITCRASQSISSYLNWYQQKPGKAPKLLIYAASSLQSGVPSRFS
WT  .................K..........VTK......F.T.Q.RI....GTYT.L...SP...

                                89-97
UCA GSGSGTDFTLTISSLQPEDFATYYCQQSYSTPWTFGQGTKVEIK
WT  .A...SLY....TNI............AH..........H.AAN
```

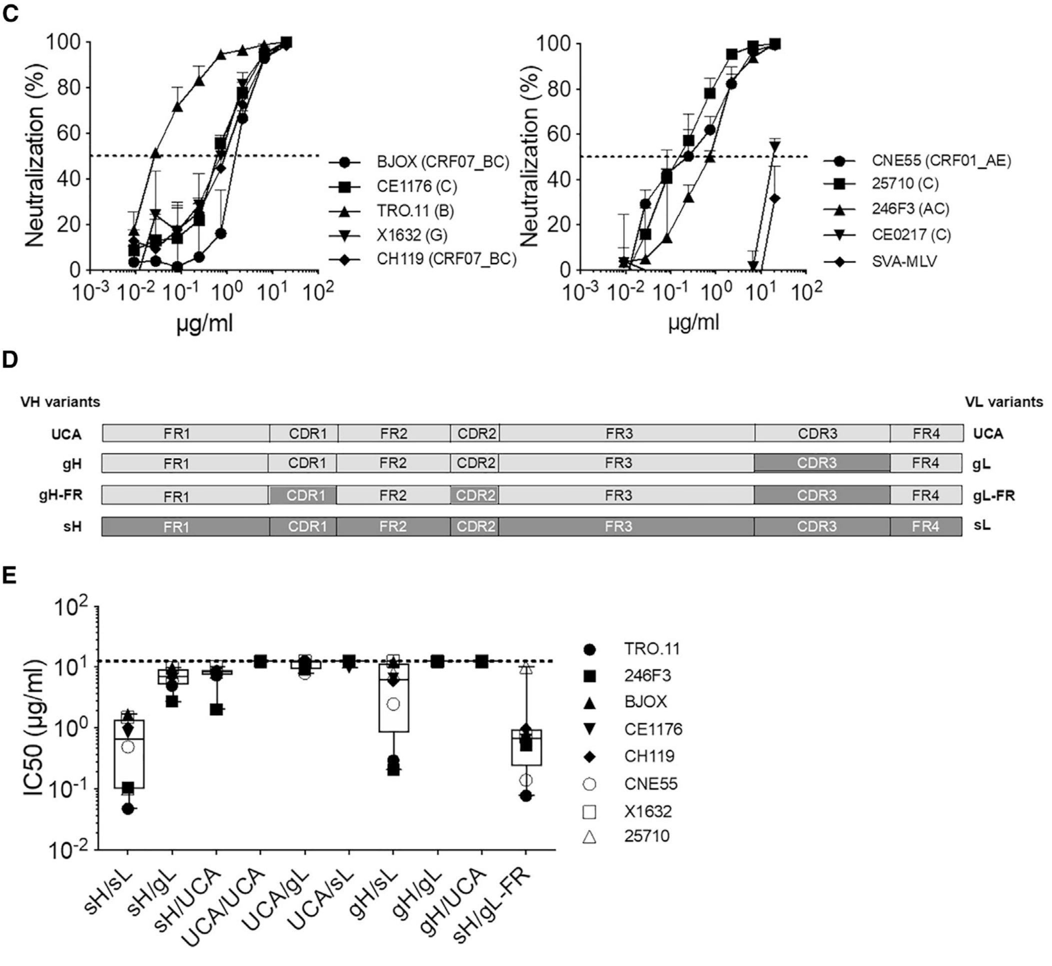

Figure 1. Analysis of LN01 mAb Sequence and Neutralization

(A) Analysis of LN01 sequences showing the inferred germline genes and alleles encoding the variable region of the heavy and light chains, the amino acid length of the CDR3 regions and the mutation frequency of the variable regions of the light and heavy chains (aa, amino acid).

(legend continued on next page)

lack important non-specific membrane binding (Huang et al., 2012; Krebs et al., 2019; Williams et al., 2017), indicating that non-specific membrane autoreactivity is not a prerequisite of bnAbs targeting MPER. However, specific interaction with membrane is important and structures of 4E10 and 10E8 revealed lipid binding of both bnAbs (Irimia et al., 2016; Irimia et al., 2017). Based on these structures, models of the Fab-MPER-membrane interface have been generated and have shown that the MPER epitope helix lies nearly perpendicular to the membrane (Irimia et al., 2016; Irimia et al., 2017; Rujas et al., 2016). Although MPER bnAbs 4E10, 10E8, and DH511 recognize the same epitope, their approach angles vary (Williams et al., 2017). In contrast, VRC42.04 is a close variant of 4E10 that recognizes the same epitope with the same approach angle as 4E10. Interestingly, the latter bnAbs have been independently generated in clade B-and clade CRF016AE-infected patients (Krebs et al., 2019; Zwick et al., 2001).

MPER epitopes are likely only poorly accessible on native Env trimers, and full epitope accessibility requires at least some degree of receptor/co-receptor-binding-induced conformational changes in Env (Chakrabarti et al., 2011; Lee et al., 2016; Rathinakumar et al., 2012). Consequently, MPER bnAbs bind with high affinity to the fusion intermediate conformation of gp41 (Chen et al., 2014; Frey et al., 2008; Lai et al., 2014). This conformation forms during the receptor-binding-induced transitions from native Env (Pancera et al., 2014) to the postfusion conformation (Buzon et al., 2010), thereby facilitating exposure of the linear MPER sequence motif in a membrane context. This transient conformation is suitable for bipartite anti-MPER bnAb binding of the MPER and membrane.

Dependent on the patient cohort investigated, MPER-specific Abs are either present in a large number of patient sera with broad neutralizing activity (Doria-Rose et al., 2017; Huang et al., 2012; Molinos-Albert et al., 2014) or are reported to be rare (Landais et al., 2016; Pietzsch et al., 2010). Importantly, their presence increases the potency and breadth of the sera substantially in comparison to V2 apex bnAbs (Jacob et al., 2015). Furthermore, antibody cocktails including MPER antibodies are more potent (Kong et al., 2015), and *in vitro* the presence of 10E8 in a 4-antibody cocktail reduced significantly the amount of incomplete neutralization (Wagh et al., 2016). Finally, both 4E10 and 10E8 protect animals from SHIV infection by passive immunization (Hessell et al., 2010) (Pegu et al., 2014).

Here, we have isolated a broad and potent anti-MPER neutralizing Ab, LN01, derived from lymph-node germinal center B cells of an elite controller infected with a clade B strain. This antibody uses a heavy-chain germline V gene and thus extends the B cell repertoire for the induction of MPER bnAbs. We have determined the reactivity of the unmutated common ancestor (UCA) and the role of the extensive load of somatic mutations for neutralization. We show that in addition to the MPER epitope, LN01 binding requires part of the TM for interaction. Structural studies have revealed the role of the TM and that of specific lipid-binding pockets. It is noteworthy that MPER forms a continuous helix with the complete gp41 TM region. In synergy with molecular dynamics simulation, we propose a model of LN01 interaction with its monomeric epitope and with the membrane, revealing important implications for gp41 immunogen design targeting the LN01 lineage.

RESULTS

LN01 Isolation and Characterization

Among a cohort of chronically HIV-1-infected patients, naïve to antiretroviral therapy, we identified a patient (SA003) who showed high level of serum bnAbs, as assessed on a panel of 9 HIV-1 pseudoviruses (PVs) from the Global Panel of HIV-1 Env reference strains (Figure S1A). Of note, SA003 donor is an elite controller with viremia <50 HIV-1 RNA copies per mL of plasma (infected with clade B HIV-1). From patient SA003, we isolated lymph node mononuclear cells (LNMC) and sorted IgG memory B cells ($CD19^{+}IgA^{-}IgM^{-}CD27^{+}CD38^{-}$) and IgG germinal center (GC) B cells ($CD19^{+}IgA^{-}IgM^{-}CD27^{+}CD38^{+}$). The two B cell subsets were immortalized with Epstein-Barr virus (EBV) in the presence of anti-B-cell-receptor polyclonal antibodies and cultured for 14 days on a monolayer of mesenchymal stromal cells (MSCs) together with a cocktail of stimuli composed of IL2, IL21, IL6, and the TLR-9 agonist CpG-2006. The supernatants of B cell cultures were screened for their ability to neutralize 2 HIV-1 PVs from the Global Panel, BJOX2000 (clade CFR07) and CE1176 (clade C). One B cell supernatant from the IgG GC B cell library showed a high percentage of neutralization against both PVs tested (>70% for BJOX2000 and >90% for CE1176) (Figure S1B). The VH and VL regions of the monoclonal antibody produced by this B cell clone were sequenced and expressed as recombinant IgG1 monoclonal antibody, hereafter referred to as LN01.

The sequence analysis revealed that LN01 was originally an IgG3 antibody encoding the IGHV4-39 and IGKV1-39 VH and VK germline genes. Two common features of HIV-1 bnAbs were also found in LN01 mAb: high frequency of somatic mutations in the heavy and light chain variable regions compared to the germline sequence (28% and 27%, respectively) and a long HCDR3 loop made of 20 amino acids (Figure 1A). The alignment of LN01 amino acid sequences with the unmutated common ancestor (UCA) sequences showed a high degree of mutations in HCDR1, framework 2 (FR-H2), FR-H4, LCDR1, LCDR2, and FR-L4 (Figure 1B).

(B) Alignment of the amino acid sequences of the variable regions of LN01 wild-type and LN01 UCA. The CDRs regions are highlighted in gray.

(C) LN01 IgG1 activity was tested *in vitro* in neutralization assay using TZM-bl cells. Different concentrations of the antibody were tested against nine pseudoviruses (PVs) of the Global Panel plus a control PV (SVA-MLV). Shown on y axes is the % of neutralization and the standard deviation (SD) calculated on quadruplicates.

(D) Schematic of LN01 unmutated common ancestor (UCA) and variants created for investigation of the neutralization requirements of LN01 germlined variants. Light gray areas represent sequence from UCA; dark gray regions are from the somatic, mature antibody. Wild-type, somatically mutated heavy (sH), or somatically mutated light (sL) chains; gH or gL, germline V-gene revertants of sH or sL in which HCDR3 or LCDR3 are mature; gH-FR or gL-FR, germline V-gene revertants of sH or sL in which HCDRs or LCDRs are mature.

(E) Box-and-whisker plots showing the neutralizing activity of LN01 germline variants against a panel of eight PVs of the Global Panel as measured using a neutralization assay on TZM-bl cells.

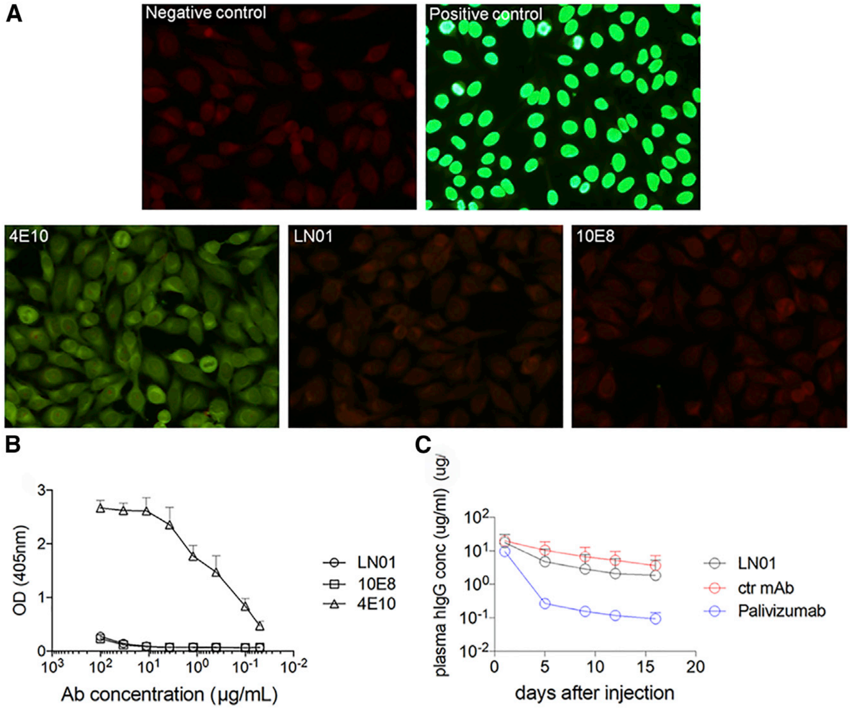

Figure 2. Analysis of LN01 Autoreactivity

(A) Immunofluorescence on Hep-2 cells. BnAbs LN01, 4E10, and 10E8 as well as positive and negative controls provided by the diagnostic kit were tested at 50 μg/mL.

(B) ELISA to measure the binding to the self-antigen cardiolipin. Assay performed according to manufacturer instructions. Shown are OD values of duplicates at 405 nm.

(C) Pharmacokinetic analysis performed in huFcRn transgenic mice (Tg276, Jackson Laboratory). LN01, palivizumab and a control mAb specific for an irrelevant antigen were administered i.v. at 10 mg/kg (n = 5). The concentration of human mAbs in plasma ± standard deviation (SD) was determined at multiple time points using a total human IgG ELISA, as described in STAR Methods.

The neutralizing activity of recombinant LN01 IgG1 mAb was initially tested against a small panel of 9 HIV-1 PVs (and a control PV, SVA-MLV). LN01 was found to neutralize 8 PVs with a median IC_{50} value of 0.57 μg/mL (ranging between 0.03 μg/mL of TRO.11 and 1.61 μg/mL of BJOX2000) (Figure 1C). We also checked the neutralizing activity of LN01 mAb expressed in the IgG3 and Fab formats. Of note, both IgG3 and Fab formats showed the same neutralizing activity as compared with LN01 IgG1 (Figure S2).

To further characterize the LN01 mAb activity we determined whether FcγRI expressed on target cells could augment the potency of Ab-mediated neutralization of HIV-1 PVs, an effect that was previously described to occur with gp41 MPER-specific neutralizing Abs and not with gp120-specific mAbs such as b12 and 2G12 (Perez et al., 2009). Hence, we tested side by side the LN01 IgG1 neutralizing activity on parental TZM-bl cells versus TZM-bl expressing FcγRI in parallel with two anti-MPER bnAbs, 10E8 and 4E10. Differences in neutralization potencies of ≥3-log between parental target cells and FcγRI cells were observed for 10E8 and 4E10, as well as for LN01 (Figure S3). This improved potency may result from a kinetic advantage unique to antibodies whose epitopes are difficult to access or exposed for only a short time, such as the MPER displayed on intermediate conformations of the Env protein during an early stage of fusion. Taken together, these results show that LN01 is a bnAb with high breadth and potency against a multi-clade HIV-1 PVs panel.

Neutralization by LN01 Is Dependent on Somatic Mutations

To investigate the role of somatic mutations on the neutralizing activity of LN01, we generated nine LN01 variants with FR and CDR regions completely reverted to the germline in VH and VK and compared their neutralizing activity to that of the fully mutated LN01 mAb (Figures 1D and 1E). The germlining of all somatic mutations in the heavy chain (UCA VH variant) completely abolished LN01-neutralizing activity against the HIV-1 PVs tested, regardless of which light-chain variants were used (UCA/UCA, UCA/gL, and UCA/sL variants). Similarly, the germlining of all somatic mutations in the light chain (UCA VL variant) also abolished LN01 neutralizing activity, independently of which heavy-chain variants were used (sH/UCA, gH/UCA, UCA/UCA variants).

However, when the mature light chain is paired to the heavy chain completely reverted to the germline except for HCDR3 (gH/sL variant), LN01-neutralizing activity is impaired but not abolished. Of note, when the mature heavy chain is paired to the light chain in which all the FRs are reverted to the germline and the CDRs are mature (sH/gL-FR), the LN01 neutralizing activity is comparable to that of the fully mutated Ab (sH/sL) (Figure 1E). These results show that with respect to the light chain only the somatic mutations in the CDRs (8 mutations in a total of 29 mutations) are required for LN01 activity. Thus, this raises the question whether other somatic mutations can be as well reverted to germline while maintaining the LN01 neutralizing activity.

Analysis of LN01 Autoreactivity

A common feature of several HIV-1 bnAbs, e.g. 2F5, 4E10, and 10E8, is that they cross-react with self-antigens. To evaluate LN01 mAb autoreactivity, we tested its binding to HEp-2 epithelial cells and to cardiolipin. LN01 showed a low binding to HEp-2 cells at the highest concentration tested comparable to the staining with 10E8 but significantly lower than 4E10 (Figure 2A). No binding was observed to cardiolipin (Figure 2B). To further characterize the LN01 bnAb, we performed the pharmacokinetic study in huFcRn transgenic mice. Following intravenous administration, the LN01 serum concentration declined over the 2 weeks of observation with kinetics similar to those of a control mAb specific for an irrelevant antigen and superior to those of the clinical stage mAb palivizumab (Figure 2C). These results show that LN01, based on the preliminary assessment, seems to be

non-autoreactive and to have a favorable pharmacokinetic profile. More in-depth analysis is needed to confirm the safety profile of LN01.

LN01 Neutralization Breadth, Potency, and Effector Function

Since the LN01 mAb showed high breadth and potency against a small panel of HIV-1 PVs, it was then tested against an 118-isolate Env-PVs panel in parallel with bnAb 10E8. LN01 neutralized 92% of the tested PVs compared to 95% for 10E8; the median IC_{50} were 1.1 and 0.8 μg/mL for LN01 and 10E8, respectively. When the IC_{80} values are considered, LN01 and 10E8 neutralized 83% and 85% of the tested PVs with a median of 7.4 and 6.1 μg/mL for LN01 and 10E8, respectively (Figure 3A). Of note, the neutralizing activity of LN01 was not skewed to specific clades, albeit it seems to be less effective on clade A (the SA003 patient was infected by a clade B HIV-1) (Table S1). Thus, LN01 mAb mediates broad and potent neutralization against a large panel of HIV-1 viruses comparable to bnAb 10E8 (Figure 3B).

The targeted killing of HIV-1 infected lymphocytes is an effector function held by some but not all bnAbs. Antibody-dependent cellular cytotoxicity (ADCC) is mediated through the binding of the Fc portion of the antibody to Fc receptors that are expressed on effector cells including natural killer (NK) cells. In complement-dependent cytotoxicity (CDC), antibody binding to the target cell induces cell lysis that is triggered by the binding of C1q subunits to the Fc antibody region. For *in vitro* ADCC evaluations, CEM-NKR-CCR5 CD4 T cells infected with one of four HIV-1 viral strains were cultured with primary NK cells in the presence or absence of different antibodies (Figure 3C). ADCC activities of IgG1 and IgG3 versions of LN01 were compared to that of bnAbs 10E8 and 4E10. BnAbs 3BNC117, 10–1074, PGT128, PGT151 (all IgG1) and the isotype control mG053 were used as controls. One lab-adapted (NLAD8) and three primary strains of HIV-1 (YU2, CH058, and CH077) were tested. In this assay, LN01 IgG1 displays significant ADCC against the four HIV-1 strains tested at levels that are comparable to control bnAbs. LN01 IgG3 showed a reduced response. 10E8 displayed ADCC against 1 out of 4 strains and 4E10 showed no response (Figure 3C). CDC-mediated cell lysis induced through bnAbs was evaluated using Raji cells engineered to stably express cell surface levels of HIV-1 YU2 envelope. Following the incubation of cells with human serum and antibodies, the appearance of dead cells was monitored by flow cytometry with heat-inactivated human serum used as negative control. LN01 IgG1 and IgG3 antibodies both exhibited specific CDC-mediated killing of the envelope positive cells at equivalent levels observed for 10E8, while 4E10 showed substantially reduced CDC activity (Figure 3D).

LN01 Epitope Specificity and Binding

The results described above suggested that LN01 could target the MPER of HIV-1 Env. To address this hypothesis, we used a panel of HIV-2/HIV-1 chimeric PVs containing various segments of the HIV-1 MPER replacing the parental HIV-2/7312A sequences (Tomaras et al., 2011). Importantly, IgG1 LN01 antibody did not neutralize the parental HIV-2 7312A strain. IgG1 LN01 antibody was found to potently neutralize the chimeric virus 7312A.C4 in which six residues from HIV-1 were replaced in the HIV-2 MPER region (**LAS**W**VK**YI**Q** replaced by **ITK**W**LW**YI**K**) but not the chimeric virus 7312A.C6 in which only three residues in the same region were replaced (**LA**SW**V**KYIQ was replaced by **IT**SW**I**KYIQ) (Figure 4A). A similar finding was observed with the chimeric virus 7312A.C1C where the same six mutations of 7312A.C4 were combined with additional seven mutations in the N-terminal region of MPER. These results indicate that residues in the C-terminal region of the gp41 MPER (in particular L679, W680, and K683) are involved in IgG1 LN01 antibody binding and neutralization activity, thus indicating that LN01 is a MPER-specific bnAb.

To better define the specificity of LN01 we used a peptide microarray formed by 1423 15-mer peptides, overlapping by 12 amino acids, that cover the full length of the consensus HIV-1 Env gp160 sequences for clades A, B, C, D, group M, CRF01_AE, and CRF02_AG. LN01 was tested in parallel with a control antibody called 7B2 (that is specific for the immunodominant region of gp41) for binding to the peptide microarray. Of note, IgG1 LN01 antibody did not react with any of the peptides in this library, while 7B2 strongly reacted with 190–195 peptides that spanned the gp41 immunodominant region (Figure S4A). In addition, LN01 did not interact with a 28-amino-acid peptide spanning the entire MPER region (Figure S4B).

To further refine the epitope mapping, we tested LN01 and two other MPER-specific mAbs, 10E8 and 4E10, against a panel of COT6.15 viruses (clade C) encoding MPER mutants (Tomaras et al., 2011). LN01 neutralizing activity was reduced by >8-fold against viruses with the mutants F673A, D674A and D674S (both F673 and D674 are shared in HIV-2), while only marginally reduced against the W672A mutant (Figure 4B). Of note, 10E8 shared with LN01 the reduced activity against the F673A, D674A, and D674S mutants, but its activity was reduced by 2-log against the W672A mutant, indicating that LN01 and 10E8 share a similar interaction with F673 and D674 but are likely to interact differently with W672.

Because LN01 did not interact with a MPER-peptide-containing residues 671–683, longer MPER-containing peptides, MPER-TM1 (residues 671–689 including seven TM residues) and MPER-TM2 (residues 630–711, containing the complete TM region) (Figure S5A) were tested in binding studies. MPER-TM2, solubilized in a buffer containing β-D-octyl glucoside (β-OG), forms trimers (Figure S5B) but does not interact with LN01. However, when solubilized in a buffer containing Fos-Choline-12, MPER-TM2 is monomeric and forms a complex with LN01 Fabs (Figure S5C). Binding kinetics were analyzed both in β-OG and in Fos-Choline-12-containing buffers by surface plasmon resonance (SPR) and compared to 10E8 interaction with MPER-TM1 and MPER-TM2. From this analysis, K_ds were measured to be 160.8 nM for LN01 binding to MPER-TM1 in β-OG and 170.0 nM for interaction with MPER-TM1 in Fos-Choline-12, showing similar binding in both detergents. The interaction with MPER-TM2 is tighter, resulting in a K_d of 13.0 nM, underlining a role for the complete TM region. Binding of 10E8 yielded K_ds of 62.0 and 36.9 nM for the interaction with MPER-TM1 and again a slightly higher affinity of 24.2 nM for the interaction with MPER-TM2 (Figure S5D). We conclude that the LN01 epitope extends from MPER into the TM region.

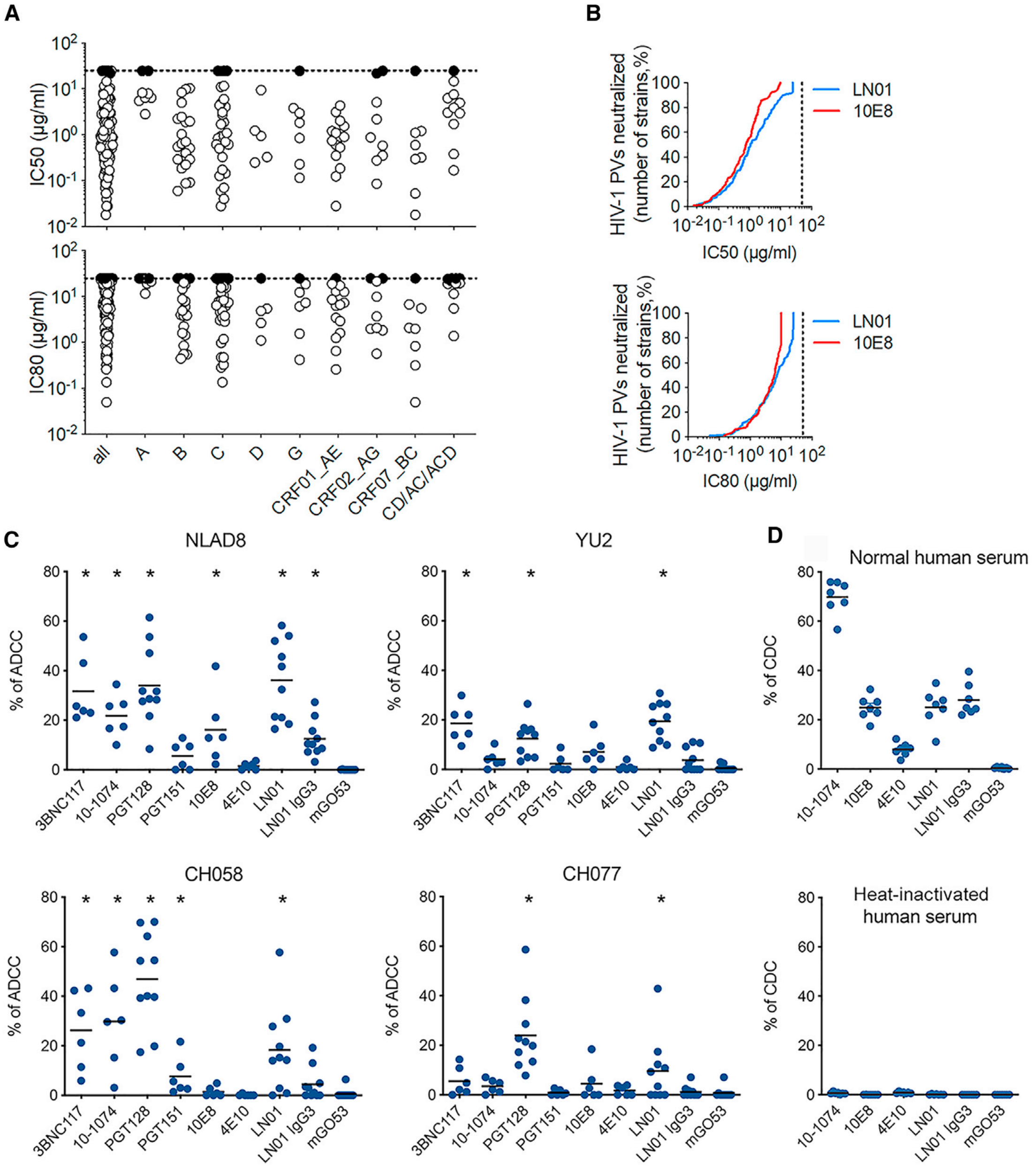

Figure 3. LN01 Neutralization Breadth, Potency, and Effector Function Killing of HIV-1-Infected Lymphocytes

(A) The neutralizing activity of LN01 IgG1 tested against a cross-clade panel of 118 HIV-1 PVs. The IC50 (top panel) and IC80 (bottom panel) expressed in mg/ml were determined in TZM-bl-cell-based micro-neutralization assay as described in STAR Methods.

(B) Neutralization breadth-potency curves for LN01 and 10E8, with breadth shown as percentage of PVs neutralized at each IC50 (top panel) or IC80 (bottom panel) cutoff (25 µg/mL for LN01 and 10 µg/mL for 10E8).

(C) ADCC killing of HIV-1 infected lymphocytes performed with bnAbs at 15 µg/mL on CEM-NKR-CCR5 cells infected with NLAD8, YU2, CH058, or CH077 HIV-1 strains. ADCC was calculated as the disappearance of Gag+ cells with or without antibodies (n = 6–10), with each dot representing an individual donor of primary NK cell. ADCC responses of each tested antibody were compared to that of the isotype control mGO53 in the Wilcoxon test (*p < 0.05).

(D) CDC-mediated cell killing performed with bnAbs at 15 µg/mL incubated with a Raji-YU2 Env cell line in the presence of normal human serum from six individual donors or heat-inactivated human serum. The mGO53 antibody was used as a negative control in (C) and (D).

LN01 Structures in Complex with MPER-TM

Crystal structures of LN01 were determined in complex with MPER-TM1, in complex with MPER-TM1 and phosphatidylserine (06:0 PS), and in complex with MPER-TM2 to resolutions of 3.2 Å, 3.1 Å, and 3.9 Å, respectively (Table S2). Two identical complexes of LN01/MPER-TM1 were present in the asymmetric unit. MPER-TM1 residues 671–689 adopt a slightly bent helical conformation in complex with LN01 (Figure 5A). Main contacts are mediated by polar interactions, D674 to LCDR3 T94, N677 to LCDR3 H92, T676 to HCDR3 W100h, and K683 to the

A

IC50 (ug/ml) in TZM-bl cells		656- 661- 666- 671- 676- 681- 686-
Virus	LN01	
Clade B HIV-1		NEQELLALDKWASLWNWFDITKWLWYIKIFIMIV
HIV-2/7312A	>25	NMYELQKLNSWDVFGNWFDLASWVKYIQYGVYIV
HIV-2/7312A.C1C	**<0.011**	-----LA-D--KNLW----ITK-LW--K------
HIV-2/7312A.C3	>25	-----LA-DK-ASLW------------------
HIV-2/7312A.C4	**<0.011**	------------------ITK-LW--K------
HIV-2/7312A.C6	>25	------------------IT--I----------
HIV-2/7312A.C7	>25	------A-DKWA---------------------
HIV-2/7312A.C8	**<0.011**	------------SLW----ITK-LW--K------

B

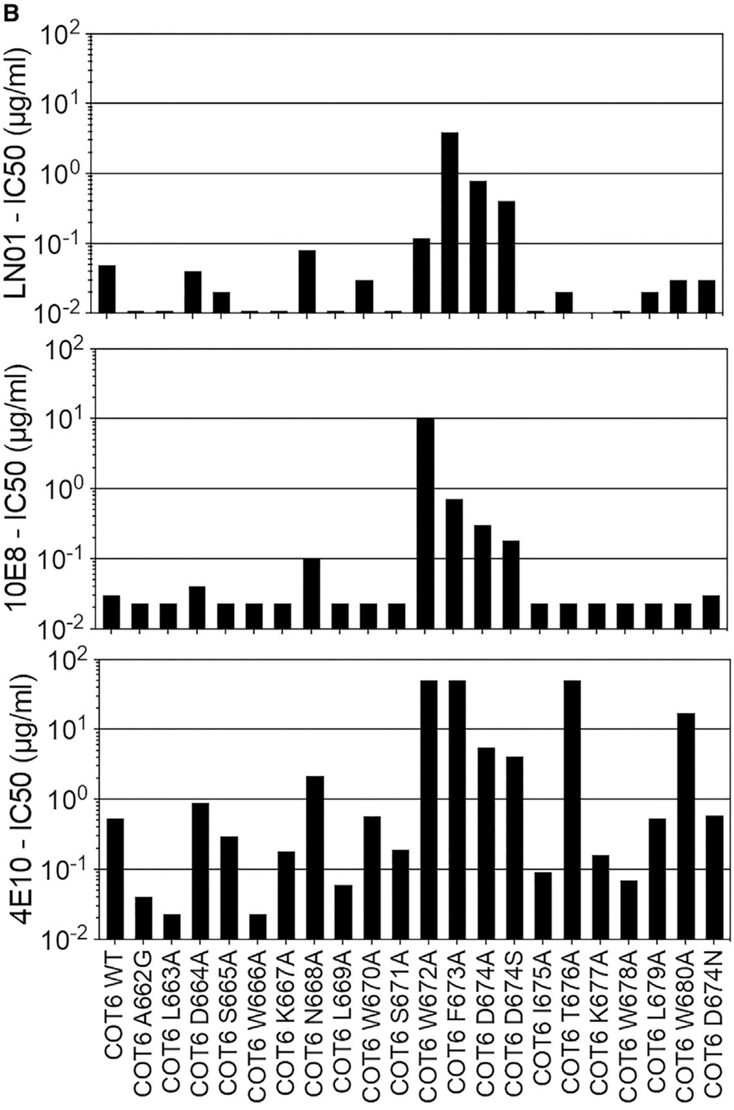

Figure 4. Relevant Residues on HIV-1 PVs for LN01 Binding

(A) HIV-2 chimera containing HIV-1 MPER region and parental HIV-2 were used to determined the binding of recombinant LN01. In the table are shown the IC50 (mg/mL) calculated based on the neutralization assay with TZM-bl cells (middle column), and the different mutations in the sequence carried by MPER of each chimeras (right column).

(B) The activities of LN01, 10E8, and 4E10 mAbs against several MPER mutants of the COT6.15 virus were assessed. Shown are the IC50 values expressed in μg/mL.

carbonyls of HCDR3 F100 and S100b (Figure 5B). Furthermore, gp41 F673 inserts into a hydrophobic pocket composed of HCDR3 W100h, HCDR1 Y35, and LCDR3 W96. In addition, numerous hydrophobic contacts include long-range interactions of HCDR3 residues, F100 and W100a, to gp41 I682, I686, and M687, thereby positioning the tip of HCDR3 close to TM residues (Figure 5B; Table S3). The overall contact interface of the Fab and MPER-TM1 spans 683 $Å^2$. The structure also reveals the presence of a Fos-Choline-12 detergent molecule (Figure S6A); its phosphate group interacts with LCDR1 K31 and the cation of the choline inserts into a cation box (Morita et al., 2016; Roderick et al., 2002) made up of gp41 W680 and Y681 and LCDR1 Y32 and HCDR3 Y100g (Figure 5B). Co-crystallization of MPER-TM1-LN01 with 06:0 phosphatidylserine (PS) (Figure S6B) revealed a network of polar interactions coordinating one PS molecule. HCDR3 S97 and HCDR2 Y52 hydrogen bond to the carbonyl of PS, HCDR1 D32 contacts the amide of PS, the amide of HCDR3 F100 hydrogen bonds to the phosphate group of PS, and HCDR3 W100a to the carbonyl of the acyl chain (Figures 5B and S6B). This interaction network reveals how lipid interaction coordinates the HCDR3 extended conformation, allowing W100a immersion into the lipid bilayer. Notably, most residues coordinating epitope recognition, Fos-Choline-12, and PS interaction in LN01 are present in the LN01 UCA. Exceptions are LCDR1 K31 (Fos-Choline-12, phosphocholine head group), which itself is coordinated by LCDR2 Y52 (Figure 5B), LCDR3 H92 (contacting MPER), HCDR1 D32, and HCDR2 Y52 (contacting PS), as well as LCDR3 H92 (contacting gp41) (Figures 1B and 5B). Furthermore, replacement of UCA HCDR3 G100c by wild-type (WT) HCDR3 T100c likely influences the HCDR3 loop conformation and its capacity to reach the lipid bilayer. Thus, LN01 UCA does not neutralize, mainly because it lacks specific lipid-binding sites.

The neutralization analyses of LN01 variants having different heavy- and light-chain combinations (Figures 1D and 1E) agree with the molecular details of LN01 epitope recognition. In order to define the importance of the interaction with the peptidic epitope, we analyzed the LN01 variants with respect

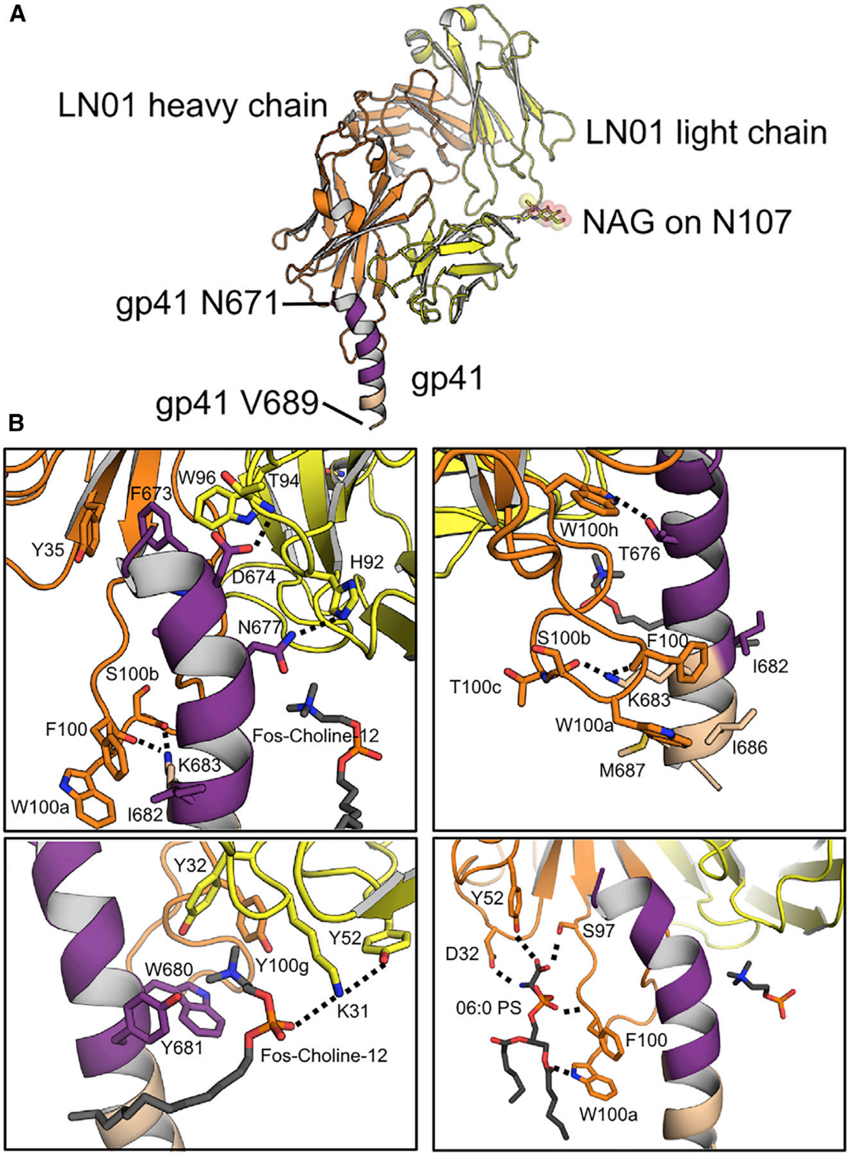

Figure 5. Structure of LN01 in Complex with gp41 MPER-TM1 and with Lipid

(A) Structure of the LN01-MPER-TM1 complex. LN01 is colored in yellow (light chain) and orange (heavy chain) and the N-acetyl-β-d-glucosamine (NAG) on Asn107 of the light chain is shown with spheres. Gp41 is colored in purple (MPER) and beige (TM). The same coloring was used in all figures.

(B) Close up of the interactions with gp41 MPER, Fos-Choline-12, and PS revealing two lipid-binding sites on either side of the MPER helix. Fos-Choline-12, PS, and residues involved in polar contacts and important hydrophobic contacts are indicated and represented in sticks; hydrogen bond interactions are represented as dashed lines. The upper two panels show the interactions of LN01 with gp41 MPER from two different orientations. The lower left panel shows the interaction with Fos-Choline-12, a putative lipid-binding site accommodating phosphatidylcholine, and the lower right panel shows the hydrogen bond network that coordinates 06:0 PS forming a second lipid-binding pocket.

to MPER-TM2 interaction (Figure S7). Using bio-layer interferometry (BLI), WT (sH/sL) LN01 showed a K_D of 2.3 ± 0.01 nM, which is a factor of 5 higher than the K_D determined by SPR (Figure S5D). LN01 sH/gL-FR (Figure 1E) binds the gp41 epitope with a K_D of 7.0 ± 0.03 nM indicating that light-chain framework mutations affect binding slightly while maintaining neutralization potency, except for strain 2570 (Figure 1E). LN01 gH/sL (Figure 1D) binds with a K_D of 6.2 ± 0.02 nM but shows no or lower potency in neutralization (Figure 1E) in agreement with the importance of HCDR1 D32 and HCDR2 Y52 for PS coordination (Figure 5B). LN01 gH/gL and LN01 gH/UCA (Figure 1D) both bind with low micromolar affinity (K_D = 1.4 ± 03 and 4.7 ± 1.7 μM) explaining their lack of neutralization. LN01 sH/gL has a 100-fold lower K_D of 453.3 ± 82 nM and exerts only some low potency neutralization (Figure 1E), which may be explained by the absence of the PS and Fos-Choline12-binding sites as well as the lower affinity due to potential long-range effects of light-chain CDR residues.

LN01 UCA/UCA, UCA/sL, and UCA/gL (Figure 1D) showed no binding, in agreement with no neutralization (Figure 1E), highlighting the importance of HCDR3 interactions with gp41 (Figure 5B). This thus confirms the importance of both high-affinity gp41 peptide epitope interaction and lipid binding for neutralization.

The structure of LN01 in complex with MPER-TM2 containing the complete TM region reveals that MPER forms a continuous helix with the TM comprising residues 684–711 (Figure 6A). The asymmetric unit contained four complexes, two with a continuous straight MPER-TM helix and two with a 90° and 110° kink at the conserved Gly 691 in the middle of the TM region (Figures 6B and S5E). LN01 interactions with MPER are the same in all four complexes and similar to the LN01-MPER-TM1 structures. Molecular dynamics (MD) simulations of the LN01-MPER-TM2 complex placed into a bilayer with a lipid composition analogous to that of the viral envelope (Brügger et al., 2006) revealed a 25° tilted orientation of the TM segment with respect to that of the bilayer (Figure 6C). This tilt orients the LN01 Fab on the membrane such that the HCDR3 dips into the bilayer and positions the lipid-binding sites to interact with lipid head groups (Figure 6D). Furthermore, the tilt angle of the TM allows residue K683 to interact with lipid head groups of one leaflet and R696 to contact head groups of the opposite leaflet; residues R707 and R709 terminating the TM helix interact as well with lipid head groups in the simulation (Figure S6C).

Although some of the LN01-interacting MPER-TM1 residues overlap with the contacts observed for bnAbs 10E8, 4E10, and DH511.1 (DH511.2) (Figure S5E), all four antibodies approach the MPER epitope with different angles. Comparison of the

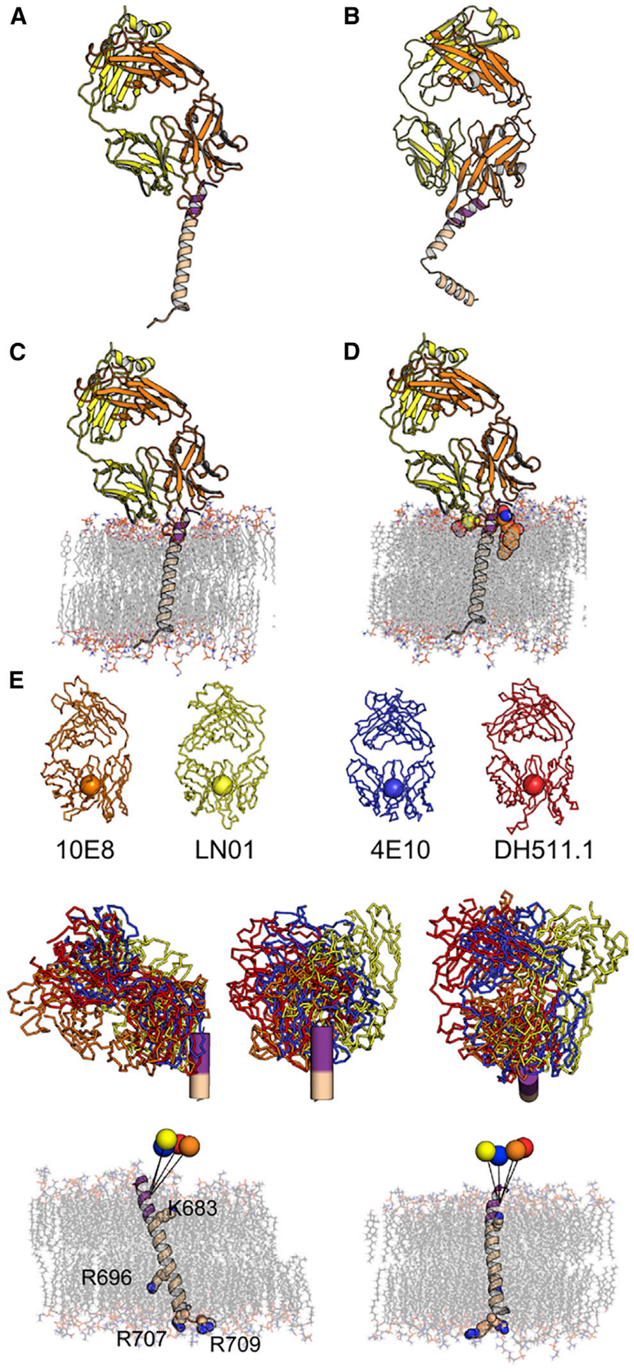

Figure 6. Structure of LN01 in Complex with gp41 MPER-TM2 Reveals a Continuous Helix of MPER and TM

(A) Structure of the LN01-MPER-TM2 complex with the TM in the straight conformation. Color coding is the same as in Figure 5. Gp41 MPER residues 650 to 670 are disordered in the LN01-MPER-TM2 structure.

(B) Structure of the LN01-MPER-TM2 complex with the TM in the bent conformation.

(C) Orientation of the LN01-MPER-TM2 complex in the lipid bilayer based on the MD simulation result (lipids are represented in sticks).

(D) LN01-MPER-TM2 complex with modeled phosphatidylcholine and 06:0 PS (based on the LN01-MPER-TM1 structure) demonstrates that both lipids are well positioned to be part of the bilayer. Phosphatidylcholine and 06:0 PS are shown in spheres.

(E) The angle of approach of MPER bnAbs. The upper panel shows the bnAbs LN01 (yellow), 10E8 (orange), 4E10 (blue), and DH511.1 (red), represented with a sphere at the center of the variable domain for each antibody.

The middle panel shows their orientations upon recognition of the linear helical epitope as determined by Cα superposition of the MPER peptide of the four complexes (two side views and one top view looking down the helical axis of MPER).

The lower panel shows the representation of the angle of approach of the different bnAbs on the MPER-TM domain inserted in the lipid bilayer (lipids and basic residues are shown as sticks). The trajectory between the center of each antibody and gp41 T676 is depicted by a straight line.

approach angles, based on the orientation of the MPER-TM2 in the membrane as determined by MD simulation (Figure 6C), showed that Fabs 10E8, 4E10, and DH511.1 are rotated by 30°, 14°, and 34°, respectively, compared with the LN01 position (Figure 6E). Taken together, the different angles of approach of MPER bnAbs suggest that half of the MPER surface is immunologically silent and not accessible in any of the different conformational states of Env. In order to determine whether TM tilting in the membrane is induced by LN01 binding, we performed MD simulation to position the straight TM alone in the lipid bilayer. Furthermore, we included the bent TM conformation in the simulation to test its potential physiological relevance. Six 0.3-μs MD trajectories were generated, two of them starting from a straight TM helix, and four initiated with a bent TM helix. In the simulations starting from a straight TM helix, TM did not interconvert into a bent form (Figures 7A and 7B). Analysis of the trajectories indicates that the TM is steadily anchored to the lipid bilayer by interactions of K683 with the head groups of one leaflet, and residues R707/709 interacting with head groups of the other leaflet (Figure 7A) as shown in the LN01-MPER-TM2 simulation (Figure S6C). Measuring the tilt of the α helix with respect to the membrane plane as a function of time revealed average tilt angles of 18° ± 5°, slightly smaller than the tilt angle imposed by the LN01 Fab interaction (Figures 5C and S6C). This conformation indicates that the guanidine group of the strictly conserved central TM residue R696 can contact polar head groups (Figures 7A and S6C). In contrast, simulations initiated with the bent TM revealed a propensity to interconvert toward a straight conformation. Out of four independent simulations, only one preserved a bent TM conformation, with a near 90° bending angle. In one simulation, the α helix rapidly retracted to a nearly straight form, while in the remaining two, the TM appears to navigate between the two conformations (Figure 7B). The central R696 can contact polar head groups in the bent conformation as well, and the anchoring residues at the membrane boundaries K683 and R707/709 squeeze the membrane, reducing its diameter locally (Figure 7C). The MD trajectories further suggest that the straight and the bent TM helices in a membrane are metastable states of the free-energy landscape, the straight conformation likely corresponding to a lower free-energy state than the bent form. To verify this hypothesis, the potential of mean force (PMF) underlying the transition between the two conformations was determined in an 11-μs multiple-walker adaptive-biasing-force (MW-ABF) simulation (Comer et al., 2014). The one-dimensional free-energy profile features two minima separated by a 4.5 kcal/mol barrier, the straight and the bent conformations being 3.8 kcal/mol in favor of the former

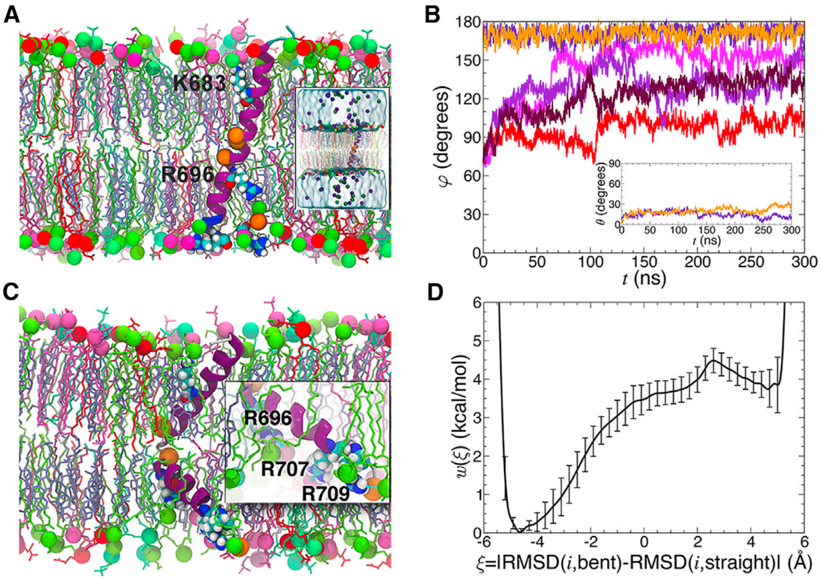

Figure 7. Molecular Dynamics Simulation of the Straight and Bent TM Conformations in a Lipid Bilayer

The lipid bilayer is composed of POPC (turquoise), POPE (yellow-green), POPS (red), SSM (pink), and cholesterol (mauve). The lipid head groups are represented as van der Waals spheres of the corresponding colorcolour. The G residues of the α helix are highlighted as orange spheres.

(A) MD simulation of the straight TM conformation reveals a tilt angle of 17°. The inset shows the complete assay, featuring water and the K^+ (violet) and Cl^- (green) ions.

(B) MD simulation of the bent TM conformation demonstrates that the bent form thins the membrane locally.

(C) Time evolution of the bending angle, φ, formed by the long axes of the α-helical segments spanning residues I675 to M687 and I697 to N706 determined from six independent MD simulations. The inset of (C) depicts the time evolution of the tilt angle, θ, formed by the long axis of gp41 and the normal to the lipid bilayer.

(D) Free-energy profile characterizing the transition between the straight (left minimum) and the bent (right minimum) conformations of gp41 in a membrane. The error bars correspond to the standard deviation measured from the eight walkers of the 11-μs MW-ABF simulation.

(Figure 7D). Thus, starting from a bent α helix, the free-energy barrier to overcome is only 0.7 kcal/mol. These observations indicate that depending on the initial conditions, the bent α helix can rapidly interconvert to a straight α helix. Conversely, at 310K (37°C), the straight α helix is very unlikely to bend to an elbow-shaped α helix, in line with our equilibrium brute-force MD simulations. We conclude that the straight TM region is the lowest energy state, which most likely represents the TM conformation present upon LN01 binding *in vivo*.

DISCUSSION

Here, we report the isolation as well as functional and structural characterization of a bnAb (LN01) isolated from lymph-node GC B cells of an elite controller. LN01, originally isolated as IgG3, uses IGHV4-39 and IGKV1-39 VH, and VK genes and neutralizes 92% of a 118-strain virus panel. We show that LN01 binds to the same gp41 MPER epitope as bnAbs 10E8, 4E10, DH511, and VRC42.04 (Cardoso et al., 2005; Huang et al., 2012; Krebs et al., 2019; Williams et al., 2017). However, LN01 interaction with the MPER epitope requires, in addition to MPER, amino acid residues in adjacent helical turns of the transmembrane region for interaction. Thus, at least one helical turn of the TM is an integral part of the LN01 epitope. Furthermore, the affinity of LN01 is 10 times higher when MPER is fused to the complete TM segment, which may provide a more stable TM structure. In line with a role of the TM for anti-MPER bnAb function, the affinity of 10E8 is 100 times higher when the MPER epitope is linked to part of the TM (Rujas et al., 2016).

The MPER epitope adopts the same helical structure in complex with 10E8, 4E10, DH511, VRC42, and LN01, although the contact residues differ because four of the five bnAbs approach the epitope from different angles. Together, they cover approximately 180° of the cylindrical MPER helix surface, which is inconsistent with a tight trimeric conformation. Indeed, a trimeric NMR model of MPER-TM indicates that the MPER epitope is not accessible in this conformation (Dev et al., 2016). The cryoelectron microscopy (cryo-EM) structure of native Env in complex with PGT151 suggests that glycans N88 and N625 sterically hinder 10E8 binding to native Env trimers. Thus, in order to interact with native Env, 10E8 interaction requires lifting the trimer off the membrane surface, resulting in a conformation similar to the one induced by CD4 splaying MPER-TM apart (Huang et al., 2014; Lee et al., 2016). Although these early conformational states of Env may provide initial binding, the binding affinity of 10E8 or other MPER antibodies to such an early conformational intermediate has not been investigated. The real target of bnAbs targeting MPER is most likely the gp41 fusion intermediate conformation (Frey et al., 2008; Lai et al., 2014), whereas anti-MPER bnAbs prevent refolding into the fusion-active conformation (Weissenhorn et al., 1997). This is also in agreement with the long mean half-life of neutralization (Williams et al., 2017) linked to the prolonged MPER epitope exposure (Shen et al., 2010) and in line with the increased neutralization potencies observed between parental cells and FcγRI cells as reported here.

Most MPER antibodies except VRC42 (Krebs et al., 2019) have been isolated as the IgG3 subclass, whose more flexible hinge region may favour MPER epitope recognition, although no differences in neutralization or CDC activity were observed between LN01 IgG1 and IgG3 subclasses. In contrast, LN01 IgG1 showed a trend toward improved ADCC mediated killing of HIV-1 infected lymphocytes compared to the IgG3. These potential differences between the LN01 antibody subclasses will require further ADCC investigation with a concentration response analysis. Interestingly, using four different strains of HIV-1, LN01 showed a higher ADCC activity than 10E8 and 4E10, the latter being negative in ADCC as reported previously (Bruel et al., 2016).

We further show that LN01 binds lipids similar to 4E10 and 10E8 (Irimia et al., 2016; Irimia et al., 2017), which is likely to be a general feature of MPER bnAbs. The long HCDR3 with hydrophobic residues at its tip, which are essential for neutralization (Alam et al., 2009; Chen et al., 2014; Julien et al., 2010; Lutje Hulsik et al., 2013; Ofek et al., 2010; Scherer et al., 2010), is another common feature of MPER bnAbs. Our MD simulations reveal that LN01 bound to MPER-TM is positioned such that HCDR3 can insert into the membrane, thereby interacting simultaneously with the first turn of the α-helical TM and lipids via at least two lipid-binding pockets. Thus, the membrane is part of a bipartite epitope, which increases the binding affinity (Reichart et al., 2016) and transforms MPER bnAbs into "conformational" Abs (Mouquet et al., 2010). Although membrane interaction is essential for neutralization, LN01 autoreactivity with membranes in the absence of the gp41 epitope is below the detection level similar to that reported for 10E8 (Huang et al., 2012) and VRC42.04 (Krebs et al., 2019) and thus is not as prominent as described for 4E10 (Alam et al., 2007; Haynes et al., 2005). Nevertheless, some autoreactivity such as specific lipid binding plays a role in the maturation of MPER bnAbs, in line with the suggestion that their generation requires the ability to overcome immune tolerance mechanisms (Moody et al., 2016) by engaging antibody lineages induced by host proteins or microbiota (Liao et al., 2011; Trama et al., 2014).

The germline VH and VL gene usage of MPER bnAbs shows similarities and differences. 10E8 and DH511 use the germline V gene IGHV3-15, and 4E10 and VRC42 employ IGHV1-69, while LN01 uses yet another one, IGHV4-39, hence expanding the VH repertoire of naïve B cells for MPER antibody generation. In contrast, the light-chain V gene usage of IGKV1-39 is shared between LN01 and the DH511, while 4E10 and VRC42 use IGKV3-20 and 10E8 IGLV3-19 (Finton et al., 2014; Huang et al., 2012; Krebs et al., 2019; Williams et al., 2017). This indicates that these five anti-MPER bnAbs, derived from four different patients, have found common and divergent solutions to the same problem, suggesting that successful targeting MPER might not be exceptional, in line with the presence of MPER-specific Abs in polyclonal sera (Doria-Rose et al., 2017; Huang et al., 2012). The UCA version of LN01 does not neutralize PVs infection, in agreement with no detectable interaction with the gp41 MPER-TM2 epitope. Testing our different LN01 variants for epitope binding revealed that high-affinity peptide epitope binding, as well as the presence of both proposed lipid-binding sites, are important for LN01 neutralization and potency. Furthermore, many of the LN01 somatic mutations within the framework regions seem to be unimportant for neutralization. However, some long-range structural effects cannot be excluded, in agreement with findings that suggest that HIV-1 bnAbs require framework mutations for either increased flexibility and/or direct antigen contact (Klein et al., 2013). In particular, our structural studies suggest that only five of the CDR somatic mutations are implicated in epitope and lipid binding. Similarly, a longitudinal study on the 4E10-related bnAb VRC42 suggests that it becomes broad with a low mutation rate (Krebs et al., 2019). Thus, further investigations will inform on the minimal requirement of mutations in light of improving LN01 developability properties.

The crystal structure of MPER-TM2 containing the complete TM region reveals a continuous helix of MPER and TM similar to the monomeric MPER-TM NMR structure (Chiliveri et al., 2018). Other structural models propose a hinge or helical bent connecting MPER and TM (Apellániz et al., 2015; Kwon et al., 2018), underlining some conformational flexibility within MPER. MD simulation placed the TM domain alone with an approximate 18° tilt angle in the membrane stabilized by the basic residues at both membrane boundaries. This conformation allows the central, strictly conserved R696 to interact with lipid head groups. Thus, R696 may be strictly conserved in order to position and/or stabilize the tilted TM during the conformational transitions of gp41, leading to membrane fusion (Schibli and Weissenhorn, 2004), which is consistent with the proposal that R696 is important for membrane-fusion efficiency (Long et al., 2011). We further observe a bent conformation of the TM region, which has no role in LN01 interaction. Although this may be a pure Fos-Choline-12 effect, bending occurs at Gly positions that are strictly conserved in HIV-1, HIV-2, and SIV TM sequences (Chen and Chou, 2017). MD simulations confirmed that the TM region can navigate between a straight and bent conformation, both being metastable, although the straight form represents the lower free-energy state. Interestingly, the bent conformation compresses the membrane, and membrane thinning of SNARE TMs was proposed to lower the free energy of stalk formation by favoring the local concentration of highly bent stalk structures (Smirnova et al., 2019) formed at early stages of the fusion process (Chernomordik and Kozlov, 2008). It is, therefore, tempting to speculate that a bent Env TM may have a similar role in membrane fusion.

In summary, the functional and structural data indicate that potential MPER immunogens must be linked to the native Env transmembrane region and incorporated in the membrane as the MPER epitope extends into the TM region, thereby generating the functional bipartite gp41 membrane epitope. Since the TM alone orients itself in the membrane with a tilt angle that is similar to the one induced or stabilized by LN01 interaction, MPER-TM inserted in the membrane may present the optimal immunogen. However, since MPER itself has some degree of conformational flexibility and may as well insert into the membrane when linked to the TM domain (Apellániz et al., 2015; Kwon et al., 2018), our structures indicate that stabilization of the continuous MPER-TM helix by chemical (Bird et al., 2014) or other means could favor immunogenicity and thus B cell stimulation.

STAR★METHODS

Detailed methods are provided in the online version of this paper and include the following:

- KEY RESOURCES TABLE
- LEAD CONTACT AND MATERIALS AVAILABILITY
- EXPERIMENTAL MODEL AND SUBJECT DETAILS
  - Cell Lines
  - HIV-1 Primary Viruses
  - Sample Collection
  - Animals
- METHODS DETAILS
  - B-cell Isolation and Stimulation
  - Sequence Analysis of Antibody cDNA

- ○ Production of Recombinant Antibody and Antibody Variants
- ○ Neutralization Assays
- ○ ADCC Assay
- ○ Complement-dependent Cytotoxicity Assay
- ○ Pharmacokinetic Analysis
- ○ Autoreactivity Assays
- ○ ELISA Assay
- ○ Linear Epitope Mapping
- ○ Gp41 Peptides
- ○ Surface Plasmon Resonance (SPR) Analysis
- ○ Bio-layer Interferometry Binding Analysis
- ○ Crystallization, Data Collection and Structure Determination
- ○ Figure Generation
- ○ Molecular Dynamics (MD) Simulation
- QUANTIFICATION AND STATISTICAL ANALYSIS
- DATA AND CODE AVAILABILITY

SUPPLEMENTAL INFORMATION

Supplemental Information can be found online at https://doi.org/10.1016/j.chom.2019.09.016.

ACKNOWLEDGMENTS

This work was supported by Collaboration for AIDS Vaccine Discovery grants from the Bill and Melinda Gates Foundation (OPP1114725 to G.P. and A.L. and OPP1032144 to D.C.M.). Part of the project (W.W.) has received funding from the European Union's Horizon 2020 research and innovation program under grant agreement no. 681137. W.W. acknowledges the Institute Universitaire de France (IUF) and the platforms of the Grenoble Instruct-ERIC center (ISBG; UMS 3518 CNRS-CEA-UGA-EMBL) within the Grenoble Partnership for Structural Biology (PSB). Platform access was supported by FRISBI (ANR-10-INBS-05-02) and GRAL, a project of the University Grenoble Alpes graduate school (Ecoles Universitaires de Recherche) CBH-EUR-GS (ANR-17-EURE-0003). O.S. acknowledges funding from the Institut Pasteur, the ANRS, the Vaccine Research Institute and the Gilead HIV cure program. We thank the ESRF-EMBL Joint Structural Biology Group for access and support at the ESRF beam lines, J. Marquez (EMBL) from the HTX crystallization facility, J.-B. Reiser and A. Choquet for assistance on the SPR and BLI platforms, C. Mas for assistance on the SEC-MALLS platform, P. Raúl Armoa Ortiz for his contribution to crystallization, H. Mouquet for providing the monoclonal antibodies used in Figures 3C and 3D, and C. Atzori for clinical work.

AUTHOR CONTRIBUTIONS

A.L., D.C., and G.P. conceived and designed the study. W.W. conceived and designed the structural and biophysical work. D.P. designed and performed experiments, and analyzed data. C.F. contributed to conceiving the project and coordinated profiling. C.S. carried out the neutralization studies. C. Caillat produced and characterized complexes by X-ray crystallography and BLI-binding studies. S.G. performed SPR experiments. F.D. and C. Chipot performed molecular dynamics simulation experiments. S.B. carried out antibodies sequencing and cloning. A.M. carried out *in vivo* studies. D.J. carried out cell sorting for B cell isolation. A.R. identified and recruited HIV-infected patients for the study. M.T. processed biological samples from patients. O.S., J.D., and T.B. conceived and performed the ADCC and CDC assays. G.D.T. and X.S. conceived and performed the peptide microarray assay. M.S.S. and D.C.M. performed neutralization assays. D.C., D.P., C.C., and W.W. wrote the manuscript with input from all authors.

DECLARATION OF INTERESTS

The authors declare no competing interests. D.P., A.M., and D.C. are employees of Vir Biotechnology and hold shares in Vir Biotechnology. LN01 is covered by patent application no. WO 2017/093985A1. Inventors: G.P. and A.L.

Received: July 1, 2019
Revised: August 29, 2019
Accepted: September 27, 2019
Published: October 22, 2019

REFERENCES

Adams, P.D., Afonine, P.V., Bunkóczi, G., Chen, V.B., Davis, I.W., Echols, N., Headd, J.J., Hung, L.W., Kapral, G.J., Grosse-Kunstleve, R.W., et al. (2010). PHENIX: a comprehensive Python-based system for macromolecular structure solution. Acta Crystallogr. D Biol. Crystallogr. *66*, 213–221.

Alam, S.M., McAdams, M., Boren, D., Rak, M., Scearce, R.M., Gao, F., Camacho, Z.T., Gewirth, D., Kelsoe, G., Chen, P., et al. (2007). The role of antibody polyspecificity and lipid reactivity in binding of broadly neutralizing anti-HIV-1 envelope human monoclonal antibodies 2F5 and 4E10 to glycoprotein 41 membrane proximal envelope epitopes. J. Immunol. *178*, 4424–4435.

Alam, S.M., Morelli, M., Dennison, S.M., Liao, H.X., Zhang, R., Xia, S.M., Rits-Volloch, S., Sun, L., Harrison, S.C., Haynes, B.F., et al. (2009). Role of HIV membrane in neutralization by two broadly neutralizing antibodies. Proc. Natl. Acad. Sci. USA *106*, 20234–20239.

Andersen, H.C. (1983). Rattle: a "velocity" version of the shake algorithm for molecular dynamics calculations. J. Comput. Phys. *52*, 24–34.

Apellániz, B., Rujas, E., Serrano, S., Morante, K., Tsumoto, K., Caaveiro, J.M., Jiménez, M.Á., and Nieva, J.L. (2015). The atomic structure of the HIV-1 gp41 transmembrane domain and its connection to the immunogenic membrane-proximal external region. J. Biol. Chem. *290*, 12999–13015.

Bird, G.H., Irimia, A., Ofek, G., Kwong, P.D., Wilson, I.A., and Walensky, L.D. (2014). Stapled HIV-1 peptides recapitulate antigenic structures and engage broadly neutralizing antibodies. Nat. Struct. Mol. Biol. *21*, 1058–1067.

Bruel, T., Guivel-Benhassine, F., Amraoui, S., Malbec, M., Richard, L., Bourdic, K., Donahue, D.A., Lorin, V., Casartelli, N., Noël, N., et al. (2016). Elimination of HIV-1-infected cells by broadly neutralizing antibodies. Nat. Commun. *7*, 10844.

Brügger, B., Glass, B., Haberkant, P., Leibrecht, I., Wieland, F.T., and Kräusslich, H.G. (2006). The HIV lipidome: a raft with an unusual composition. Proc. Natl. Acad. Sci. USA *103*, 2641–2646.

Buzon, V., Natrajan, G., Schibli, D., Campelo, F., Kozlov, M.M., and Weissenhorn, W. (2010). Crystal structure of HIV-1 gp41 including both fusion peptide and membrane proximal external regions. PLoS Pathog. *6*, e1000880.

Cardoso, R.M., Zwick, M.B., Stanfield, R.L., Kunert, R., Binley, J.M., Katinger, H., Burton, D.R., and Wilson, I.A. (2005). Broadly neutralizing anti-HIV antibody 4E10 recognizes a helical conformation of a highly conserved fusion-associated motif in gp41. Immunity *22*, 163–173.

Casartelli, N., Sourisseau, M., Feldmann, J., Guivel-Benhassine, F., Mallet, A., Marcelin, A.G., Guatelli, J., and Schwartz, O. (2010). Tetherin restricts productive HIV-1 cell-to-cell transmission. PLoS Pathog. *6*, e1000955.

Chakrabarti, B.K., Walker, L.M., Guenaga, J.F., Ghobbeh, A., Poignard, P., Burton, D.R., and Wyatt, R.T. (2011). Direct antibody access to the HIV-1 membrane-proximal external region positively correlates with neutralization sensitivity. J. Virol. *85*, 8217–8226.

Chen, B., and Chou, J.J. (2017). Structure of the transmembrane domain of HIV-1 envelope glycoprotein. FEBS J. *284*, 1171–1177.

Chen, J., Frey, G., Peng, H., Rits-Volloch, S., Garrity, J., Seaman, M.S., and Chen, B. (2014). Mechanism of HIV-1 neutralization by antibodies targeting a membrane-proximal region of gp41. J. Virol. *88*, 1249–1258.

Chen, Y., Zhang, J., Hwang, K.K., Bouton-Verville, H., Xia, S.M., Newman, A., Ouyang, Y.B., Haynes, B.F., and Verkoczy, L. (2013). Common tolerance mechanisms, but distinct cross-reactivities associated with gp41 and lipids, limit production of HIV-1 broad neutralizing antibodies 2F5 and 4E10. J. Immunol. *191*, 1260–1275.

Chernomordik, L.V., and Kozlov, M.M. (2008). Mechanics of membrane fusion. Nat. Struct. Mol. Biol. *15*, 675–683.

Chiliveri, S.C., Louis, J.M., Ghirlando, R., Baber, J.L., and Bax, A. (2018). Tilted, uninterrupted, monomeric HIV-1 gp41 transmembrane helix from residual dipolar couplings. J. Am. Chem. Soc. *140*, 34–37.

Comer, J., Gumbart, J.C., Hénin, J., Lelièvre, T., Pohorille, A., and Chipot, C. (2015). The adaptive biasing force method: everything you always wanted to know but were afraid to ask. J. Phys. Chem. *119*, 1129–1151.

Comer, J., Phillips, J.C., Schulten, K., and Chipot, C. (2014). Multiple-replica strategies for free-energy calculations in NAMD: multiple-walker adaptive biasing force and Walker selection rules. J. Chem. Theor. Comput. *10*, 5276–5285.

Darden, T., York, D., and Pedersen, L. (1993). Particle mesh Ewald: an N•log (N) method for Ewald sums in large systems. J. Chem. Phys. *98*, 10089–10092.

Dev, J., Park, D., Fu, Q., Chen, J., Ha, H.J., Ghantous, F., Herrmann, T., Chang, W., Liu, Z., Frey, G., et al. (2016). Structural basis for membrane anchoring of HIV-1 envelope spike. Science *353*, 172–175.

Doria-Rose, N.A., Altae-Tran, H.R., Roark, R.S., Schmidt, S.D., Sutton, M.S., Louder, M.K., Chuang, G.Y., Bailer, R.T., Cortez, V., Kong, R., et al. (2017). Mapping polyclonal HIV-1 antibody responses via next-generation neutralization fingerprinting. PLoS Pathog. *13*, e1006148.

Doyle-Cooper, C., Hudson, K.E., Cooper, A.B., Ota, T., Skog, P., Dawson, P.E., Zwick, M.B., Schief, W.R., Burton, D.R., and Nemazee, D. (2013). Immune tolerance negatively regulates B cells in knock-in mice expressing broadly neutralizing HIV antibody 4E10. J. Immunol. *191*, 3186–3191.

Emsley, P., Lohkamp, B., Scott, W.G., and Cowtan, K. (2010). Features and development of coot. Acta Crystallogr. D Biol. Crystallogr. *66*, 486–501.

Falkowska, E., Le, K.M., Ramos, A., Doores, K.J., Lee, J.H., Blattner, C., Ramirez, A., Derking, R., van Gils, M.J., Liang, C.H., et al. (2014). Broadly neutralizing HIV antibodies define a glycan-dependent epitope on the prefusion conformation of gp41 on cleaved envelope trimers. Immunity *40*, 657–668.

Feller, S.E., Zhang, Y.H., Pastor, R.W., and Brooks, B.R. (1995). Constant pressure molecular dynamics simulations – the Langevin piston method. J. Chem. Phys. *103*, 4613–4621.

Finton, K.A., Friend, D., Jaffe, J., Gewe, M., Holmes, M.A., Larman, H.B., Stuart, A., Larimore, K., Greenberg, P.D., Elledge, S.J., et al. (2014). Ontogeny of recognition specificity and functionality for the broadly neutralizing anti-HIV antibody 4E10. PLoS Pathog. *10*, e1004403.

Frey, G., Peng, H., Rits-Volloch, S., Morelli, M., Cheng, Y., and Chen, B. (2008). A fusion-intermediate state of HIV-1 gp41 targeted by broadly neutralizing antibodies. Proc. Natl. Acad. Sci. USA *105*, 3739–3744.

Gottardo, R., Bailer, R.T., Korber, B.T., Gnanakaran, S., Phillips, J., Shen, X., Tomaras, G.D., Turk, E., Imholte, G., Eckler, L., et al. (2013). Plasma IgG to linear epitopes in the V2 and V3 regions of HIV-1 gp120 correlate with a reduced risk of infection in the RV144 vaccine efficacy trial. PLoS One *8*, e75665.

Haynes, B.F., Fleming, J., St Clair, E.W., Katinger, H., Stiegler, G., Kunert, R., Robinson, J., Scearce, R.M., Plonk, K., Staats, H.F., et al. (2005). Cardiolipin polyspecific autoreactivity in two broadly neutralizing HIV-1 antibodies. Science *308*, 1906–1908.

Hessell, A.J., Rakasz, E.G., Tehrani, D.M., Huber, M., Weisgrau, K.L., Landucci, G., Forthal, D.N., Koff, W.C., Poignard, P., Watkins, D.I., et al. (2010). Broadly neutralizing monoclonal antibodies 2F5 and 4E10 directed against the human immunodeficiency virus type 1 gp41 membrane-proximal external region protect against mucosal challenge by simian-human immunodeficiency virus SHIVBa-L. J. Virol. *84*, 1302–1313.

Huang, J., Kang, B.H., Pancera, M., Lee, J.H., Tong, T., Feng, Y., Imamichi, H., Georgiev, I.S., Chuang, G.Y., Druz, A., et al. (2014). Broad and potent HIV-1 neutralization by a human antibody that binds the gp41-gp120 interface. Nature *515*, 138–142.

Huang, J., Ofek, G., Laub, L., Louder, M.K., Doria-Rose, N.A., Longo, N.S., Imamichi, H., Bailer, R.T., Chakrabarti, B., Sharma, S.K., et al. (2012). Broad and potent neutralization of HIV-1 by a gp41-specific human antibody. Nature *491*, 406–412.

Huang, Y., Yu, J., Lanzi, A., Yao, X., Andrews, C.D., Tsai, L., Gajjar, M.R., Sun, M., Seaman, M.S., Padte, N.N., et al. (2016). Engineered bispecific antibodies with exquisite HIV-1-neutralizing activity. Cell *165*, 1621–1631.

Humphrey, W., Dalke, A., and Schulten, K. (1996). VMD: visual molecular dynamics. J. Mol. Graphics *14* (33–38), 27–38.

Irimia, A., Sarkar, A., Stanfield, R.L., and Wilson, I.A. (2016). Crystallographic identification of lipid as an integral component of the epitope of HIV broadly neutralizing antibody 4E10. Immunity *44*, 21–31.

Irimia, A., Serra, A.M., Sarkar, A., Jacak, R., Kalyuzhniy, O., Sok, D., Saye-Francisco, K.L., Schiffner, T., Tingle, R., Kubitz, M., et al. (2017). Lipid interactions and angle of approach to the HIV-1 viral membrane of broadly neutralizing antibody 10E8: insights for vaccine and therapeutic design. PLoS Pathog. *13*, e1006212.

Jacob, R.A., Moyo, T., Schomaker, M., Abrahams, F., Grau Pujol, B., and Dorfman, J.R. (2015). Anti-V3/glycan and anti-MPER neutralizing antibodies, but not anti-V2/glycan site antibodies, are strongly associated with greater anti-HIV-1 neutralization breadth and potency. J. Virol *89*, 5264–5275.

Jo, S., Kim, T., Iyer, V.G., and Im, W. (2008). CHARMM-GUI: a web-based graphical user interface for CHARMM. J. Comput. Chem. *29*, 1859–1865.

Jorgensen, W.L., Chandrasekhar, J., Madura, J.D., Impey, R.W., and Klein, M.L. (1983). Comparison of simple potential functions for simulating liquid water. J. Chem. Phys. *79*, 926–935.

Julien, J.P., Huarte, N., Maeso, R., Taneva, S.G., Cunningham, A., Nieva, J.L., and Pai, E.F. (2010). Ablation of the complementarity-determining region H3 apex of the anti-HIV-1 broadly neutralizing antibody 2F5 abrogates neutralizing capacity without affecting core epitope binding. J. Virol. *84*, 4136–4147.

Kabsch, W. (2010). Integration, scaling, space-group assignment and post-refinement. Acta Crystallogr. D Biol. Crystallogr. *66*, 133–144.

Klauda, J.B., Venable, R.M., Freites, J.A., O'Connor, J.W., Tobias, D.J., Mondragon-Ramirez, C., Vorobyov, I., MacKerell, A.D., Jr., and Pastor, R.W. (2010). Update of the CHARMM all-atom additive force field for lipids: validation on six lipid types. J. Phys. Chem. B *114*, 7830–7843.

Klein, F., Diskin, R., Scheid, J.F., Gaebler, C., Mouquet, H., Georgiev, I.S., Pancera, M., Zhou, T., Incesu, R.B., Fu, B.Z., et al. (2013). Somatic mutations of the immunoglobulin framework are generally required for broad and potent HIV-1 neutralization. Cell *153*, 126–138.

Kong, R., Louder, M.K., Wagh, K., Bailer, R.T., deCamp, A., Greene, K., Gao, H., Taft, J.D., Gazumyan, A., Liu, C., et al. (2015). Improving neutralization potency and breadth by combining broadly reactive HIV-1 antibodies targeting major neutralization epitopes. J. Virol. *89*, 2659–2671.

Krebs, S.J., Kwon, Y.D., Schramm, C.A., Law, W.H., Donofrio, G., Zhou, K.H., Gift, S., Dussupt, V., Georgiev, I.S., Schätzle, S., et al. (2019). Longitudinal analysis reveals early development of three MPER-directed neutralizing antibody lineages from an HIV-1-infected individual. Immunity *50*, 677–691.e13.

Kwon, B., Lee, M., Waring, A.J., and Hong, M. (2018). Oligomeric structure and three-dimensional fold of the HIV gp41 membrane-proximal external region and transmembrane domain in phospholipid bilayers. J. Am. Chem. Soc. *140*, 8246–8259.

Kwong, P.D., and Mascola, J.R. (2018). HIV-1 vaccines based on antibody identification, B cell ontogeny, and epitope structure. Immunity *48*, 855–871.

Lai, R.P., Hock, M., Radzimanowski, J., Tonks, P., Hulsik, D.L., Effantin, G., Seilly, D.J., Dreja, H., Kliche, A., Wagner, R., et al. (2014). A fusion intermediate gp41 immunogen elicits neutralizing antibodies to HIV-1. J. Biol. Chem. *289*, 29912–29926.

Landais, E., Huang, X., Havenar-Daughton, C., Murrell, B., Price, M.A., Wickramasinghe, L., Ramos, A., Bian, C.B., Simek, M., Allen, S., et al. (2016). Broadly neutralizing antibody responses in a large longitudinal sub-Saharan HIV primary infection cohort. PLoS Pathog. *12*, e1005369.

Lee, J.H., Ozorowski, G., and Ward, A.B. (2016). Cryo-EM structure of a native, fully glycosylated, cleaved HIV-1 envelope trimer. Science *351*, 1043–1048.

Liao, H.X., Chen, X., Munshaw, S., Zhang, R., Marshall, D.J., Vandergrift, N., Whitesides, J.F., Lu, X., Yu, J.S., Hwang, K.K., et al. (2011). Initial antibodies binding to HIV-1 gp41 in acutely infected subjects are polyreactive and highly mutated. J. Exp. Med. *208*, 2237–2249.

Long, Y., Meng, F., Kondo, N., Iwamoto, A., and Matsuda, Z. (2011). Conserved arginine residue in the membrane-spanning domain of HIV-1 gp41 is required for efficient membrane fusion. Protein Cell *2*, 369–376.

Lutje Hulsik, D., Liu, Y.Y., Strokappe, N.M., Battella, S., El Khattabi, M., McCoy, L.E., Sabin, C., Hinz, A., Hock, M., Macheboeuf, P., et al. (2013). A gp41 MPER-specific llama VHH requires a hydrophobic CDR3 for neutralization but not for antigen recognition. PLoS Pathog. *9*, e1003202.

MacKerell, A.D., Bashford, D., Bellott, M., Dunbrack, R.L., Evanseck, J.D., Field, M.J., Fischer, S., Gao, J., Guo, H., Ha, S., et al. (1998). All-atom empirical potential for molecular modeling and dynamics studies of proteins. J. Phys. Chem. B *102*, 3586–3616.

McCoy, A.J., Grosse-Kunstleve, R.W., Adams, P.D., Winn, M.D., Storoni, L.C., and Read, R.J. (2007). Phaser crystallographic software. J. Appl. Crystallogr. *40*, 658–674.

Miyamoto, S., and Kollman, P.A. (1992). SETTLE: an analytical version of the SHAKE and RATTLE algorithms for rigid water models. J. Comput. Chem. *13*, 952–962.

Molinos-Albert, L.M., Carrillo, J., Curriu, M., Rodriguez de la Concepción, M.L., Marfil, S., García, E., Clotet, B., and Blanco, J. (2014). Anti-MPER antibodies with heterogeneous neutralization capacity are detectable in most untreated HIV-1 infected individuals. Retrovirology *11*, 44.

Montefiori, D.C. (2009). Measuring HIV neutralization in a luciferase reporter gene assay. Methods Mol. Biol. *485*, 395–405.

Moody, M.A., Pedroza-Pacheco, I., Vandergrift, N.A., Chui, C., Lloyd, K.E., Parks, R., Soderberg, K.A., Ogbe, A.T., Cohen, M.S., Liao, H.X., et al. (2016). Immune perturbations in HIV-1-infected individuals who make broadly neutralizing antibodies. Sci. Immunol. *1*, aag0851.

Morin, A., Eisenbraun, B., Key, J., Sanschagrin, P.C., Timony, M.A., Ottaviano, M., and Sliz, P. (2013). Collaboration gets the most out of software. Elife *2*, e01456.

Morita, J., Kano, K., Kato, K., Takita, H., Sakagami, H., Yamamoto, Y., Mihara, E., Ueda, H., Sato, T., Tokuyama, H., et al. (2016). Structure and biological function of ENPP6, a choline-specific glycerophosphodiester-phosphodiesterase. Sci. Rep. *6*, 20995.

Mouquet, H., Scharf, L., Euler, Z., Liu, Y., Eden, C., Scheid, J.F., Halper-Stromberg, A., Gnanapragasam, P.N., Spencer, D.I., Seaman, M.S., et al. (2012). Complex-type N-glycan recognition by potent broadly neutralizing HIV antibodies. Proc. Natl. Acad. Sci. USA *109*, E3268–E3277.

Mouquet, H., Scheid, J.F., Zoller, M.J., Krogsgaard, M., Ott, R.G., Shukair, S., Artyomov, M.N., Pietzsch, J., Connors, M., Pereyra, F., et al. (2010). Polyreactivity increases the apparent affinity of anti-HIV antibodies by heteroligation. Nature *467*, 591–595.

Murshudov, G.N., Vagin, A.A., and Dodson, E.J. (1997). Refinement of macromolecular structures by the maximum-likelihood method. Acta Crystallogr. D. Biol. Crystallogr. *53*, 240–255.

Ofek, G., McKee, K., Yang, Y., Yang, Z.Y., Skinner, J., Guenaga, F.J., Wyatt, R., Zwick, M.B., Nabel, G.J., Mascola, J.R., et al. (2010). Relationship between antibody 2F5 neutralization of HIV-1 and hydrophobicity of its heavy chain third complementarity-determining region. J. Virol. *84*, 2955–2962.

Pancera, M., Zhou, T., Druz, A., Georgiev, I.S., Soto, C., Gorman, J., Huang, J., Acharya, P., Chuang, G.Y., Ofek, G., et al. (2014). Structure and immune recognition of trimeric pre-fusion HIV-1 Env. Nature *514*, 455–461.

Pegu, A., Yang, Z.Y., Boyington, J.C., Wu, L., Ko, S.Y., Schmidt, S.D., McKee, K., Kong, W.P., Shi, W., Chen, X., et al. (2014). Neutralizing antibodies to HIV-1 envelope protect more effectively in vivo than those to the CD4 receptor. Sci. Transl. Med *6*, 243ra88.

Perez, L.G., Costa, M.R., Todd, C.A., Haynes, B.F., and Montefiori, D.C. (2009). Utilization of immunoglobulin G Fc receptors by human immunodeficiency virus type 1: a specific role for antibodies against the membrane-proximal external region of gp41. J. Virol. *83*, 7397–7410.

Phillips, J.C., Braun, R., Wang, W., Gumbart, J., Tajkhorshid, E., Villa, E., Chipot, C., Skeel, R.D., Kalé, L., and Schulten, K. (2005). Scalable molecular dynamics with NAMD. J. Comput. Chem. *26*, 1781–1802.

Pietzsch, J., Scheid, J.F., Mouquet, H., Seaman, M.S., Broder, C.C., and Nussenzweig, M.C. (2010). Anti-gp41 antibodies cloned from HIV-infected patients with broadly neutralizing serologic activity. J. Virol. *84*, 5032–5042.

Rathinakumar, R., Dutta, M., Zhu, P., Johnson, W.E., and Roux, K.H. (2012). Binding of anti-membrane-proximal gp41 monoclonal antibodies to CD4-liganded and -unliganded human immunodeficiency virus type 1 and simian immunodeficiency virus virions. J. Virol. *86*, 1820–1831.

Reichart, T.M., Baksh, M.M., Rhee, J.K., Fiedler, J.D., Sligar, S.G., Finn, M.G., Zwick, M.B., and Dawson, P.E. (2016). Trimerization of the HIV transmembrane domain in lipid bilayers modulates broadly neutralizing antibody binding. Angew. Chem. Int. Ed. Engl. *55*, 2688–2692.

Roderick, S.L., Chan, W.W., Agate, D.S., Olsen, L.R., Vetting, M.W., Rajashankar, K.R., and Cohen, D.E. (2002). Structure of human phosphatidylcholine transfer protein in complex with its ligand. Nat. Struct. Biol. *9*, 507–511.

Rujas, E., Caaveiro, J.M., Partida-Hanon, A., Gulzar, N., Morante, K., Apellániz, B., García-Porras, M., Bruix, M., Tsumoto, K., Scott, J.K., et al. (2016). Structural basis for broad neutralization of HIV-1 through the molecular recognition of 10E8 helical epitope at the membrane interface. Sci. Rep. *6*, 38177.

Sabin, C., Corti, D., Buzon, V., Seaman, M.S., Lutje Hulsik, D., Hinz, A., Vanzetta, F., Agatic, G., Silacci, C., Mainetti, L., et al. (2010). Crystal structure and size-dependent neutralization properties of HK20, a human antibody binding to the highly conserved heptad repeat 1 of gp41. PLoS Pathog. *6*, e1001195.

Scheid, J.F., Mouquet, H., Ueberheide, B., Diskin, R., Klein, F., Oliveira, T.Y., Pietzsch, J., Fenyo, D., Abadir, A., Velinzon, K., et al. (2011). Sequence and structural convergence of broad and potent HIV antibodies that mimic CD4 binding. Science *333*, 1633–1637.

Scherer, E.M., Leaman, D.P., Zwick, M.B., McMichael, A.J., and Burton, D.R. (2010). Aromatic residues at the edge of the antibody combining site facilitate viral glycoprotein recognition through membrane interactions. Proc. Natl. Acad. Sci. USA *107*, 1529–1534.

Schibli, D.J., and Weissenhorn, W. (2004). Class I and class II viral fusion protein structures reveal similar principles in membrane fusion (Review). Mol. Membr. Biol. *21*, 361–371.

Schneider, T.D., and Stephens, R.M. (1990). Sequence logos: a new way to display consensus sequences. Nucleic Acids Res. *18*, 6097–6100.

Shen, X., Dennison, S.M., Liu, P., Gao, F., Jaeger, F., Montefiori, D.C., Verkoczy, L., Haynes, B.F., Alam, S.M., and Tomaras, G.D. (2010). Prolonged exposure of the HIV-1 gp41 membrane proximal region with L669S substitution. Proc. Natl. Acad. Sci. USA *107*, 5972–5977.

Shen, X., Duffy, R., Howington, R., Cope, A., Sadagopal, S., Park, H., Pal, R., Kwa, S., Ding, S., Yang, O.O., et al. (2015). Vaccine-induced linear epitope-specific antibodies to simian immunodeficiency virus SIVmac239 envelope are distinct from those induced to the human immunodeficiency virus type 1 envelope in nonhuman primates. J. Virol. *89*, 8643–8650.

Smirnova, Y.G., Risselada, H.J., and Müller, M. (2019). Thermodynamically reversible paths of the first fusion intermediate reveal an important role for membrane anchors of fusion proteins. Proc. Natl. Acad. Sci. USA *116*, 2571–2576.

Sok, D., and Burton, D.R. (2018). Recent progress in broadly neutralizing antibodies to HIV. Nat. Immunol. *19*, 1179–1188.

Stiegler, G., Kunert, R., Purtscher, M., Wolbank, S., Voglauer, R., Steindl, F., and Katinger, H. (2001). A potent cross-clade neutralizing human monoclonal antibody against a novel epitope on gp41 of human immunodeficiency virus type 1. AIDS Res. Hum. Retroviruses *17*, 1757–1765.

Tiller, T., Meffre, E., Yurasov, S., Tsuiji, M., Nussenzweig, M.C., and Wardemann, H. (2008). Efficient generation of monoclonal antibodies from single human B cells by single cell RT-PCR and expression vector cloning. J. Immunol. Methods *329*, 112–124.

Tiwari, P.B., Wang, X., He, J., and Darici, Y. (2015). Analyzing surface plasmon resonance data: choosing a correct biphasic model for interpretation. Rev. Sci. Instrum. *86*, 035001.

Tomaras, G.D., Binley, J.M., Gray, E.S., Crooks, E.T., Osawa, K., Moore, P.L., Tumba, N., Tong, T., Shen, X., Yates, N.L., et al. (2011). Polyclonal B cell

responses to conserved neutralization epitopes in a subset of HIV-1-infected individuals. J. Virol *85*, 11502–11519.

Trama, A.M., Moody, M.A., Alam, S.M., Jaeger, F.H., Lockwood, B., Parks, R., Lloyd, K.E., Stolarchuk, C., Scearce, R., Foulger, A., et al. (2014). HIV-1 envelope gp41 antibodies can originate from terminal ileum B cells that share cross-reactivity with commensal bacteria. Cell Host Microbe *16*, 215–226.

Tuckerman, M.E., Berne, B.J., and Martyna, G.J. (1992). Reversible multiple time scale molecular dynamics. J. Chem. Phys. *97*, 1990–2001.

Wagh, K., Bhattacharya, T., Williamson, C., Robles, A., Bayne, M., Garrity, J., Rist, M., Rademeyer, C., Yoon, H., Lapedes, A., et al. (2016). Optimal combinations of broadly neutralizing antibodies for prevention and treatment of HIV-1 Clade C infection. PLoS Pathog *12*, e1005520.

Walker, L.M., Huber, M., Doores, K.J., Falkowska, E., Pejchal, R., Julien, J.P., Wang, S.K., Ramos, A., Chan-Hui, P.Y., Moyle, M., et al. (2011). Broad neutralization coverage of HIV by multiple highly potent antibodies. Nature *477*, 466–470.

Wardemann, H., Yurasov, S., Schaefer, A., Young, J.W., Meffre, E., and Nussenzweig, M.C. (2003). Predominant autoantibody production by early human B cell precursors. Science *301*, 1374–1377.

Weissenhorn, W., Dessen, A., Harrison, S.C., Skehel, J.J., and Wiley, D.C. (1997). Atomic structure of the ectodomain from HIV-1 gp41. Nature *387*, 426–430.

Williams, L.D., Ofek, G., Schätzle, S., McDaniel, J.R., Lu, X., Nicely, N.I., Wu, L., Lougheed, C.S., Bradley, T., Louder, M.K., et al. (2017). Potent and broad HIV-neutralizing antibodies in memory B cells and plasma. Sci. Immunol. *2*.

Zhang, Y. (2008). I-TASSER server for protein 3D structure prediction. BMC Bioinformatics *9*, 40.

Zwick, M.B., Labrijn, A.F., Wang, M., Spenlehauer, C., Saphire, E.O., Binley, J.M., Moore, J.P., Stiegler, G., Katinger, H., Burton, D.R., et al. (2001). Broadly neutralizing antibodies targeted to the membrane-proximal external region of human immunodeficiency virus type 1 glycoprotein gp41. J. Virol. *75*, 10892–10905.

STAR★METHODS

KEY RESOURCES TABLE

REAGENT or RESOURCE	Source	Identifier
Antibodies		
PE-Cy7 Mouse Anti-Human CD19	BD Biosciences	Cat#341113_clone SJ25-C1
Alexa Fluor 488 goat anti-Human IgM	Invitrogen	Cat#A21215; RRID:AB_2535800
Goat anti-Human IgA FITC	Invitrogen	Cat#A18788; RRID:AB_2535565
Anti-Human CD3 PC5	Beckman Coulter	Cat#A07749
Anti-Human CD38 APC	BD Pharmingen	Cat#555462_clone HIT2 (RUO); RRID:AB_398599
Anti-Human CD14 PC5	Beckman Coulter	Cat#A07765
Anti Human CD27 BV650	BioLegend	Cat#302827_clone O323; RRID:AB_11124941
AffiniPure F(ab')$_2$ Fragment Goat Anti-Human IgA+IgG+IgM (H+L)	Jackson Immunoresearch	Cat#109-006-064; RRID:AB_2337548
AP-conjugated anti-human IgG secondary antibody	Southern Biotech	Cat#2040-04; RRID: AB_2795643
10E8 recombinant mAb	IRB and Pasteur; Huang et al., 2012	PMID:23151583; RRID:AB_2491067
HK20 recombinant mAb	IRB; Sabin et al., 2010	PMID:21124990; RRID:AB_2491018
4E10 recombinant mAb	IRB and Pasteur; Stiegler et al., 2001	PMID:11788027; RRID:AB_2491029
LN01 Recombinant mAb and All Variants	this paper	N/A
Mouse Anti-HIV-1 Gag FITC (Clone KC57)	Beckman Coulter	Cat#6604665
mGO53	Dr. Hugo Mouquet, Institut Pasteur, Paris; Wardemann et al., 2003	PMID:12920303
3BNC117	Produced by Dr. Hugo Mouquet; Scheid et al., 2011	PMID: 21764753; RRID:AB_2491033
10–1074	Produced by Dr. Hugo Mouquet; Mouquet et al., 2012	PMID: 23115339; RRID:AB_2491062
PGT128	Produced by Dr. Hugo Mouquet; Walker et al., 2011	PMID:21849977; RRID:AB_2491047
PGT151	Produced by Dr. Hugo Mouquet; Falkowska et al., 2014	PMID: 24768347
Bacterial and Virus Strains		
HIV-1 PV strain CE1176	Fraunhofer Institute for Biomedical Engineering IBMT	N/A
HIV-1 PV strain BJOX2000	Fraunhofer Institute for Biomedical Engineering IBMT	N/A
HIV-1 PV strain X1632	Fraunhofer Institute for Biomedical Engineering IBMT	N/A
HIV-1 PV strain 25710	Fraunhofer Institute for Biomedical Engineering IBMT	N/A
HIV-1 PV strain TRO.11	Fraunhofer Institute for Biomedical Engineering IBMT	N/A
HIV-1 PV strain CH119	Fraunhofer Institute for Biomedical Engineering IBMT	N/A
HIV-1 PV strain 246F3	Fraunhofer Institute for Biomedical Engineering IBMT	N/A
HIV-1 PV strain CE0217	Fraunhofer Institute for Biomedical Engineering IBMT	N/A

(*Continued on next page*)

Continued

REAGENT or RESOURCE	Source	Identifier
HIV-1 PV strain CNE55	Fraunhofer Institute for Biomedical Engineering IBMT	N/A
SVA-MLV_control PV	Fraunhofer Institute for Biomedical Engineering IBMT	N/A
Epstein Barr Virus	IRB	N/A
pCH058.c/2960	NIH AIDS Reagent Program	Cat#11856
pCH077.t/2627	NIH AIDS Reagent Program	Cat#11742
pNL(AD8)	NIH AIDS Reagent Program	Cat#11346
pYU2	NIH AIDS Reagent Program	Cat#1350
Standard panel of reference Env pseudoviruses	Huang et al., 2016	Multiclade Reference Panel PMID:27315479
OverExpress C41(DE3) chemically competent *E. coli* cells	Sigma-Aldrich	Cat#CMC0017
Ni-NTA Agarose	Qiagen	Cat#30210
Biological Samples		
HIV-1 Infected ART Naïve Patient LNMCs	Hospital “Sacco” in Milan, Italy	N/A
Human serum	Etablissement Français du Sang	N/A
Chemicals, Peptides, and Recombinant Proteins		
28amino acids long peptide gp41 MPER	Pepscan	N/A
Human recombinant IL-6	BD Pharmingen	Cat#550071
Human recombinant IL-21	ImmunoTools	Cat#11340215
Human recombinant IL-2	IRB	N/A
MPER-TM1 Peptide	Smart Bioscience	www.smart-bioscience.com
Fos-Choline-12	anatrace	Cat#F308
n-Octyl-β-D-glucosid n-Octyl-β-D-Glucopyranoside	anatrace	Cat#O311
PEG3350	Sigma-Aldrich	Cat#88276
PEG8000	Sigma-Aldrich	Cat#89510
HEPES	Sigma-Aldrich	Cat#H4034
Ethylene glycol	Carl Roth	Cat#6881.1
06:0 PS	Avanti Polar Lipids	Cat#840030
Critical Commercial Assays		
ImmunoWELL cardiolipin (IgG) test	GenBio	Cat#3090
Pierce Rapid Gold BCA Protein Assay kit	Thermo Fisher Scientific	Cat#A53225PR
NK cell isolation kit	Miltenyi Biotec	Cat#130-092-657
LIVE/DEAD Fixable Aqua Dead Cell Stain Kit	Invitrogen	Cat#L34965
ANA HEp-2 Test System	Zeus Scientific	Cat# FA2400
EZ-LinkNHS-PEG4-Biotinylation Kit	Thermo Scientific	Cat#21455
Deposited Data		
MPER-TM1-LN01 complex	this paper	6SNC
MPER-TM1-LN01-PS complex	this paper	6SND
MPER-TM2-LN01 complex	this paper	6SNE
Experimental Models: Cell Lines		
TZM-bl cell line	NIH AIDS Reagent Program	Cat#8129
TZM-bl FcγRI cell line	NIH AIDS Reagent Program	Cat#11798
Expi293F cells	Thermo Fisher Scientific	Cat#A14527
Mesenchimal stromal cells (MSC)	IRB	N/A
CEM-NKR-CCR5	NIH AIDS Reagent Program	Cat# 4376
Raji	ATCC	CCL-86

(*Continued on next page*)

Continued

REAGENT or RESOURCE	Source	Identifier
293T/17	ATCC	Cat# CRL-11268
Experimental Models: Organisms/Strains		
Mouse: homozygous human FcRn (huFcRn) transgenic mice_Tg276 strain	Jackson Laboratory	JAX: 004919
Oligonucleotides		
CpG 2006	Microsynth	N/A
Recombinant DNA		
Plasmid: IgG1 expression vector	Tiller et al., 2008	PMID:17996249
Plasmid: Igk expression vector	Tiller et al., 2008	PMID:17996249
pMX-YU2-ENVΔCT- IRES-GFP	Provided by Dr. Hugo Mouquet, Pasteur	N/A
Plasmid: MPER-TM2 expression vector	This study	N/A
Software and Algorithms		
Prism 8	GraphPad	https://www.graphpad.com/scientific-software/prism/
CLC Main Workbench 8	QIAGEN	https://www.qiagenbioinformatics.com/products/clc-main-workbench/
IMGT-the International Immunogenetics Information System	Lefranc et al., 1999	http://www.imgt.org
XDS	Kabsch, 2010	PMID:20124693
Phaser	McCoy et al., 2007	PMID:19461840
COOT	Emsley et al., 2010	PMID:20383002
REFMAC	Murshudov et al., 1997	PMID:15299926
Phenix	Adams et al., 2010	PMID:20124702
SBGrid	Morin et al., 2013	N/A
Pymol	Warren DeLano	www.pymol.org
VMD (Version 1.9.4)	Humphrey et al., 1996	https://www.ks.uiuc.edu/Research/vmd/
NAMD (Version 2.13)	Phillips et al., 2005	https://www.ks.uiuc.edu/Research/namd/
CHARMM-GUI	Jo et al., 2008	http://www.charmm-gui.org
WebLogo	Schneider and Stephens, 1990	https://weblogo.berkeley.edu/logo.cgi
wwPDB OneDep System		https://deposit-pdbe.wwpdb.org
wwPDB Validation Service		https://validate.wwpdb.org/
BLI Data Analysis software (v11.1.0.25)	Pall FortéBio	https://www.moleculardevices.com
SBGrid Consortium	(Morin et al., 2013)	https://sbgrid.org/
Python 3.7		https://python.org
Biacore T200 Control Software		https://www.gelifesciences.com/biacore

LEAD CONTACT AND MATERIALS AVAILABILITY

Plasmids and mAbs generated in this study will be made available on request but may require a payment and/or a completed Material Transfer agreement if there is potential for commercial application. Further information and requests for resources and reagents should be directed to and will be fulfilled by the Lead Contact, Winfried Weissenhorn (winfried.weissenhorn@ibs.fr).

EXPERIMENTAL MODEL AND SUBJECT DETAILS

Cell Lines

TZM-bl wild-type and TZM-bl expressing the FcγRI cells were obtained from NIH-AIDS Research and Reference Reagent Program (ARRRP) and used for neutralisation assays. TZM-bl wild type cells were maintained in Dulbecco's modified Eagle's medium supplemented with 10% fetal bovine serum, 100 units of Penicillin and 0.1 mg/ml of Streptomycin while TZM-bl expressing the FcγRI cells were maintained in Dulbecco's modified Eagle's medium supplemented with 10% fetal bovine serum, 0.025M Hepes, 50 μg/ml of Gentamicin, 20 μg/ml of Blasticidin. Both cell lines were cultivated at 37°C in a humidified 5% CO_2 incubator. Cell monolayers were split at 1x10^6 cells/T175 flask at confluence by treatment with 0.25% trypsin. Raji-Env cells (obtained from the ATCC,

ATCC® CCL-86™) were cultured in RPMI supplemented with 10% FCS and 1% penicillin/streptomycin at 37°C in 5% CO2 and were used for CDC experiments. Expi293F cells (ThermoFisher Scientific) were maintained in Expi293 Expression Medium and cultivated at 37°C in a humidified 8% CO_2 incubator (shaker with 25-mm shaking diameter, speed set to 140 rpm). Cells were split, once they reached a density of approximately 1-4x10^6 cells/ml, at 0.3x10^6 cells/ml. Expi293F cells were used for Ab production. The sex of all cell lines is female.

HIV-1 Primary Viruses

NLAD8, YU2 and transmitted-founder HIV-1 strains (CH058, CH077; obtained from the NIH AIDS Reagent Program) were prepared by the transfection of 293T cells (obtained from the ATCC) along with vesicular stomatitis virus G (VSV-G) to normalize infectivity, as done previously (Casartelli et al., 2010). HIV-1 PVs were provided by the Fraunhofer Institute for Biomedical Engineering IBMT.

Sample Collection

Male donor SA003 (year of birth 1963), ART naïve, was selected among a cohort of HIV-1-infected patients enrolled in the Hospital "Sacco" in Milan due to the ability of its plasma to neutralize eight out of nine HIV-1 PVs of the Global Panels. The aforementioned donor is a Long Term Non-Progressor (LTNP) patient who consented to lymphadenectomy, signed informed written consent and underwent surgery. At that time, he had been infected with HIV-1 for 30 years with CD4 T-cell counts of 474 cells/μl and plasma HIV-1 RNA values of 1600 copies/ml, CDC stage A1. The donor was not involved in previous procedures and he had never received antiretroviral therapy. The protocol was approved by the Ethic Commission Milan Area A (Comitato Etico Interaziendale Milano Area A) protocol Number 0006143 on February 20th 2015.

Animals

Treatment-naive female, 6-10 weeks old, homozygous human FcRn (huFcRn) transgenic mice (Tg276 strain, Jackson Laboratory Bar Harbor, ME) were used to assess the pharmacokinetics of monoclonal antibodies. Mice were bred in the specific pathogen-free (spf) facility at the Institute for Research in Biomedicine Switzzerland under a chow diet. All the *in vivo* procedures were performed in accordance with the Swiss Federal Veterinary Office guidelines and as authorized by the Cantonal Veterinary Office.

METHODS DETAILS

B-cell Isolation and Stimulation

Memory and GC B cells from patient SA003 were isolated from cryopreserved lympho node mononuclear cells (LNMCs) as follows: LNMCs were stained with anti-human CD19 PE-Cy7 (BD Bioscience_341113), anti-human IgM FITC (Invitrogen_A21215), anti-human IgA FITC (Invitrogen_A18788), anti-human CD27 BV650 (Biolegend_302827), anti-human CD38 APC (Beckman Coulter_555462) and anti-human CD14 PC5 (Beckman Coulter_A07765) plus anti-human CD3 PC5 (Beckman Coulter_A07749) on ice for 20 min. The cells were then washed, filtered and sorted using FACSAria (Becton Dickinson). Memory IgG$^+$ B cells were sorted as CD19$^+$CD27$^+$CD38$^-$ while GC IgG$^+$ B cells as CD19$^+$CD27$^+$CD38$^+$, both negative for all other markers, resuspended in complete IMDM with 10% FBS and immortalized with Epstein-Barr virus (EBV) in the presence of 2.5 μg/ml CpG 2006, 2.5 μg/ml AffiniPure F(ab')$_2$ Fragment Goat Anti-Human IgA+IgG+IgM (H+L) (Jackson Immunoresearch), 500 U/ml IL-2, 5 ng/ml IL-6 (BD Pharmingen) and 10 ng/ml IL-21 (ImmunoTolls). The two subsets of B cells were seeded at 0.5 cells per well on a monolayer of Mesenchimal Stromal Cells (MSC) (Corti et al., 2014; Pinto et al., 2013) in 384-well microtiter plates. After 2 weeks, the supernatants were screened for neutralization activity using high throughput micro-neutralization assay against BJOX and CE1176 HIV-1 PVs. The B-cell cultures that neutralized both the PVs were then re-tested in secondary screening against the same PVs plus other two: X1632 and 25710. The B cells that neutralized four out of four HIV-1 PVs were lysed and the variable regions of the heavy and light chain were cloned.

Sequence Analysis of Antibody cDNA

cDNA was synthesized from selected B-cell culture and both the heavy and light chain variable regions (VH and VL) were sequenced as previously described (Tiller et al., 2008). Using the Database: IMGT (http://www.imgt.org), the VH and VL gene family and the number of somatic mutations were determined by analyzing the homology of the VH and VL sequences to known human V, D and J genes. UCA sequences of the VH and VL were constructed using IMGT/V-QUEST.

Production of Recombinant Antibody and Antibody Variants

The variable regions of the heavy and light chain were cloned into IgG1 and IgK expression vectors (Tiller et al., 2008) and expressed recombinantly by transient transfection of Expi293F cells (ThermoFisher Scientific) using polyethylenimine (PEI). After 7 days, cell culture supernatants were collected, centrifuged, filtered and purified by protein A chromatography (GE Healthcare). The purified antibodies were quantified using Pierce Rapid Gold BCA Protein Assay kit (ThermoFisher Scientific).

Neutralization Assays

A single-cycle infectivity assay was used to measure the neutralization of luciferase-encoding virions pseudotyped with the desired HIV-1 Env-protein. For the primary screening, the culture supernatants from day 14 were tested using 384-well based HIV-1 PVs microneutralization assay using in parallel two strains, CE1176 and BJOX2000 representative respectively of clade C and CRF07.

Neutralization were undertaken on TZM-bl (3000 cell per well). Briefly, 10μl of culture supernatant was pre-incubated with 10 μl of diluted HIV-1 PVs for 1h at 37° (5% CO_2) and then added on the top of TZM-bl. After an incubation of 72h at 37°, the cells were lysed with BriteLite reagent (PerkinElmer) and the luciferase activity detected using the EnVision multimode plate reader (PerkinElmer). For the secondary screening, the neutralization was performed following the same protocol but making four dilution of the culture supernatant before adding the HIV-1 PVs (CE1176, BJOX2000, X1632 and 25710). To test the breadth and potency of the recombinant antibodies, the neutralization assay was performed in 96-well plates. Briefly, 35μl of serial diluted mAb were pre-incubated with 35μl of HIV-1 PVs for 1h at 37° and then added on TZM-bl or TZM-bl expressing FcγRI, depending on the experiment, seeded the day before at 10 000 cell per well. The volume of medium was then adjusted to 200 μl per well the day after. After an incubation of 72h at 37°, the cells were lysed, and the luciferase activity measured using the EnVision. IC_{50} and IC_{80} were calculated by nonlinear regression analysis using the GraphPad Prism 5 software.

Neutralization against the extended panel of HIV-1 PVs (Huang et al., 2016) was employed to evaluate breadth and potency. Neutralization was assessed after a single round of infection in either TZM-bl or TZM-bl/FcγR1 cells using non-reported PV as described (Montefiori, 2009). A pre-titrated dose of PV was incubated with serial 3-fold dilutions of test sample in duplicate in a total volume of 150 μl for 1 hr at 37°C in 96-well flat-bottom culture plates. Freshly trypsinized cells (10,000 cells in 100 μl of growth medium containing 75 μg/ml DEAE dextran) were added to each well. One set of 8 control wells received cells + virus (virus control) and another set received cells only (background control). After 48 hours of incubation, 100 μl of cells was transferred to a 96-well black solid plate (Costar) for measurements of luminescence using the Britelite Luminescence Reporter Gene Assay System (PerkinElmer Life Sciences). Assay stocks of PV were prepared by transfection in 293T/17 cells (American Type Culture Collection) and titrated in TZM-bl cells as described (Montefiori, 2009).

ADCC Assay

HIV-1-infected target CEM-NKR.CCR5 CD4 T cells were stained using the Far Red DDAO cell tracker (Life Technologies). 2–5 $\times 10^4$ target cells were plated in U-bottom 96-well plates and incubated with antibodies (15 μg/ml) for 5 min at room temperature. NK cells were added in each well (at a ratio of 1 CEM-NKR-CCR5 : 10 NK). Plates were spun 1 min at 300 g to promote cell contacts and incubated at 37°C for 4 h. Cells were then stained for intra-cellular Gag with the anti-Gag KC57 murine monoclonal antibody. To measure cell viability, the live/dead fixable aqua dead cell marker (1: 1,000 in PBS, Life Technologies) was added 20 min at 4°C before fixation. Data were acquired on a BD FACS CANTO II and analysed using FlowJo software. The frequencies of Gag+ cells among Far-Red+ cells were determined. ADCC was calculated using the following formula: 100 × (% of Gag+ target cells plus NK without antibody - % of Gag+ target cells plus effector with antibody)/(% of Gag+ target cells plus NK without antibody). Negative values were set to zero. Antibodies used in this assay, 3BNC117 (Scheid et al., 2011), 10-1074 (Mouquet et al., 2012), PGT128 (Walker et al., 2011), PGT151 (Falkowska et al., 2014), 4E10 (Stiegler et al., 2001), 10E8 (Huang et al., 2012) and mGO53 (Wardemann et al., 2003) were produced as described above.

Complement-dependent Cytotoxicity Assay

Raji-Env cells were generated with Raji cells (obtained from the ATCC, ATCC® CCL-86™) that were spinoculated (1,000g for 1,5h at 32°C) with a retroviral viral vector carrying Env (pMX-YU2 ENVΔCT-GFP-PuroR) (Pietzsch et al., 2010). Transduced cells (GFP+) were sorted and cultivated in the presence of Puromycin (1μg/ml, Sigma). High level of Env expression was then obtained by subcloning. 0.5 x 105 Raji-Env Cells were cultivated in presence of 50% normal human serum (NHS) or heat-inactivated human serum (HIHS) and with or without antibodies (15 μg/ml). After 24h, cells were washed twice with PBS and stained with the live/dead fixable aqua dead cell marker (1:1,000 in PBS, Life Technologies) for 30 min. Cells were then fixed with 4% PFA for 10min at RT. The frequency of dead cells was measured by flow cytometry (Attune Nxt, ThermoFischer). CDC was calculated using the following formula: 100 x (% of dead cells with antibody - % of dead cells without antibody)/(100 - % of dead cells without antibody).

Pharmacokinetic Analysis

LN01 pharmacokinetic was performed in Tg276 huFcRn transgenic mice in parallel with palivizumab and a control mAb specific for an irrelevant antigen. The mAbs were administered i.v. at 10 mg/ml (number of mice per group = 5). The concentration of human IgG mAbs in plasma were determined at multiple time points: 1-, 5-, 9-, 13- and 16-days post-administration. Mice bleeding was performed from the tail vein of mice.

Autoreactivity Assays

The LN01 mAb autoreactivity was tested by indirect immunofluorescence on human HEp-2 cells and by ELISA against a self-antigen, the human cardiolipin. In the first approach, LN01 mAb was tested at 50 μg/ml on slides coated with HEp-2 cells in parallel to a negative control and the positive control provided by the kit as well as 4E10 and 10E8 at 50 μg/ml. All the procedure was performed according to the kit specifications (Zeus Scientific). In the second approach, the anti-cardiolipin ELISA was performed according to the kit instructions (GenBio). Serial dilutions of the mAbs were tested on the pre-coated 96-well plate.

ELISA Assay

The binding of LN01, 10E8 and HK20 (Sabin et al., 2010) mAbs to the 28 amino acids long peptide encompassing the entire gp41 MPER was tested by ELISA using 96 well plates half-area. Briefly, ELISA plates were coated with 2 μg/ml of the long peptide

(Pepscan), blocked with 1% BSA and incubated with titrated antibodies, followed by AP-conjugated anti-human IgG secondary antibody (SouthernBiotech). Plates were then washed and substrate (p-NPP, Sigma) was added. After 1h of incubation, plates were read at 405 nm. For human IgG quantification in the plasma of mice from *in vivo* experiment, 96 well plates were coated with 10 μg/ml of goat anti-human IgG UNL (SouthernBiotech), blocked with 1% BSA and incubated with titrated plasma, followed by AP-conjugated anti-human IgG secondary antibody (SouthernBiotech). Plates were then washed and substrate (p-NPP, Sigma) was added. After 30 min of incubation, plates were read at 405 nm. As standard to quantify human IgG concentration, serial dilutions of Rituximab were used.

Linear Epitope Mapping

Linear epitope mapping was performed by peptide microarray as previously reported (Gottardo et al., 2013; Shen et al., 2015). The peptides were printed onto 3D-Epoxy glass slides and were analyzed with a GenePix 4000B scanner (Tomaras et al., 2011). The binding of LN01 and 7B2 was detected by incubation with DyLight 649-labeled goat anti-human IgG. Fluorescence intensity was measured using a GenePix 4000B scanner and was analyzed with GenePix software. Signal intensity is the median fluorescence intensity of triplicate spots for each peptide included on array slides.

Gp41 Peptides

The MPER-TM1 peptide (671-NWFDITNWLWYIKLFIMIV-KKKKKK-689) was synthesized (Smart Bioscience, Grenoble). MPER-TM2 contains an N-terminal His-tag, a flag-tag a TEV protease site and gp41 residues 630 to 711 similar to the construct described before (Lutje Hulsik et al., 2013). For crystallization, the construct was engineered to contain a second TEV protease site inserted at position 649, which produced gp41-TM2 containing residues 650 to 711. The cDNAs were cloned into petM20 and expressed in C41 *E. coli* cells for 3h at 37º. Cells were lysed by sonication in a buffer containing 20 mM Tris pH8.0, 100 mM NaCl, 1% CHAPS. The soluble fraction was passed over a Ni^{2+}-chromatography column. Purified gp41 was cleaved by TEV protease at a ratio (w/w) 1:10 passed over an anion exchange column followed by a second Ni^{2+}-chromatography column to remove non cleaved protein. The resulting MPER-TM2 was further purified by size exclusion chromatography on a S200 column in a buffer containing either 25 mM Hepes pH7.5, 150 mM NaCl, 3 mM Fos-Choline-12 or 25 mM Hepes pH7.5, 150 mM NaCl, 1% β-OG (n-Octyl-β-D-glucosid). For complex formation of MPER-TM2 with LN01 Fabs, the complex was purified by SEC on a superdex 200 column in a buffer containing 25 mM Hepes pH7.5, 150 mM NaCl, 3 mM Fos-Choline-12. The complex was concentrated to 10 mg/ml. For complex formation of the MPER-TM1 peptide with LN01 Fabs, the peptide was solubilized in a buffer containing 25 mM Hepes pH7.5, 150 mM NaCl, 6 mM Fos-Choline-12 at 2 mg/ml, mixed with LN01 Fabs in a buffer containing 25 mM Hepes pH7.5, 150 mM NaCl, 3 mM Fos-choline-12 and concentrated to 10 mg/ml. The peptide was added to the LN01 Fabs at the molar ratio of 1:1.5 (Fab : MPER-TM1 peptide).

Surface Plasmon Resonance (SPR) Analysis

Biacore T200 (GE Healthcare, USA) was used for real-time binding interaction studies. Antibodies LN01 and 10E8 were immobilized via amine coupling on CM5 chips (GE Healthcare) in Hepes buffer (pH 7.5) at a flow rate of 10 μl/min until the surface plasmon resonance reached 2000 RU. Analyte concentrations varied from 1 to 2048 nM. Different dilutions of analytes were sequentially injected at a flow rate of 90 μl/min for 180s. MPER-TM1 and MPER-TM2 analytes were applied in a buffer containing 20 mM Tris pH 7.5, 100 mM NaCl, and either 1% β-OG (n-Octyl-β-D-glucosid) or 3 mM Fos-Choline-12. The dissociation time was set for 5 min and 0.1% SDS was injected for regeneration of the sensor chips. The sensogrammes were processed and visualized with Python. SPR profiles were fit to a double exponential equation. The model of biphasic binding was identified as the two state conformational change model, since the concentration dependencies of eigenvalues were linear. Dissociation constants were calculated as described (Tiwari et al., 2015).

Bio-layer Interferometry Binding Analysis

Binding measurements between LN01, LN01 variants and MPER-TM2 were carried out on an Octet Red instrument (ForteBio). For the determination of the binding between LN01 IgG and MPER-TM2, LN01 IgG were labelled with biotin (EZ-Link NHS-PEG4-Biotin) and bound to Streptavidin (SA) biosensors (ForteBio). The biosensors loaded with IgG were equilibrated in the kinetic buffer (25 mM HEPES pH 7.5, 150 mM NaCl and 0.1 % Fos-Choline-12) for 120 sec prior to measuring association with MPER-TM2 for 200 seconds at 25°C. Data were analyzed using the ForteBio analysis software version 11.1.0.25 (ForteBio). For LN01 sH/sL, LN01 gH/sL and LN01 sH/gL-FR the kinetic parameters were calculated using a global fit 1:1 model. For the determination of the binding of LN01 sH/sL, LN01 gH/gL and LN01 gH/UCA, K_D were estimated by steady state analysis. For LN01 UCA/sL, LN01 UCA/gL and LN01 UCA/UCA no binding was detected in this experimental setup. All bio-layer interferometry experiments were conducted at least three times.

Crystallization, Data Collection and Structure Determination

Crystal screening was performed at the High Throughput Crystallisation Laboratory (HTX lab, EMBL Grenoble) in 96-well sitting drop vapour diffusion plates (Greiner). Following optimization, crystals used for diffraction studies were grown at 20°C (293 K) in hanging drop vapour diffusion plates. The LN01/MPER-TM1 complex was crystallized in 0.1 M HEPES pH 7.5, 10 %(w/v) PEG 3350. Crystal were grown by mixing 1 μl of the reservoir containing 0.1 M HEPES pH 7.5, 10 %(w/v) PEG 3350, 0.1%(w/v) Fos-Choline-12 and 1 μl

of protein at a concentration of 8 mg/ml. The crystal was soaked into a cryo protectant solution containing 0.1 M HEPES pH 7.5, 5 % (w/v) PEG 3350, 30 %(w/v) Ethylene glycol, 0.1 %(w/v) Fos-Choline-12, and flash cooled in liquid N2 at 100 K. For LN01/MPER-TM1, data were collected on the ESRF beamline ID30A at a wavelength of 0.9677 Å. Data were processed with the program XDS (Kabsch, 2010). The LN01/MPER-TM1 crystals belong to space group P 4_3 2_1 2 (Table S2) and the structure was solved by molecular replacement using the program Phaser (McCoy et al., 2007) and a model of the LN01 Fab generated by I-Tasser (Zhang, 2008). The model was rebuilt using COOT (Emsley et al., 2010) and refined using REFMAC (Murshudov et al., 1997) and Phenix (Adams et al., 2010). Statistics for data reduction and structure refinement are presented in Table S2.

The crystals of LN01/MPER-TM1 plus 1,2-dihexanoyl-sn-glycero-3-phospho-L-serine (06:0 PS) were obtained by adding 1 mM of 06:0 PS in the protein solution and in the cryoprotectant solution. X-ray data were collected on the ESRF beamline ID23eh1, at a wavelength of 0.98 Å and data were processed with the program XDS (Kabsch, 2010).

The LN01/MPER-TM2 complex was crystallized by mixing 1 μl of 10 mg ml-1 of the LN01/MPER-TM2 complex and 1 μl of the reservoir solution containing 0.1 M HEPES pH 7.5, 9 %(w/v) PEG 8000, 8% Ethylene glycol, 10 mM $MnCl_2$. For cryo protection, crystals were soaked in 0.1 M HEPES pH 7.5, 9 %(w/v) PEG 8000, 25 %(w/v) Ethylene glycol, 10 mM $MnCl_2$, 0.1 %(w/v) Fos-Choline-12 prior to flash cooling in liquid N2 at 100 K. X-ray data were collected on the ESRF beamline ID30B at a wavelength of 0.9763 Å and data were processed with the program XDS (Kabsch, 2010). The LN01/MPER-TM2 crystals belong to space group P 2_1 2_1 2_1 (Table S2). The structure was solved by molecular replacement using the program Phaser (McCoy et al., 2007) and the LN01/MPER-TM1 model coordinates. The model was rebuilt using COOT (Emsley et al., 2010) and refined using REFMAC (Murshudov et al., 1997) and Phenix (Adams et al., 2010). Statistics for data reduction and structure refinement are presented in Table S2. The models were evaluated by using COOT and Phenix validation tools.

Two copies of the LN01/MPER-TM1 complex and 4 copies of the LN01/MPER-TM2 complex are present in the asymmetric units of the respective crystals. Numbering of the Fab was performed according to Kabat. The LN01/MPER-TM1 complex was refined to 3.2 Å data with an R/Rfree of 21.3/25.3%. 96.22% of the residues are within the most favoured regions of a Ramachandran plot. The LN01/MPER-TM1 + 06:0 PS complex was refined to 3.1 Å data with an R/Rfree of 23.8/26.7%. 95.8% of the residues are within the most favoured of a Ramachandran plot. The LN01/MPER-TM2 complex was refined to 3.9 Å data with an R/Rfree of 21.8/26.0% and 95.39% of the residues are within the most favoured regions of a Ramachandran plot. (Table S2). Some of the software packages used in this study were compiled by SBGrid (Morin et al., 2013).

Figure Generation

Molecular graphics figures were generated with PyMOL (W. Delano; The PyMOL Molecular Graphics System, Version 1.8 Schrödinger, LLC, http://www.pymol.org). To determine the angle of approach of antibodies LN01, 10E8, 4E10 and DH511.1 to gp41 MPER a similar method as described (Williams et al., 2017) was employed. Briefly, an axis was drawn from a spatial position midway between the variable region disulfide bond to the Cα atom of residue T676 thereby defining the direction of approach. The representation of the sequence conservation of MPER shown in Figure S6 was generated using WebLogo (Schneider and Stephens, 1990).

Molecular Dynamics (MD) Simulation

The molecular assay consisted of gp41 MPER-TM2 or only TM embedded in a lipid bilayer formed by 20 1-palmitoyl-2-oleoyl-phosphatidylcholine (POPC), 44 1-palmitoyl-2-oleoyl-phosphatidylethanolamine (POPE), 18 1-palmitoyl-2-oleoyl-phosphatidyl serine (POPS), 28 sphingomyelin d18:0/d16:0 (SSM) and 90 cholesterol units in equilibrium with 11,123 water molecules, corresponding to a cell dimension of approximately 66 × 66 × 120 $Å^3$. K^+ and Cl^- ions were added to reach an ionic concentration of 150 mM. All molecular dynamics (MD) simulations reported herein were performed employing the parallel, scalable program NAMD 2.12 (Phillips et al., 2005). Periodic boundary conditions (PBCs) were applied in the three directions of Cartesian space. Water was described by the TIP3P model (Jorgensen et al., 1983), and both gp41 and its lipid environment by the all-atom CHARMM36 force field (Jo et al., 2008; Klauda et al., 2010; MacKerell et al., 1998). A mass repartitioning scheme was introduced, allowing the equations of motion to be integrated with a time step of 4 fs, using the r-RESPA multiple time-step algorithm (Tuckerman et al., 1992). Covalent bonds involving hydrogen atoms were constrained to their equilibrium length by means of the RATTLE (Andersen, 1983) and SETTLE algorithms (Miyamoto and Kollman, 1992). The temperature and the pressure were maintained at 303 K and 1 atm, respectively, using Langevin dynamics and the Langevin piston method (Feller et al., 1995). Long-range electrostatic forces were taken into account by means of the particle mesh Ewald algorithm (Darden et al., 1993). A 12-Å cutoff was applied to truncate van der Waals and short-range Coulombic interactions. Visualization and analyses of the MD trajectories were performed with the VMD program (Humphrey et al., 1996). The free-energy landscape underlying the transition of gp41 from its straight to its bent form was determined using a multiple-walker version (Comer et al., 2014) of the MW-ABF algorithm (Comer et al., 2015) with a reaction coordinate model equal to the difference of the distance root mean square deviations (RMSD) with respect to these two conformations and eight walkers. No assumption was made on the initial orientation of the MPER-TM2 segment. The latter was placed such that its longitudinal axis was aligned with the normal to the lipid bilayer, i.e., the *z*-axis of Cartesian space. Next, the computational assay was suitably thermalized until a plateau in the dimensions of the membrane patch was reached and a steady orientation of the MPER-TM2 segment was observed. The tilt angle reported in this work is, therefore, a consequence of the sequence of the MPER-TM2 segment in relationship with the composition of the lipid bilayer, rather than an initial placement suggested by experiment. The steady orientation of the MPER-TM1 segment served as a starting point for the LN01-MPER-TM2 simulation.

The initial gp41 MPER-TM2 assay was built by aligning gp41 onto its equilibrium conformation sampled from the simulation with the isolated straight TM peptide. Lipids and water molecules were thermalized during 50 ns, while maintaining atoms of the protein complex to their crystallographic positions by means of harmonic restraints. Next, a set of three harmonic restraints over root-mean-square deviation collective variables was used to maintain backbones of LN01 and MPER-TM2 and side chains of LN01/MPER-TM2 contact residues in their reference conformation, respectively. Using this protocol, the orientation of the protein complex with respect to the bilayer was equilibrated during 200 ns. Finally, all the restraints were removed, and a 200 ns trajectory was produced.

QUANTIFICATION AND STATISTICAL ANALYSIS

Antidody EC50 and EC80 and serum ID50 were determined from log-transformed nonlinear regression, dose-response sigmoidal curve fit data using GraphPad 8. Antibody neutralization values higher then 12.5 or 25 μg/ml were considered as negative. The results were reported in Figures 1E and 2A and S1 and S3. Statistical models inherent to REFMAC (Murshudov et al., 1997) and Phenix (Adams et al., 2010) were employed for the structure refinement. All binding and neutralization assays were conducted with at least duplicate measurements. ADCC responses were analyzed in the Wilcoxon test. The results were reported in Figure 3C.

DATA AND CODE AVAILABILITY

All data generated or analyzed during this study are included in this published article (and its Supplemental Information). Atomic coordinates and structure factors of the reported crystal structures have been deposited in the Protein Data Bank (https://www.rcsb.org; PDB: 6SNC, 6SND, 6SNE).

Cell Host & Microbe, Volume *26*

Supplemental Information

Structural Basis for Broad HIV-1 Neutralization by the MPER-Specific Human Broadly Neutralizing Antibody LN01

Dora Pinto, Craig Fenwick, Christophe Caillat, Chiara Silacci, Serafima Guseva, François Dehez, Christophe Chipot, Sonia Barbieri, Andrea Minola, David Jarrossay, Georgia D. Tomaras, Xiaoying Shen, Agostino Riva, Maciej Tarkowski, Olivier Schwartz, Timothée Bruel, Jérémy Dufloo, Michael S. Seaman, David C. Montefiori, Antonio Lanzavecchia, Davide Corti, Giuseppe Pantaleo, and Winfried Weissenhorn

Supplemental Information

Structural basis for broad HIV-1 neutralization by the MPER-specific human broadly neutralizing antibody LN01

Dora Pinto, Craig Fenwick, Christophe Caillat, Chiara Silacci, Serafima Guseva, François Dehez, Christophe Chipot, Sonia Barbieri, Andrea Minola, David Jarrossay, Georgia D. Tomaras, Xiaoying Shen, Agostino Riva, Maciej Tarkowski, Olivier Schwartz, Timothée Bruel, Jérémy Dufloo, Michael S. Seaman, David C. Montefiori, Antonio Lanzavecchia, Davide Corti, Giuseppe Pantaleo, Winfried Weissenhorn

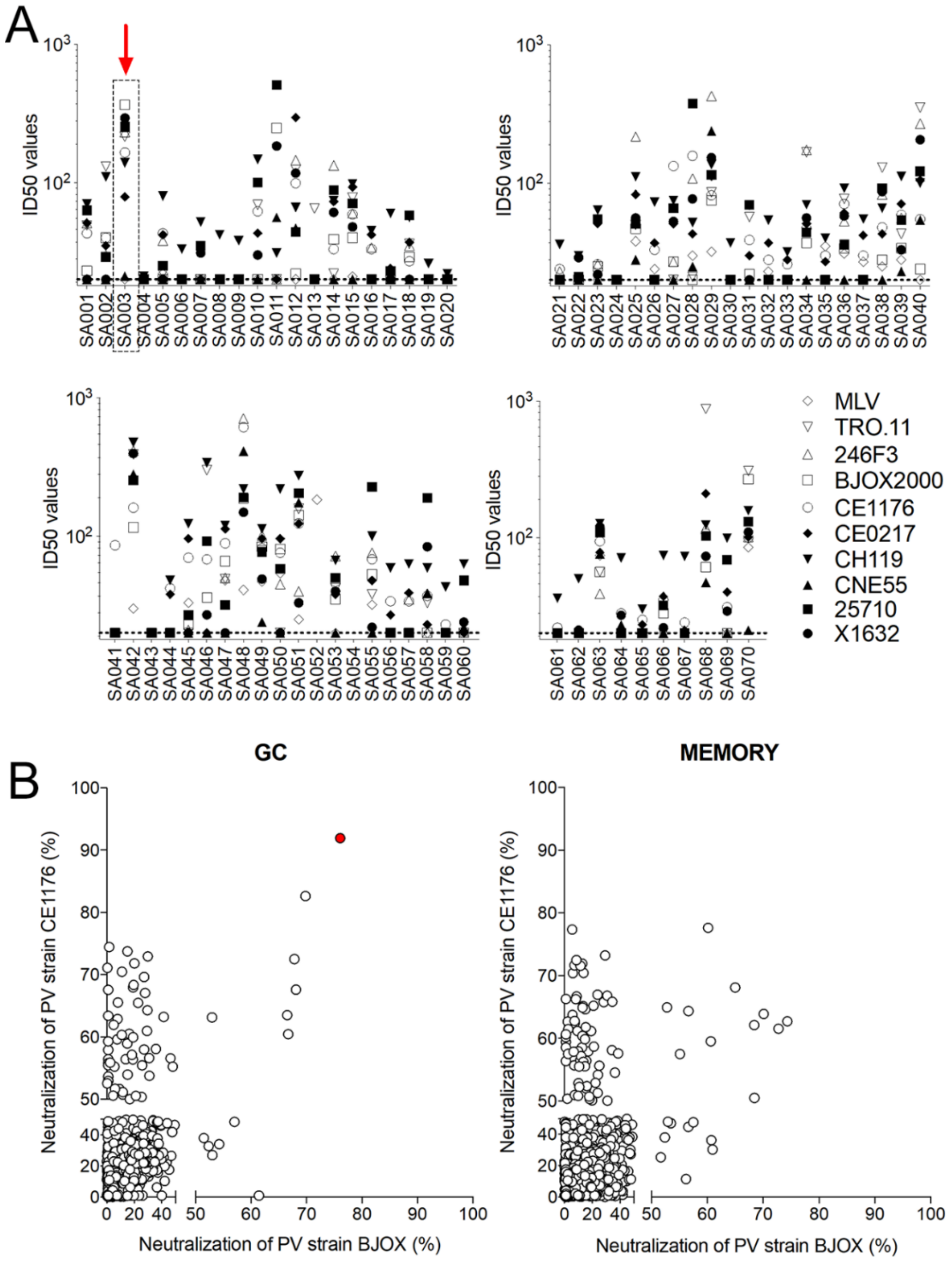

Figure S1 related to Figure 1. Selection of HIV-1 patients for the presence of broad neutralizing activity in the serum and identification of the LN01 mAb.

(A) A cohort (n=70) of patients chronically infected with HIV-1 clade B or clade C were screened for the presence of high titers of antibodies able to neutralize a panel of nine HIV-1 PVs from the Global Panel of HIV-1 reference strains. The sera were tested in neutralization assay using TZM-bl cells and the ID50s (y-axes) were determined.

(B) Identification of LN01 mAb. IgG GC and IgG memory B cells isolated from LNMC of an HIV-1 infected donor were seeded on MSC at 3 c/well in 384-well plates in the presence of IL-2, IL-6, IL-21, BCR-trigger and EBV. After 14 days of culture, supernatants were collected and tested for their ability to neutralize HIV-1 strains CE1176 and BJOX PV using a TZMbl-base micro-neutralization assay.

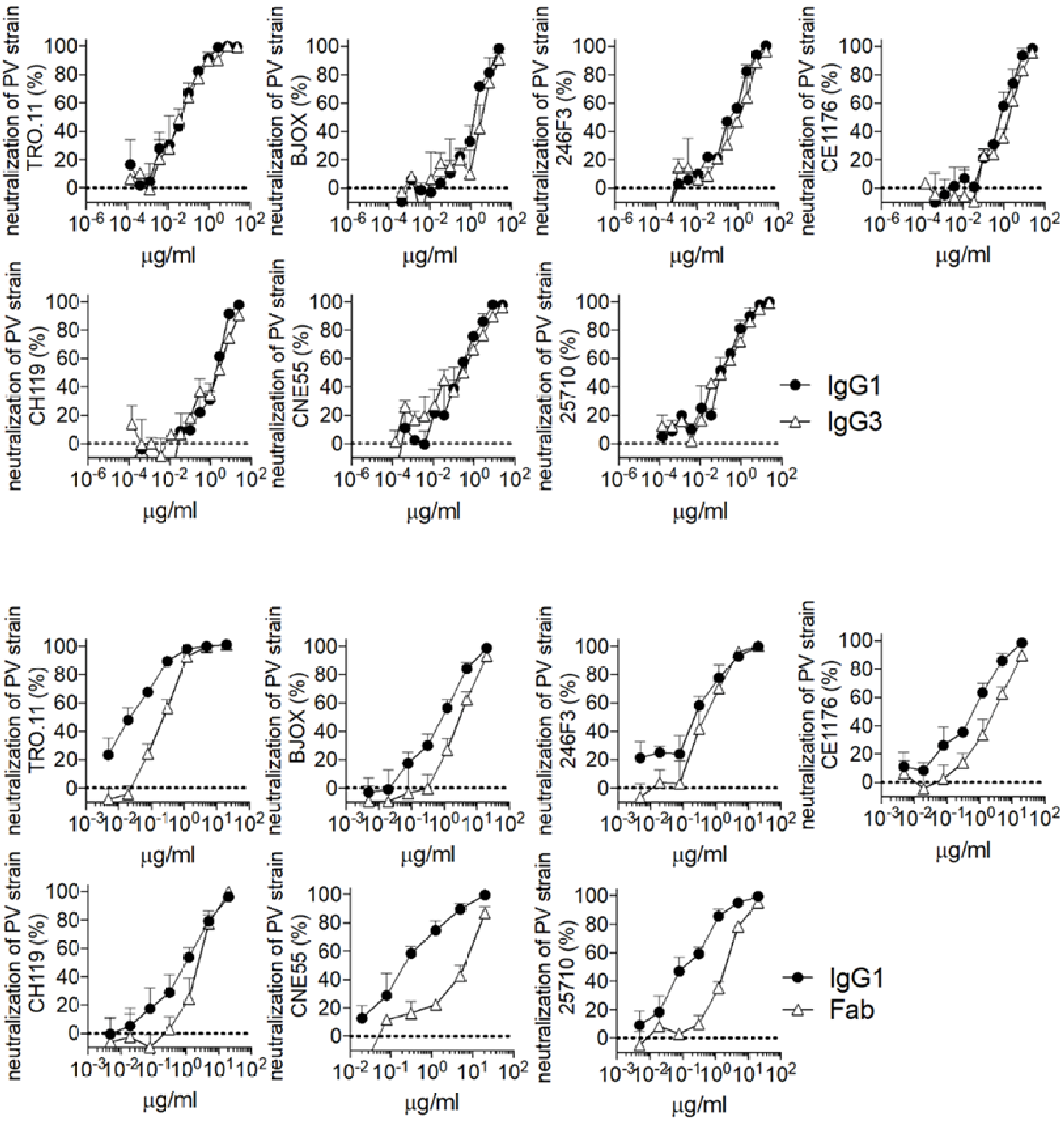

Figure S2 related to Figures 1C and 3A, B. Neutralizing activity of LN01 IgG1, IgG3 and Fab Ig formats.

Percentage of neutralization of LN01 IgG1 compared to LN01 IgG3 (top panel) and LN01 Fab fragment (bottom panel) against seven HIV-1 PVs of the Global Panel. Serial dilutions of the recombinant mAbs were incubated with HIV-1 PVs and then added to the TZM-bl target cells.

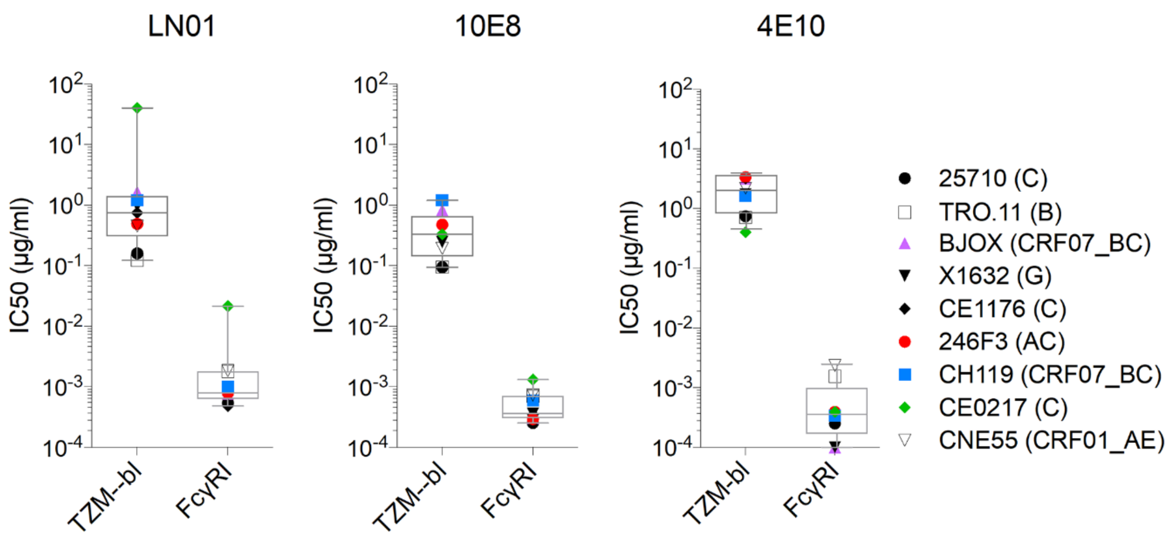

Figure S3 related to Figures 1C and 3A, B. FcγRI expression on target cells greatly enhances HIV-1 neutralization by anti-MPER broadly neutralizing mAbs.

LN01 mAb and the two other anti-MPER mAbs 10E8 and 4E10 were tested for their neutralizing activity against nine HIV-1 PVs of the Global Panel using either parental TZM-bl cells or TZM-bl cells expressing human FcγRI. The IC50 values expressed in µg/ml are shown.

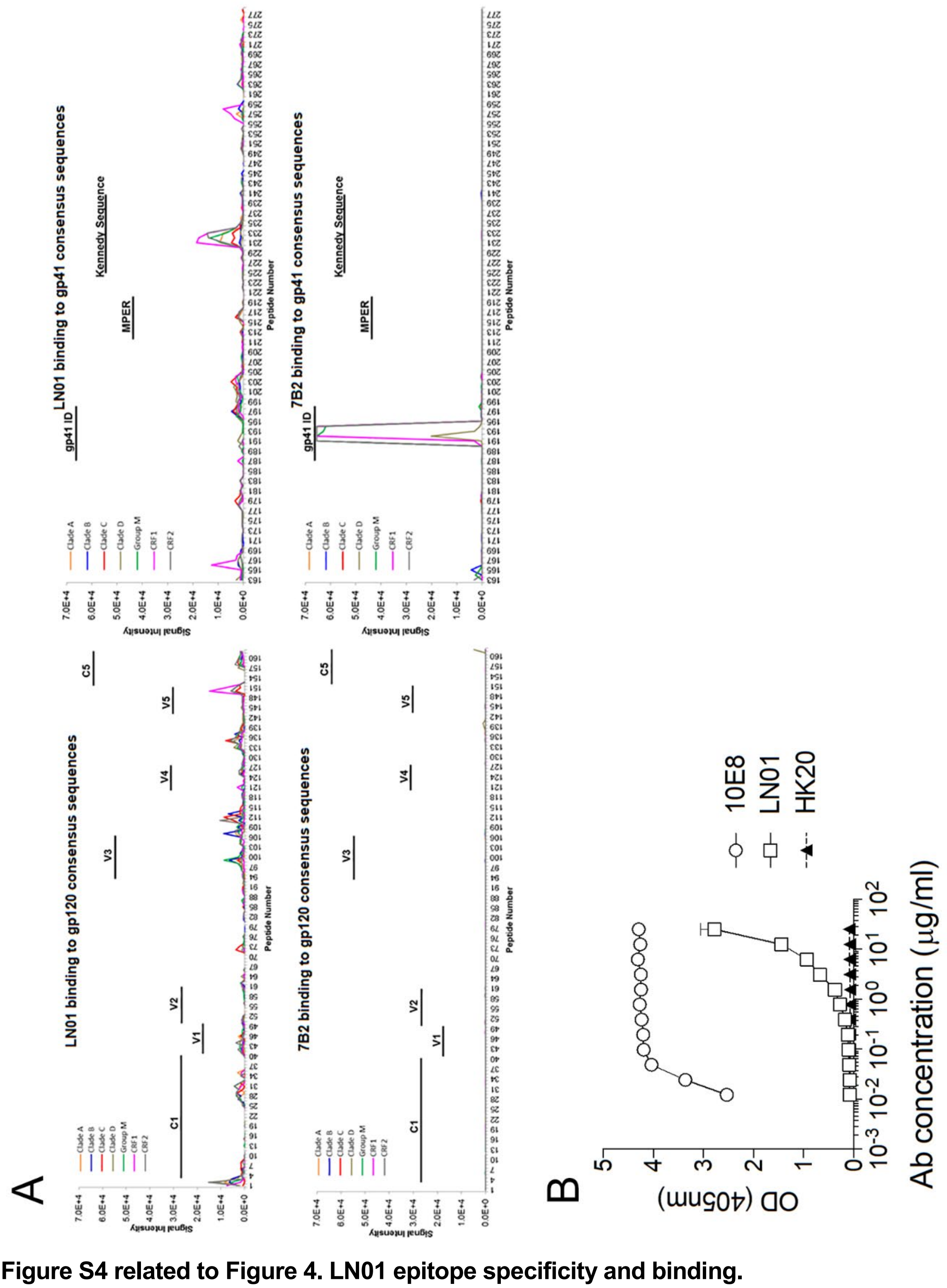

Figure S4 related to Figure 4. LN01 epitope specificity and binding.

(A) Binding of LN01 and 7B2 mAbs to an array of 1423 15-mer peptides, overlapping by 12 amino acids, that cover the full length of the consensus HIV-1 Env gp160 sequences for clades A, B, C, D, group M, CRF01_AE and CRF02_AG. Signals below 2.0E+4 are scored as negative. As expected 7B2 reacts with the gp41 immuno-dominant region.

(B) LN01, 10E8 and HK20 (HR1 specific) mAbs were tested in ELISA for binding to a 28 amino acids long peptide encompassing the entire gp41 MPER. Shown are the OD values at 405 nm.

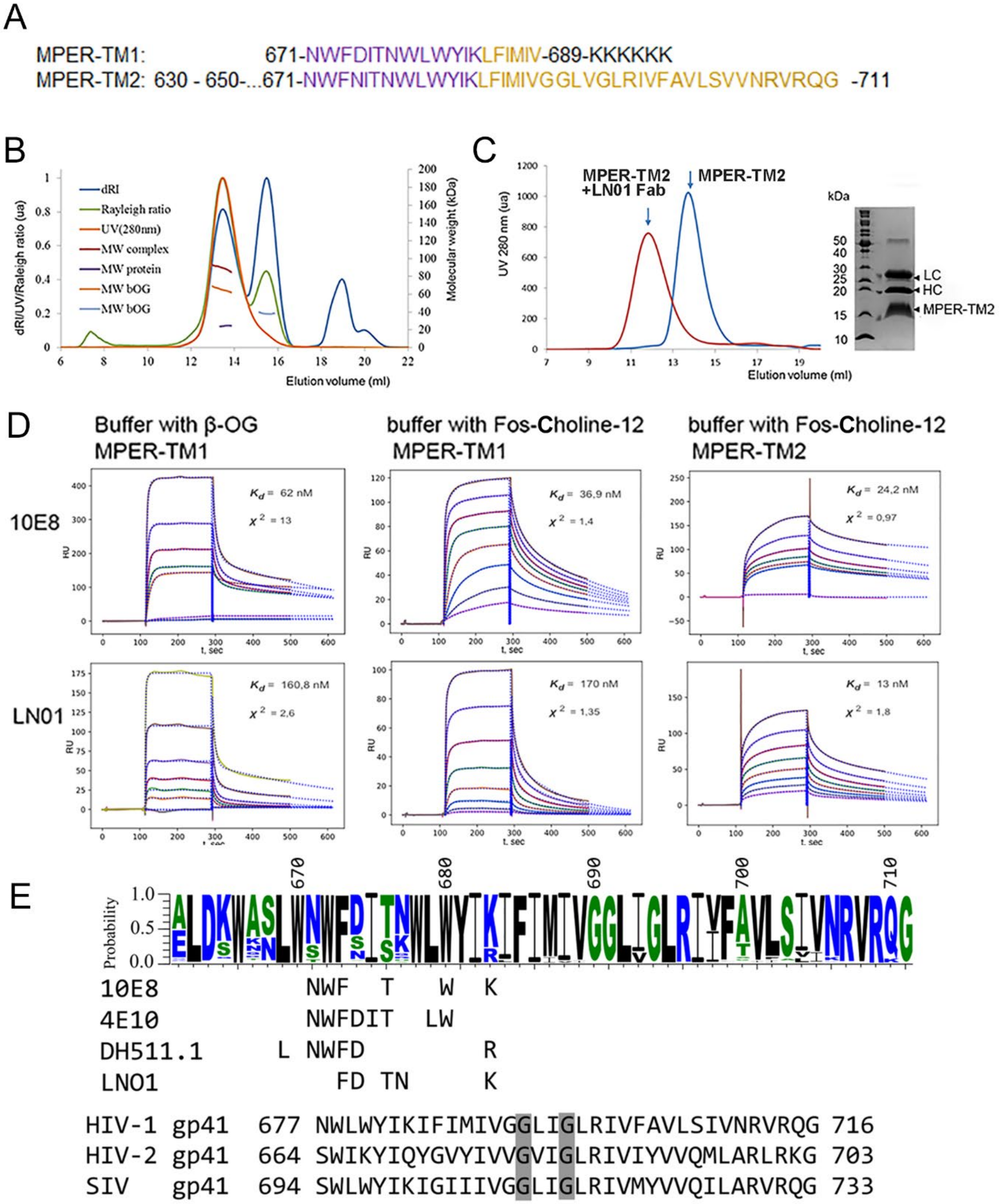

Figure S5 related to Figures 5 and 6. Sequence conservation of MPER and interaction of LN01 with gp41 peptides.

(A) Sequences of gp41 peptides, MPER-TM1 and MPER-TM-2 used in this study. Two forms of MPER-TM2 were expressed, gp41 residues 630 to 711, which was used for experiments

shown in D and MPER-TM2 residues 650 to 711 used for all other MPER-TM2 experiments. MPER-TM2 650 to 711 was generated by an engineered TEV site in the original 630 to 711 construct. The MPER sequence present in the structures is drawn in purple and the transmembrane region in beige.

(B) MPER-TM2 forms trimers in a buffer containing β-OG as determined by SEC-MALLS. The sample was applied to size-exclusion chromatography on a Superdex 200 column. The chromatogram shows detector readings of the light scattering detector (Rayleigh ratio), the UV_{280} detector, and the refractive index detector (dRI). The horizontal lines indicate the calculated mass of MPER-TM2 (24 kDa), detergent micelle in the complex (65 kDa), MPER-TM2-detergent complex (89 kDa) and free detergent micelle (38 kDa). The molecular weight of the MPER-TM2 (residues 650 to 711) monomer is 7.2 kDa.

(C) SEC analysis of MPER-TM2 alone and in complex with LN01 demonstrates complex formation in a buffer containing Fos-Choline-12. MPER-TM2 is monomeric in this buffer. The SDS-PAGE of a central fraction of the LN01-MPER-TM2 peak reveals the presence of MPER-TM2 and the Fab.

(D) SPR analyses of 10E8 and LN01 interaction with MPER-TM1 and MPER-TM2 in buffers containing β-OG and Fos-Choline-12. Injections were performed in the range of 1 to 2048 nM. The raw data is shown in full lines and the fits are drawn as dashed lines. The χ^2 of the fits and the calculated K_d are shown as insets.

(E) Sequence Conservation of gp41 MPER and the transmembrane domain (TM). Upper panel, comparison of 5448 sequences of HIV-1, M group (A-K plus recombinants) from http://www.hiv.lanl.gov. Middle panel, main residues interacting with bNAbs 4E10, 10E8, DH511.1 and LN01. Lower panel, consensus amino acid sequences of the TM region of HIV-1, HIV-2 and SIV showing the positions of the conserved glycine residues.

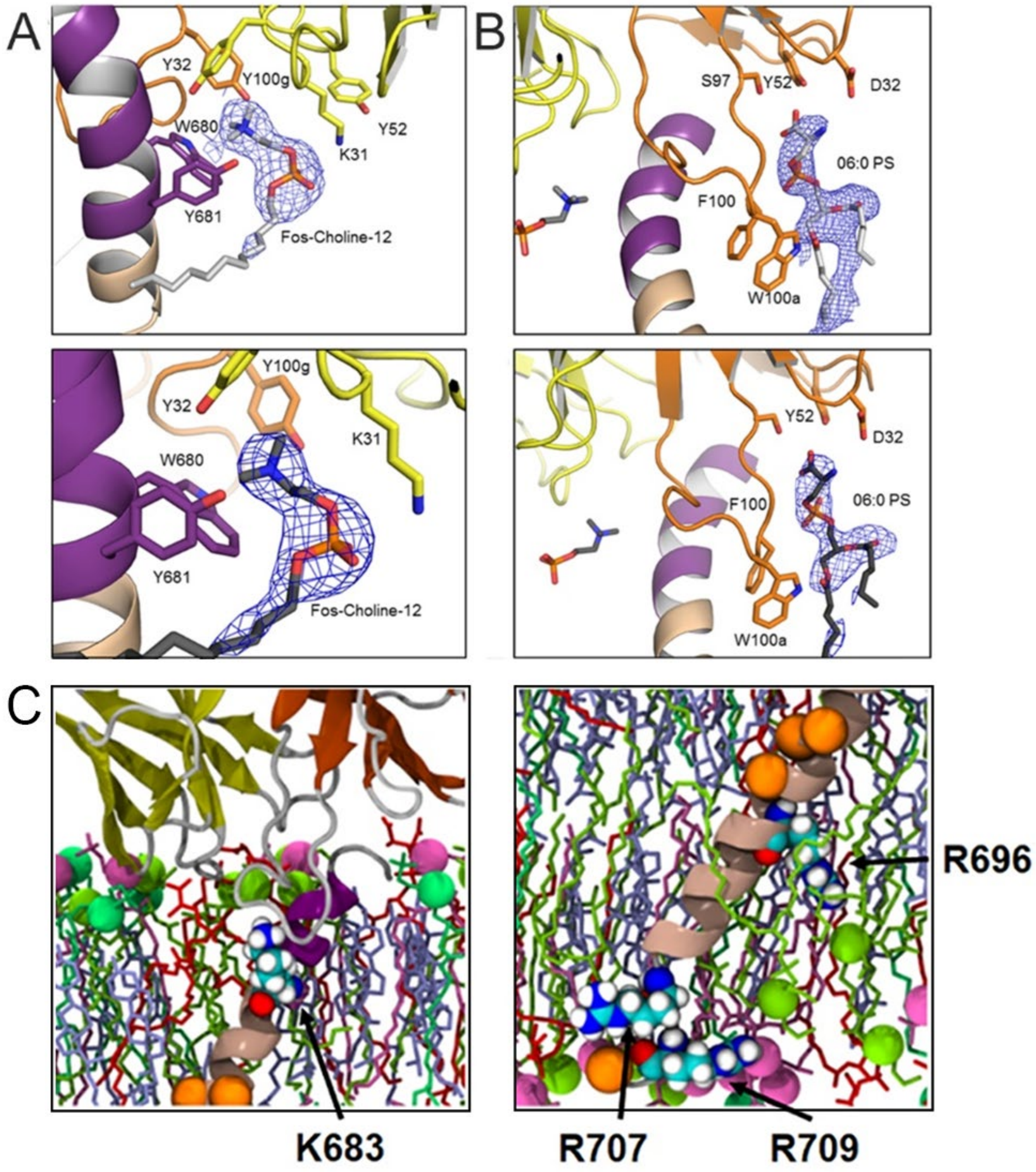

Figure S6 related to Figures 5B, 6 and 7. Molecular details of Fos-Choline-12 and 06:0 PS interaction and of transmembrane residues with lipid head groups. (A) Fos-Choline-12 and (B) 06:0 PS binding. The 2Fo-Fc composite omit map (1 σ level) is represented as a blue mesh around the ligands Fos-Choline-12 (A) and 06:0 PS (B). The 2Fo-Fc map (1σ level) calculated after refinement is represented as a blue mesh around the ligands Fos-Choline-12 (A lower panel) and 06:0 PS (B lower panel). All residues potentially interacting (within 3.5 Å) with the lipids are represented in sticks.

(C) Close-up of the LN01 Fab in complex with MPER-TM2 positioned in a lipid bilayer by MD simulation. The lipid bilayer is composed of POPC (turquoise), POPE (yellow-green), POPS (red), SSM (pink) and cholesterol (mauve). The lipid head groups are represented as van der

Waals spheres of the corresponding color. The light chain of the LN01 Fab is shown in yellow and the heavy chain in orange (left panel). Conserved basic residues positioned to contact lipid head groups on the Ab binding side of the bilayer are shown on the left panel and those present on the on the opposite side of the bilayer are shown on the right panel.

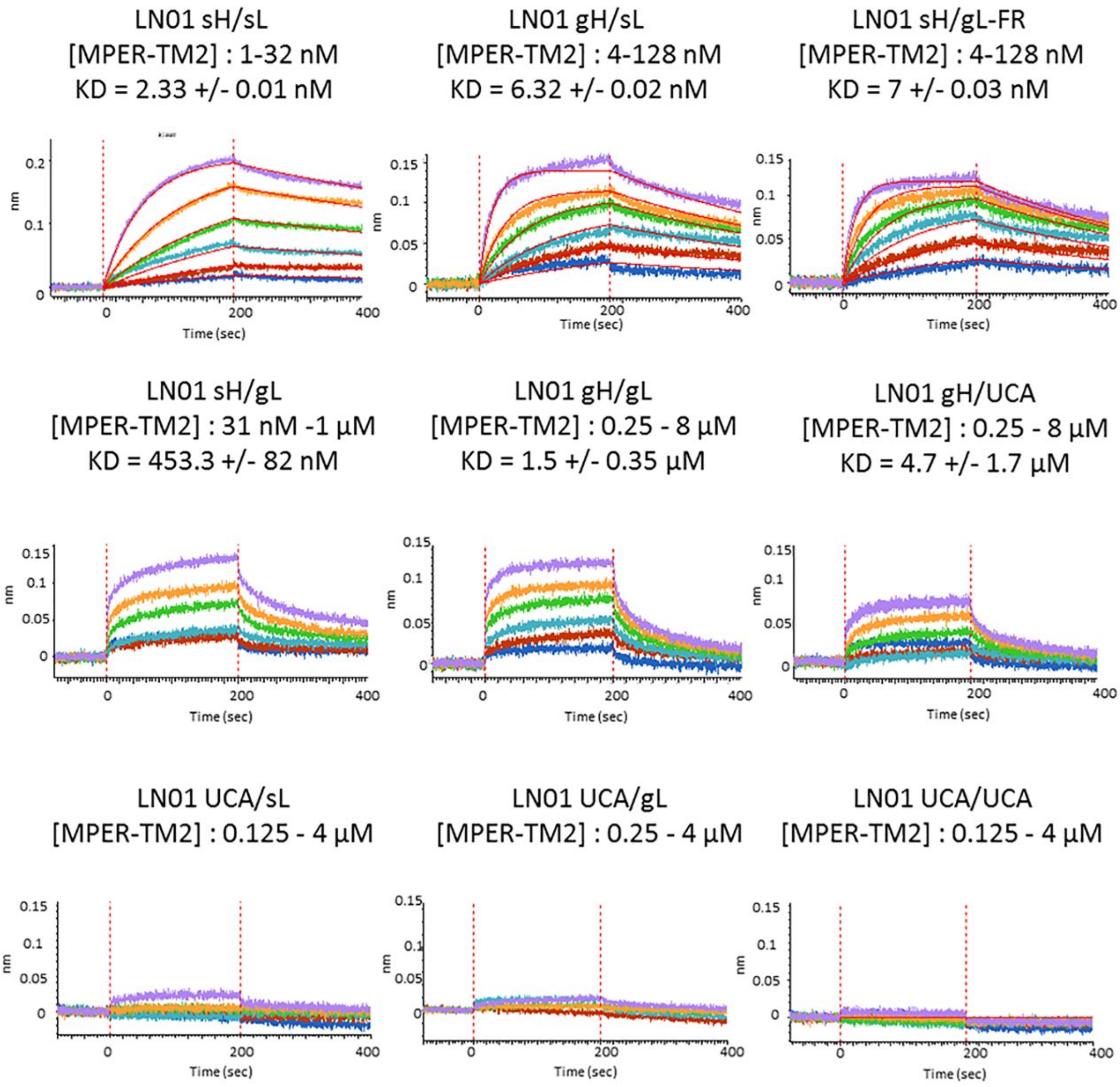

Figure S7 related to Figures 1D and E: Binding of LN01 variants to gp41 MPER-TM2 as measured by Bio-layer interferometry (BLI). Colored lines represent the response curves (blue is the lowest concentration and purple the highest). For LN01 sH/sL, LN01 gH/sL and LN01 sH/gL-FR the red line represents the best fit (1:1 model, see STAR*METHODS).

Table S1 related to Figures 2A, B. Breadth, and potency of LN01 mAb against 118 HIV-1 PVs. LN01 and 10E8 were tested in micro-neutralization assay on TZM-bl target cells against the extended panel of 118 HIV-1 PVs. Shown are the IC50 and IC80 values. Color code: red < 1 µg/ml; orange 1-10 µg/ml; yellow > 10 µg/ml.

		Titer in TZM-bl cells (ug/ml)				
		IC50			IC80	
Virus ID	Clade*	LN01	10E8		LN01	10E8
6535.3	B	1.031	0.081		6.072	1.32
QH0692.42	B	0.515	0.442		4.028	4.046
SC422661.8	B	0.292	0.445		1.446	2.715
PVO.4	B	0.403	0.747		3.793	>10
TRO.11	B	0.185	0.034		0.855	0.250
AC10.0.29	B	1.894	0.132		7.928	1.534
RHPA4259.7	B	10.306	0.728		>25	5.275
THRO4156.18	B	9.831	0.156		>25	0.884
REJO4541.67	B	0.291	0.203		2.261	1.686
TRJO4551.58	B	8.416	0.475		>25	4.783
WITO4160.33	B	0.224	0.094		1.599	1.226
CAAN5342.A2	B	2.242	1.284		18.329	9.281
WEAU_d15_410_5017	B (T/F)	2.471	>10		>25	>10
1006_11_C3_1601	B (T/F)	0.548	0.172		3.885	2.999
1054_07_TC4_1499	B (T/F)	0.092	0.046		0.551	0.305
1056_10_TA11_1826	B (T/F)	0.060	0.134		0.562	1.028
1012_11_TC21_3257	B (T/F)	0.641	0.244		8.756	4.211
6240_08_TA5_4622	B (T/F)	1.978	0.756		14.746	5.591
6244_13_B5_4576	B (T/F)	0.088	0.086		0.445	0.809
62357_14_D3_4589	B (T/F)	5.043	0.119		20.028	1.060
SC05_8C11_2344	B (T/F)	0.710	0.665		4.945	2.736
Du156.12	C	0.146	0.034		0.473	0.144
Du172.17	C	0.059	0.109		0.285	0.837
Du422.1	C	0.929	0.351		6.282	1.883
ZM197M.PB7	C	0.127	0.148		0.977	0.802
ZM214M.PL15	C	0.840	1.962		7.766	>10
ZM233M.PB6	C	0.673	0.442		3.060	2.393
ZM249M.PL1	C	0.944	1.511		4.646	6.294
ZM53M.PB12	C	11.114	3.985		>25	>10
ZM109F.PB4	C	0.618	0.295		4.857	1.888
ZM135M.PL10a	C	0.710	0.171		3.469	1.186
CAP45.2.00.G3	C	1.878	1.817		10.787	>10
CAP210.2.00.E8	C	>25	0.798		>25	3.892
HIV-001428-2.42	C	24.689	0.809		>25	7.298
HIV-0013095-2.11	C	0.028	0.031		0.136	0.213
HIV-16055-2.3	C	0.174	0.722		1.181	6.495
HIV-16845-2.22	C	0.040	0.068		0.326	0.495
Ce1086_B2	C (T/F)	0.328	1.162		2.851	6.934
Ce0393_C3	C (T/F)	>25	1.179		>25	7.423
Ce1176_A3	C (T/F)	1.092	0.867		3.786	3.923
Ce2010_F5	C (T/F)	11.611	1.844		>25	8.612
Ce0682_E4	C (T/F)	0.626	1.215		7.500	7.709
Ce1172_H1	C (T/F)	0.070	0.722		0.472	4.818
Ce2060_G9	C (T/F)	4.050	3.520		12.431	>10
Ce703010054_2A2	C (T/F)	1.703	2.299		8.142	8.579
BF1266.431a	C (T/F)	>25	2.687		>25	>10
246F C1G	C (T/F)	4.894	2.217		15.945	9.937
249M B10	C (T/F)	2.769	1.271		8.389	9.050
ZM247v1(Rev-)	C (T/F)	0.501	0.506		6.465	6.745
7030102001E5(Rev-)	C (T/F)	4.365	6.707		16.490	>10
1394C9G1(Rev-)	C (T/F)	5.964	0.963		23.305	5.327
Ce704809221_1B3	C (T/F)	3.078	0.058		15.769	0.319
CNE19	BC	1.206	0.666		6.774	6.485
CNE20	BC	0.018	1.246		0.050	6.577
CNE21	BC	0.053	1.900		0.317	>10
CNE17	BC	1.112	1.114		5.618	5.255
CNE30	BC	0.597	1.560		2.081	9.842
CNE52	BC	>25	0.304		>25	3.056
CNE53	BC	0.321	0.326		1.982	1.916
CNE58	BC	0.297	1.289		0.831	8.268

		Titer in TZM-bl cells (ug/ml)				
		IC50			IC80	
Virus ID	Clade*	LN01	10E8		LN01	10E8
MS208.A1	A	>25	0.609		>25	3.717
Q23.17	A	5.513	1.637		19.114	7.040
Q461.e2	A	2.829	2.111		11.579	>10
Q769.d22	A	6.420	1.667		21.297	10.000
Q259.d2.17	A	6.498	4.587		>25	>10
Q842.d12	A	7.934	4.189		>25	>10
0330.v4.c3	A	8.243	2.006		>25	>10
0260.v5.c36	A	>25	>10		>25	>10
191955_A11	A (T/F)	1.668	0.680		8.709	3.532
191084 B7-19	A (T/F)	2.614	5.156		22.185	>10
9004SS_A3_4	A (T/F)	0.409	1.160		2.923	6.761
T257-31	CRF02_AG	21.964	0.880		>25	5.779
928-28	CRF02_AG	0.086	0.384		0.573	1.962
263-8	CRF02_AG	0.355	0.196		1.982	1.728
T250-4	CRF02_AG	2.202	1.346		9.990	8.130
T251-18	CRF02_AG	>25	2.357		>25	>10
T278-50	CRF02_AG	5.102	0.949		21.584	7.460
T255-34	CRF02_AG	0.277	0.778		1.838	4.207
211-9	CRF02_AG	0.583	1.091		2.084	6.508
235-47	CRF02_AG	0.874	0.384		3.713	2.072
620345.c01	CRF01_AE	1.547	0.455		13.141	6.002
CNE8	CRF01_AE	0.028	NT		0.259	NT
C1080.c03	CRF01_AE	0.124	0.061		0.653	0.731
R2184.c04	CRF01_AE	0.890	0.272		7.371	2.455
R1166.c01	CRF01_AE	0.603	0.274		3.454	2.614
R3265.c06	CRF01_AE	3.139	>10		>25	>10
C2101.c01	CRF01_AE	0.739	1.921		6.352	8.260
C3347.c11	CRF01_AE	0.901	0.016		2.939	0.167
C4118.c09	CRF01_AE	2.022	1.622		12.752	9.064
CNE5	CRF01_AE	4.345	1.408		17.424	9.637
BJOX009000.02.4	CRF01_AE	1.212	1.570		6.676	9.954
BJOX015000.11.5	CRF01_AE (T/F)	0.529	0.051		8.603	1.909
BJOX010000.06.2	CRF01_AE (T/F)	0.353	0.341		1.605	5.293
BJOX025000.01.1	CRF01_AE (T/F)	0.186	0.107		1.263	6.325
BJOX028000.10.3	CRF01_AE (T/F)	1.097	0.665		18.970	7.361
X1193_c1	G	0.851	0.497		6.106	4.195
P0402_c2_11	G	3.836	0.172		18.720	3.288
X1254_c3	G	3.005	4.753		12.477	>10
X2088_c9	G	>25	>10		>25	>10
X2131_C1_B5	G	0.229	0.189		1.559	0.974
P1981_C5_3	G	0.115	0.057		0.422	0.285
X1632_S2_B10	G	1.846	1.976		7.366	9.684
3016.v5.c45	D	0.250	0.476		1.103	4.393
A07412M1.vrc12	D	0.326	0.576		2.655	6.054
231965.c01	D	9.503	>10		>25	>10
231966.c02	D	0.948	0.191		5.484	3.130
191821_E6_1	D (T/F)	1.239	1.413		4.886	7.737
3817.v2.c59	CD	14.639	1.181		>25	5.977
6480.v4.c25	CD	6.271	8.079		>25	>10
6952.v1.c20	CD	0.168	0.343		1.392	1.907
6811.v7.c18	CD	5.002	7.535		18.990	>10
89-F1_2_25	CD	3.043	1.112		21.607	10.000
3301.v1.c24	AC	7.543	4.628		24.230	>10
6041.v3.c23	AC	1.720	2.333		11.549	9.766
6540.v4.c1	AC	3.885	1.899		19.733	10.000
6545.v4.c1	AC	2.977	1.778		19.229	8.229
0815.v3.c3	ACD	0.384	0.332		5.546	2.868
3103.v3.c10	ACD	>25	>10		>25	>10

* (T/F): Transmitted / Founder Virus

**MPI: Maximum Percent Inhibition

Table S2 related to Figures 5 and 6. Crystallographic data collection and refinement statistics.

**Data collection**	LN01/MPER-TM1*	LN01/MPER-TM2**	LN01/MPER-TM1+06 PS*
Space group	$P\ 4_3\ 2_1\ 2$	$P\ 2_1\ 2_1\ 2_1$	$P\ 4_3\ 2_1\ 2$
Cell dimensions			
a, b, c (Å)	136.76, 136.76, 146.90	65.68, 144.61, 409.50	137.71, 137.71, 146.72
α, β, γ (°)	90, 90, 90	90, 90, 90	90, 90, 90
Resolution (Å)	47.00-3.20 (3.31-3.20) [1]	49.63-3.90 (4.09-3.90) [1]	48.91-3.10 (3.31-3.10)[1]
Unique reflexions	23542 (2291) [1]	35232 (3519) [1]	26213 (4662) [1]
R_{merge}[2]	0.088 (1.235) [1]	0.144 (0.555) [1]	0.161 (0.968) [1]
R_{pim}[3]	0.029 (0.395) [1]	0.073 (0.279) [1]	0.068 (0.415) [1]
$I / \sigma I$	21.6 (1.7) [1]	7.0 (2.7) [1]	8.8 (1.9) [1]
Completeness (%)	99.69 (99.48) [1]	96.2 (97.7) [1]	99.8 (99.8) [1]
Redundancy	9.6 (9.9) [1]	4.2 (4.3) [1]	7.1 (7.1) [1]
CC (1/2)	0.999 (0.688) [1]	0.995 (0.776) [1]	0.995 (0.671)
**Refinement**			
Resolution (Å)	47.00 - 3.20 (3.51 - 3.20) [1]	48.64 - 3.9 (4.039 - 3.9) [1]	46.21 - 3.1 (3.211 - 3.1) [1]
No. reflections	23526 (2285) [1]	35206 (3518) [1]	26131 (2565) [1]
Reflections used for R_{free}[5]	1208 (117) [1]	1726 (174) [1]	1336 (124) [1]
R_{work}[4] / R_{free}[5]	0.213 / 0.253	0.218/0.260	0.238/0.267
No. atoms			
Protein	7090	14832	7118
Ligand/ion	83	166	92
Water	0	0	0
Wilson B (Å^2)	85	105	77
Average B-factors (Å^2)			
Overall	96	124	69
Protein	96	124	69
Ligand/ion	137	137	81
Water			
R.m.s deviations			
Bond lengths (Å)	0.010	0.007	0.004
Bond angles (°)	1.47	1.17	1.15
Ramachandran Plot (%)			
Favoured	96.22	95.39	95.80
Outliers	0.33	0.85	0.44
PDB ID	6SNC	6SNE	6SND

*1 crystal was used for the structure; **2 crystals was used for the structure; [1] Statistics for the highest resolution shell.

[1] Parentheses refer to outer shell statistics.

[2] $R_{merge} = \Sigma_{hkl} \Sigma_i \mid I_{hkl,i} - \langle I_{hkl} \rangle \mid / \Sigma_{hkl} \Sigma_i I_{hkl,i}$, where $I_{hkl,i}$ is the scaled intensity of the ith measurement of
reflection h, k, l, and $\langle I_{hkl} \rangle$ is the average intensity for that reflection.

[3] $R_{pim} = \Sigma_{hkl} (1/(n-1))^{1/2} \Sigma_i \mid I_{hkl,i} - \langle I_{hkl} \rangle \mid / \Sigma_{hkl} \Sigma_i I_{hkl,i}$, where n is the number of time a given reflexion has been measured.

[4] $R_{work} = \Sigma_{hkl} \mid Fo - Fc \mid / \Sigma_{hkl} \mid Fo \mid \times 100$, where Fo and Fc are the observed and calculated structures factors.

[5] R_{free} was calculated as for R_{work}, but on a test set of 5% of the data excluded from refinement.

Table S3 related to Figure 5. Contact residues between gp41 and LN01. The contact distances (cut-off 3.5 Å) were calculated based on the LN01/MPER-TM1+06:0 PS structure.

gp41			LN01			Contact	
Residue name, number , interacting atom			Residue name, number, interacting atom			residue distance (A)	Hydrogen bond
Asn	671	ND2	Tyr	H 58	CD1	3.3	
Phe	673	CD1	Trp	H 100h	CE2	3.4	
Phe	673	CD2	Trp	L 96	CH2	3.5	
Phe	673	CE2	Thr	H 50	CG2	3.4	
Asp	674	OD1	Ser	L 93	C	3.5	
			Thr	L 94	CA	3.3	
			Thr	L 94	N	3.2	***
Asp	674	OD2	Thr	L 94	CB	3.1	
Thr	676	O	Ser	H 100f	CB	3.3	
Thr	676	OG1	Trp	H 100h	NE1	3.0	***
Asn	677	OD1	Tyr	L 32	CE2	3.2	
			Tyr	L 32	CZ	3.5	
Asn	677	ND2	His	L 92	ND1	3.2	***
			His	L 92	O	2.9	***
Trp	680	CD1	Phe	H 100e	O	3.2	
			Ser	H 100f	OG	3.3	
Lys	683	CD	Phe	H 100	O	3.3	
Lys	683	CE	Phe	H 100	O	3.1	
Lys	683	NZ	Phe	H 100	O	2.5	***
			Trp	H 100a	CA	3.4	
			Trp	H 100a	C	3.4	
			Ser	H 100b	O	3	***
Met	687	SD	Trp	H 100a	CZ3	3.4	

H, Ab heavy chain; L, Ab light chain
